# Supplementary material for: An autocrine ActivinB mechanism drives TGFβ/Activin signaling in Group 3 medulloblastoma
Source: EMBO Mol Med. 2019 Jul 22;11(8):e9830. doi: 10.15252/emmm.201809830 (PMC6685082; doi:10.15252/emmm.201809830)
Supplement: Supplementary file 1 — Appendix [file EMMM-11-e9830-s001.pdf]

An autocrine ActivinB mechanism drives TGFβ/Activin signaling in Group 3 medulloblastoma.

Morgane Morabito, Magalie Larcher, Florence M.G. Cavalli, Chloé Foray, Antoine Forget, Liliana Mirabal-Ortega, Mamy Jean De Dieu Andrianteranagna, Sabine Druillennec, Alexandra Garancher, Julien Masliah-Planchon, Sophie Leboucher, Abel Debalkew, Alessandro Raso, Olivier Delattre, Stéphanie Puget, François Doz, Michael D. Taylor, Olivier Ayrault, Franck Bourdeaut, Alain Eychène, Celio Pouponnot

Correspondence:
Celio POUPONNOT PhD
Institut Curie - Recherche
Laboratoire 110, Centre Universitaire
91405 - ORSAY Cedex, FRANCE
E-mail : celio.pouponnot@curie.fr
Tel. 33-1 69 86 30 79
Fax. 33-1 69 86 30 51

Running Title: ActivinB in Group3 medulloblastoma

Key words: Medulloblastoma, Activin, TGFbeta, Smad2, Smad3

APPENDIX

Table of content

Appendix Figure S1 2
Pharmacological inhibitors and blocking antibody specificity

Appendix Figure S2 3
ActivinB stimulation does not promote growth in HDMB03 cells

Appendix Figure S3 4
PMEPA1 is a target gene of Activin signaling

Appendix Figure S4 5
TGFβ/ActivinB signaling in group 3 MB PDX

Appendix Figure S5 6
P-Smad2/Total Smad2 normalization

Appendix Table S1 7
Statistics related to Figure 1B, 5B-C and EV1B

Appendix Table S2 8
Statistics related to Figure 1D, 6B and EV1D and Appendix Figure S4A

Appendix Table S3 10
Statistics related to Figure 7D-E

Appendix Table S4 13
Oligonucleotides used in this study for RT-qPCR experiments

Appendix Table S5 14
Exact p-values and number of replicates for each experiment

Appendix Supplemental Figure legends 20

Appendix Supplemental Table legends 22

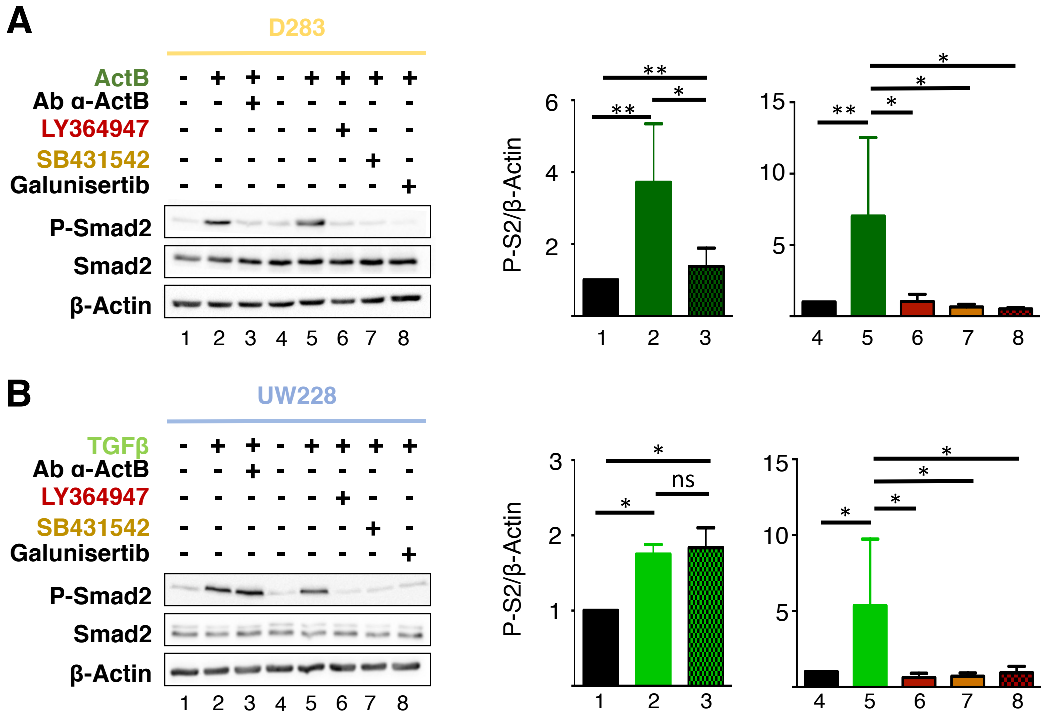

Appendix Figure S1 Morabito et al.

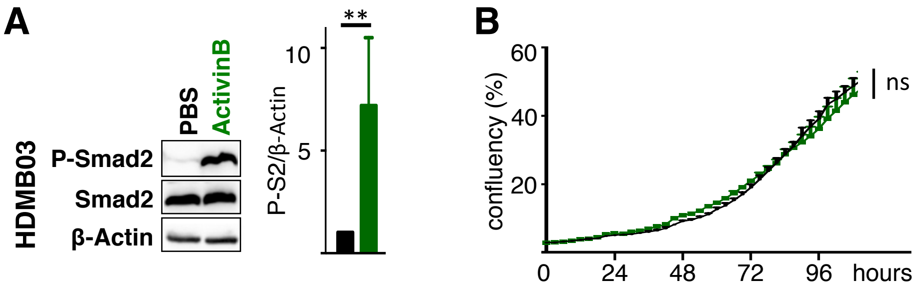

Appendix Figure S2 Morabito et al.

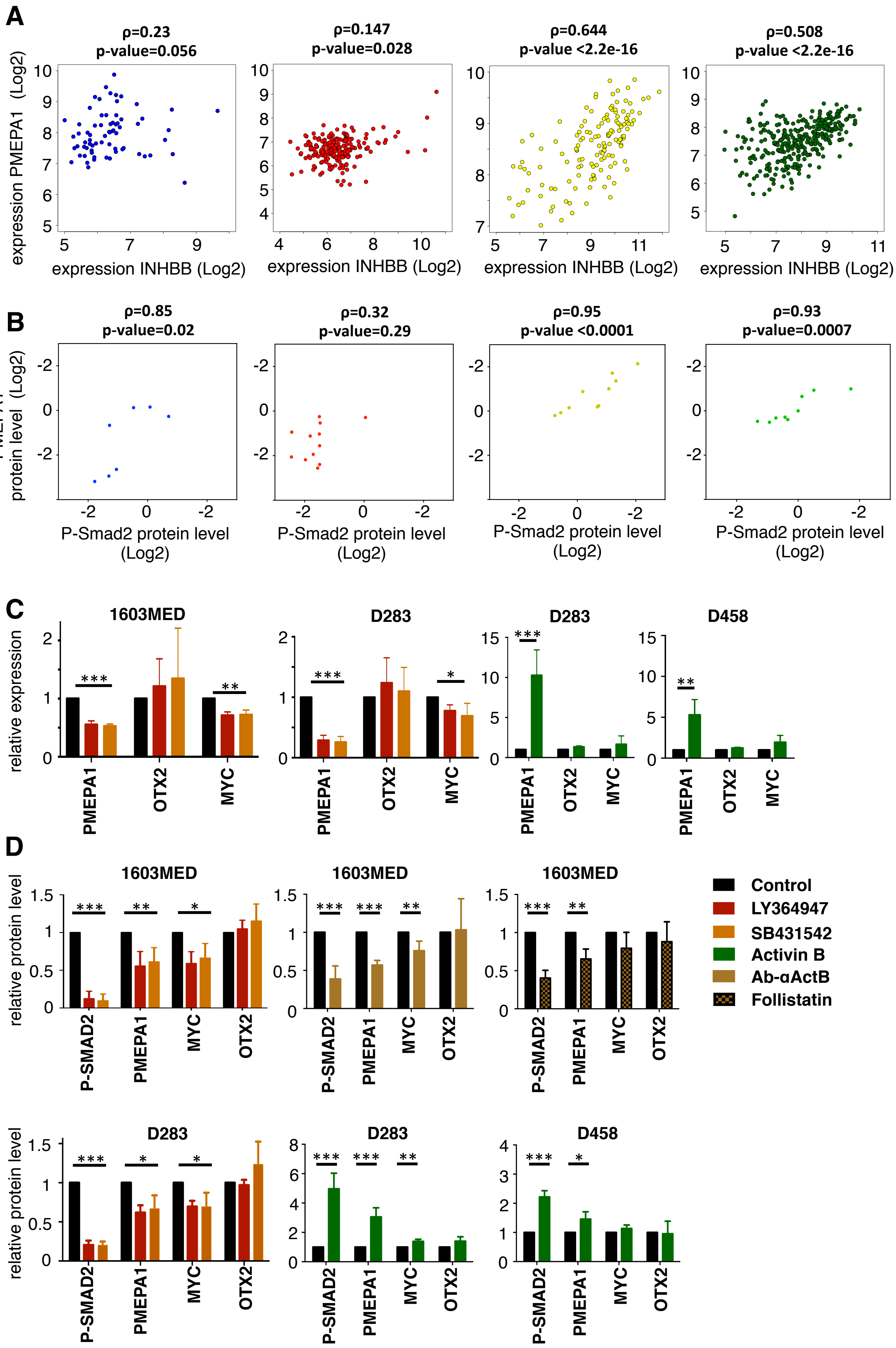

Appendix Figure S3 Morabito et al.

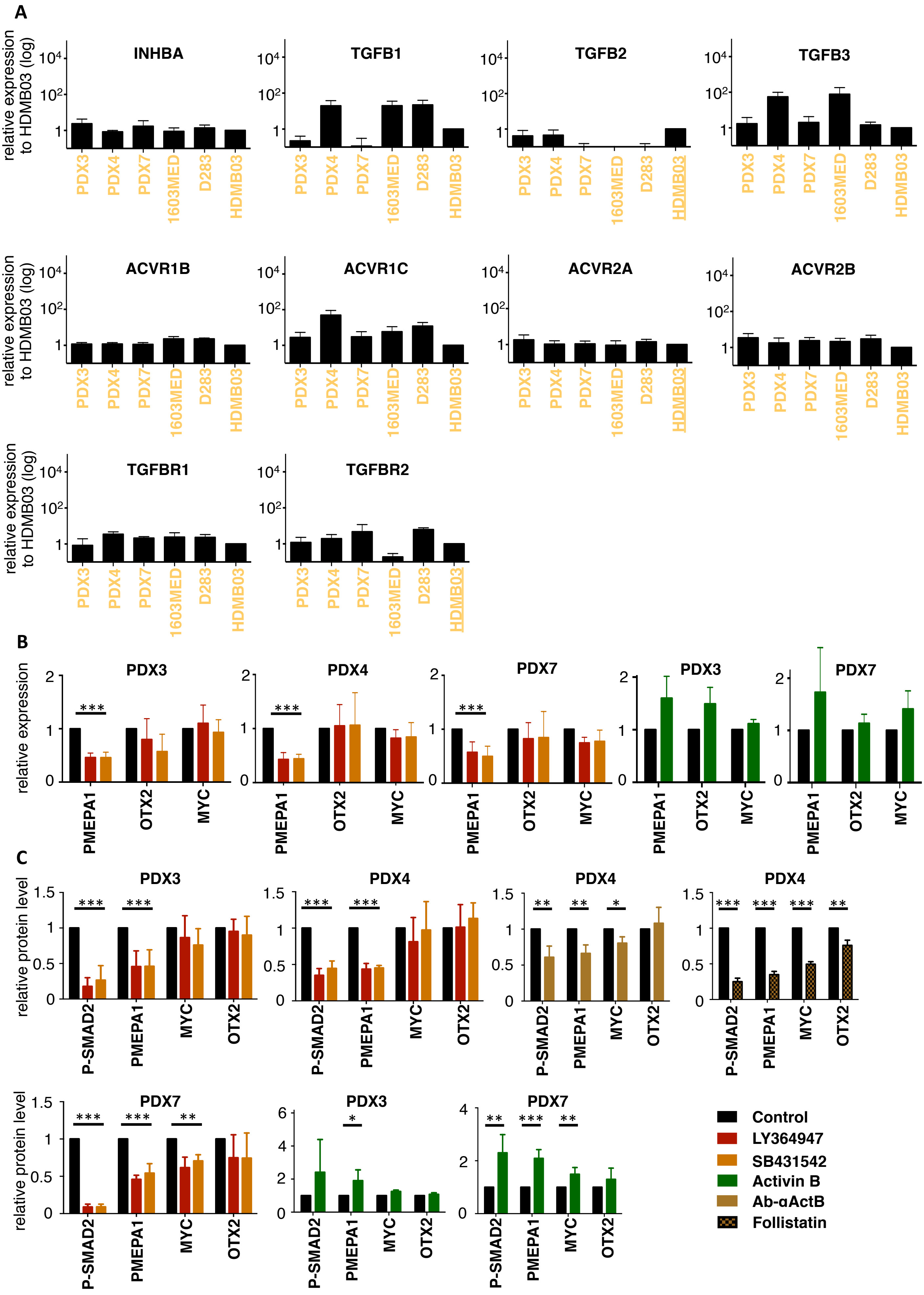

Appendix Figure S4 Morabito et al.

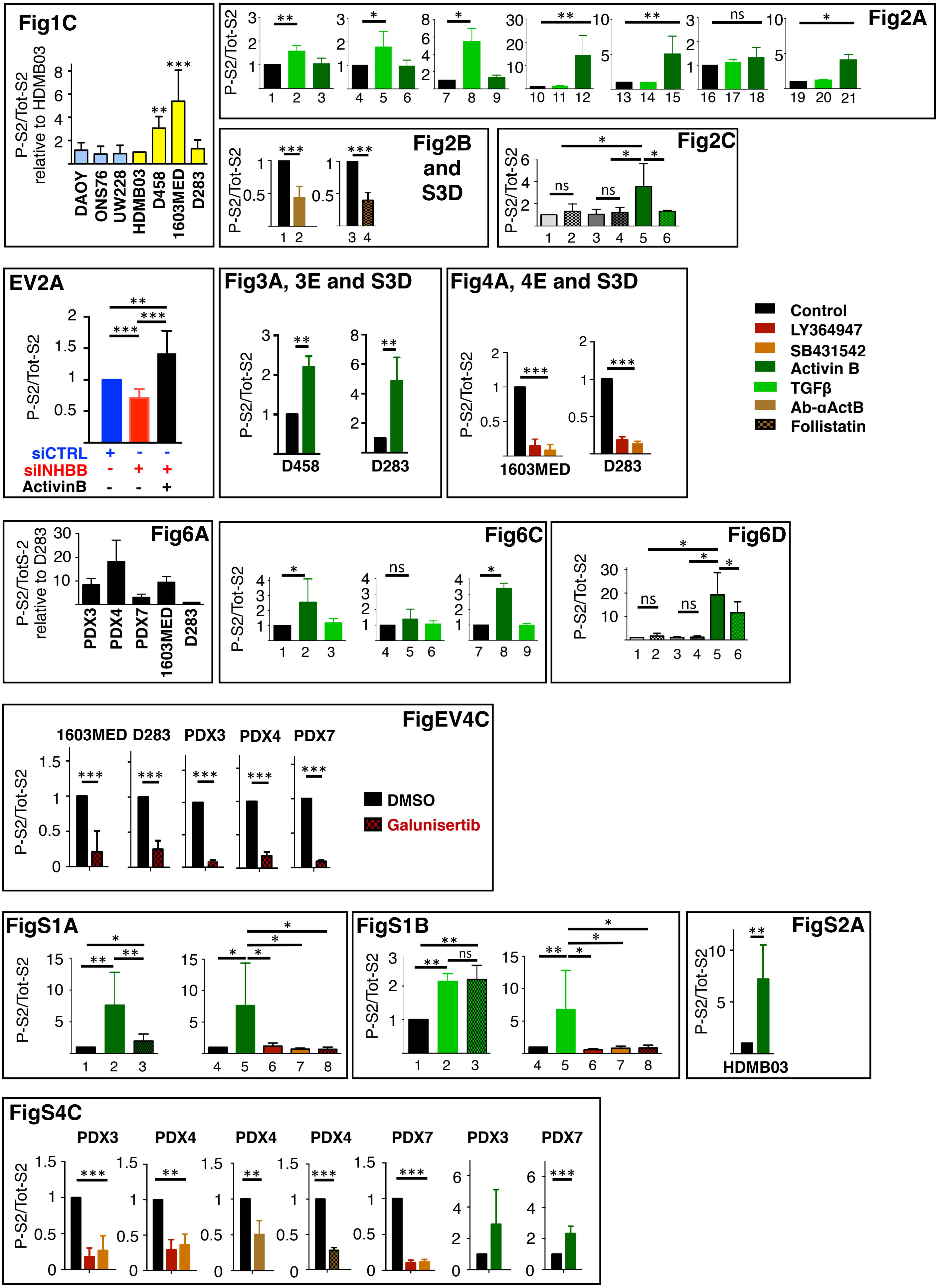

Appendix Figure S5 Morabito et al.

Appendix Table S1:Statistics related to Figure 1B, 5B-C and EV1B-C

EnsemblGeneID_from_ensemblv77	HGNC_symbol_from_ensemblv77	Fetal_vs_Adult	Fetal_vs_WNT	Fetal_vs_SHH	Fetal_vs_Group3	Fetal_vs_Group4	Adult_vs_WNT	Adult_vs_SHH	Adult_vs_Group3	Adult_vs_Group4	WNT_vs_SHH	WNT_vs_Group3	WNT_vs_Group4	SHH_vs_Group3	SHH_vs_Group4	Group3_vs_Group4
ENSG00000115170	ACVR1	3,884E-01	1,612E-05	6,816E-01	2,037E-05	1,028E-06	2,430E-04	6,780E-01	2,630E-04	5,034E-05	4,800E-21	2,229E-03	4,420E-13	5,162E-36	1,790E-70	8,397E-08
ENSG00000135503	ACVR1B	1,758E-02	8,258E-02	1,912E-01	5,173E-01	2,695E-01	4,618E-02	1,376E-02	1,798E-03	9,235E-03	2,350E-01	3,459E-06	1,040E-01	9,013E-07	4,947E-01	5,530E-06
ENSG00000123612	ACVR1C	3,996E-04	9,815E-01	8,410E-02	7,331E-04	1,086E-03	5,447E-05	2,961E-04	1,488E-04	3,745E-05	5,568E-05	3,093E-12	2,650E-13	1,272E-03	5,893E-03	5,086E-02
ENSG00000121989	ACVR2A	2,238E-01	5,610E-03	4,118E-01	8,875E-02	9,503E-02	2,141E-01	3,162E-01	2,641E-03	7,426E-01	1,537E-07	3,003E-17	1,415E-04	2,047E-12	4,944E-03	5,338E-22
ENSG00000114739	ACVR2B	7,592E-03	8,258E-02	2,736E-05	8,103E-06	1,361E-04	1,747E-03	1,412E-04	5,978E-05	7,374E-05	8,417E-17	7,595E-16	9,334E-09	9,321E-01	1,241E-11	6,276E-09
ENSG00000105329	TGFB1	5,287E-01	6,769E-03	4,626E-01	3,768E-05	5,878E-03	4,772E-03	1,315E-01	2,099E-04	2,043E-03	1,823E-08	1,502E-06	1,507E-01	1,123E-33	2,807E-14	7,348E-19
ENSG00000092969	TGFB2	3,996E-04	1,315E-06	7,381E-01	7,385E-07	3,113E-07	3,308E-03	8,260E-04	2,021E-04	5,128E-05	3,763E-31	8,823E-02	4,705E-04	1,431E-52	5,499E-83	5,104E-02
ENSG00000119699	TGFB3	3,596E-02	1,173E-01	2,294E-02	9,169E-04	8,151E-04	2,609E-02	5,934E-01	7,752E-04	4,722E-04	2,240E-12	1,771E-06	6,814E-05	5,093E-37	1,697E-53	1,557E-02
ENSG00000106799	TGFBR1	6,070E-01	1,217E-06	4,208E-03	4,854E-06	2,178E-06	5,447E-05	1,750E-02	1,546E-04	1,468E-04	2,216E-21	7,840E-01	1,134E-18	1,035E-24	3,682E-10	4,587E-18
ENSG00000163513	TGFBR2	6,070E-01	4,731E-01	6,637E-04	2,370E-01	3,435E-01	5,063E-01	4,033E-03	4,014E-01	3,895E-01	8,106E-13	4,297E-03	1,145E-01	1,966E-26	1,175E-13	4,002E-07
ENSG00000123999	INHA	2,557E-02	9,446E-01	1,651E-01	4,637E-01	2,680E-01	1,563E-02	1,078E-01	6,213E-02	8,253E-02	7,322E-03	2,297E-01	2,855E-02	1,503E-01	3,706E-01	3,845E-01
ENSG00000163083	INHBB	3,996E-04	1,441E-03	6,395E-04	7,069E-05	3,719E-03	9,309E-01	9,328E-01	2,941E-04	6,805E-04	6,397E-01	3,310E-24	2,190E-20	1,811E-42	7,130E-45	3,294E-21
ENSG00000175189	INHBC	3,277E-01	2,440E-01	1,086E-03	1,062E-02	5,603E-06	1,257E-01	4,278E-03	1,629E-02	2,661E-04	4,829E-07	2,265E-03	2,794E-21	1,995E-02	3,694E-16	4,588E-19
ENSG00000139269	INHBE	1,810E-01	2,476E-02	3,598E-02	4,926E-01	9,624E-01	2,850E-01	4,010E-01	6,915E-02	3,564E-02	6,009E-01	2,229E-03	1,239E-06	5,082E-06	4,354E-14	4,792E-02
ENSG00000124225	PMEPA1	4,955E-02	5,112E-05	9,011E-05	1,014E-06	1,510E-02	4,082E-04	3,673E-04	5,511E-05	4,226E-03	7,182E-03	1,931E-08	2,922E-05	1,842E-31	1,533E-02	1,003E-37

HGNC_symbol_from_ensemblv77	WNT_vs_SHH	WNT_vs_Group3	WNT_vs_Group4	SHH_vs_Group3	SHH_vs_Group4	Group3_vs_Group4	Group3_vs_WNT_and_SHH_and_Group4	Group3_alpha_vs_Group3_beta	Group3_alpha_vs_Group3_gamma	Group3_beta_vs_Group3_gamma	Group3_alpha_vs_.Group3_beta_and_gamma
ENSG00000124225	PMEPA1	0,0071818	1,93E-08	2,92E-05	1,84E-31	0,0153323	1,00E-37	1,16E-39	1,38E-08	3,33E-05	0,097734959
ENSG00000129535	NRL	3,29E-24	4,96E-25	0,933501491	9,73E-58	5,68E-41	5,41E-35	1,63E-54	5,69E-09	4,74E-09	0,843313181
ENSG00000136997	MYC	1,05E-35	0,0458677	2,46E-32	6,31E-38	0,7007526	8,34E-37	2,21E-34	1,04E-08	6,48E-15	1,89E-06
ENSG00000163083	INHBB	0,6396935	3,31E-24	2,19E-20	1,81E-42	7,13E-45	3,29E-21	5,33E-38	0,000591146	0,005146791	0,780422424

Statistics related to transcriptomic analyses on data set from Cavalli et al. (Cancer Cell. 2017; 31(6):737-754.e6. doi: 10.1016/j.ccell.2017.05.005.)

Column ID	p-value(G3 vs. G4)	p-value(G3 vs. SHH)	p-value(G3 vs. WNT)	p-value(G3 vs. G4 and SHH and WNT)	p-value(G4 vs. SHH)	p-value(G4 vs. WNT)	p-value(SHH vs. WNT)	p-value(Group Fused)
TGFB1	5.35963e-005	5.67015e-005	0.238964	0.000313135	0.858953	0.135101	0.158415	9.4332e-005
TGFB2	0.247443	1.12487e-006	0.75613	0.165101	8.55297e-008	0.734319	0.00134171	2.20759e-007
TGFB3	0.00180916	7.93946e-007	0.0601249	7.10596e-005	0.0259683	0.931655	0.148734	1.48278e-005
INHBA	0.0439173	0.00915805	9.87976e-005	0.0228808	0.359509	5.67716e-005	3.20652e-005	0.000161164
INHBB	0.0097442	0.00697344	0.516205	0.0231563	0.999634	0.389553	0.382804	0.0232013
ACVR1B	0.116212	0.55153	0.722947	0.895126	0.0313741	0.219181	0.975419	0.150793
ACVR2A	0.188444	0.104717	0.0206685	0.0158543	0.79032	0.12028	0.155643	0.0946167
ACVR1C	0.750199	0.30014	0.846786	0.548559	0.473682	0.99339	0.648938	0.75337
TGFBR2	0.0604814	0.0676761	0.547578	0.086931	0.881089	0.369556	0.416615	0.186227

Statistics related to proteomic analyses

Appendix Table S2: statistics related to Figure 1D, 6B and EV1D and Appendix Figure S4A

**TGFBR1 - ANOVA**	pvalue	
non group3 vs. group3 cell lines	0,011	Fig EV1
non group3 cell lines vs. group3 PDX	0,0077	
group3 cell lines vs. group3 PDX	0,6685	Appendix Figure S4

**TGFBR2 - ANOVA**	pvalue	
non group3 vs. group3 cell lines	< 0,0001	Fig EV1
non group3 cell lines vs. group3 PDX	< 0,0001	
group3 cell lines vs. group3 PDX	0,9714	Appendix Figure S4

**ACVR2A - ANOVA**	pvalue	
non group3 vs. group3 cell lines	0,0147	Fig EV1
non group3 cell lines vs. group3 PDX	0,0038	
group3 cell lines vs. group3 PDX	0,5366	Appendix Figure S4

**ACVR2B - ANOVA**	pvalue	
non group3 vs. group3 cell lines	0,0003	Fig EV1
non group3 cell lines vs. group3 PDX	0,0009	
group3 cell lines vs. group3 PDX	0,762	Appendix Figure S4

**ACVR1B - ANOVA**	pvalue	
non group3 vs. group3 cell lines	< 0,0001	Fig EV1
non group3 cell lines vs. group3 PDX	< 0,0001	
group3 cell lines vs. group3 PDX	< 0,0001	Appendix Figure S4

**ACVR1C - ANOVA**	pvalue	
non group3 vs. group3 cell lines	0,7806	Fig EV1
non group3 cell lines vs. group3 PDX	0,0623	
group3 cell lines vs. group3 PDX	0,049	Appendix Figure S4

**INHBA - ANOVA**	pvalue	
non group3 vs. group3 cell lines	0,0038	Fig EV1
non group3 cell lines vs. group3 PDX	0,0184	
group3 cell lines vs. group3 PDX	0,9993	Appendix Figure S4

INHBB - ANOVA	pvalue	
non group3 vs. group3 cell lines	0,2775	Figure 1
non group3 cell lines vs. group3 PDX	0,1975	
group3 cell lines vs. group3 PDX	0,6649	Appendix Figure S4

INHBB - non parametric t-test	pvalue	
MED1603 vs. non group3	0,0016	Figure 1
MED1603 vs. D283	0,0079	Figure 1
D283 vs. non group3	0,0016	Figure 1
HDMB03 vs. non group3	> 0,9999	Figure 1
D458 vs. non group3	0,0917	Figure 1

TGFB1 - ANOVA	pvalue	
non group3 vs. group3 cell lines	0,6167	Fig EV1
non group3 cell lines vs. group3 PDX	0,0949	
group3 cell lines vs. group3 PDX	0,1494	Appendix Figure S4

TGFB2 - ANOVA	pvalue	
non group3 vs. group3 cell lines	< 0,0001	Fig EV1
non group3 cell lines vs. group3 PDX	< 0,0001	
group3 cell lines vs. group3 PDX	0,9982	Appendix Figure S4

TGFB3 - ANOVA	pvalue	
non group3 vs. group3 cell lines	0,5274	Figure 1
non group3 cell lines vs. group3 PDX	0,3898	
group3 cell lines vs. group3 PDX	0,7561	Appendix Figure S4

TGFB3 - non parametric t-test	pvalue	
MED1603 vs. non group3	0,029	Figure 1
MED1603 vs. D283	0,0079	Figure 1
D283 vs. non group3	0,7443	Figure 1
HDMB03 vs. non group3	0,7483	Figure 1
D458 vs. non group3	0,2817	Figure 1

Statistics related to RT-qPCR experiments assessing expression levels of the TGFβ/ActivinB signaling pathway mediators performed in cell lines and PDX

Appendix Table S3 : Statistics related to Figure 7D-E

WNT_alpha

	PMEPA1	NRL	MYC	INHBB
PMEPA1	1	0,30255102	-0,147142857	0,238979592
NRL	0,30255102	1	0,101938776	-0,060102041
MYC	-0,147142857	0,101938776	1	-0,091326531
INHBB	0,238979592	-0,060102041	-0,091326531	1

WNT_beta

	PMEPA1	NRL	MYC	INHBB
PMEPA1	1	0,179220779	0,437662338	0,185714286
NRL	0,179220779	1	0,546753247	0,227272727
MYC	0,437662338	0,546753247	1	0,402597403
INHBB	0,185714286	0,227272727	0,402597403	1

SHH_alpha

	PMEPA1	NRL	MYC	INHBB
PMEPA1	1	0,046940559	0,22736014	0,145847902
NRL	0,046940559	1	0,089554196	0,102797203
MYC	0,22736014	0,089554196	1	0,340078671
INHBB	0,145847902	0,102797203	0,340078671	1

SHH_beta

	PMEPA1	NRL	MYC	INHBB
PMEPA1	1	0,013445378	-0,078711485	0,336414566
NRL	0,013445378	1	-0,220728291	0,060784314
MYC	-0,078711485	-0,220728291	1	0,125210084
INHBB	0,336414566	0,060784314	0,125210084	1

SHH_gamma

	PMEPA1	NRL	MYC	INHBB
PMEPA1	1	-0,062095282	0,041859389	0,068917669
NRL	-0,062095282	1	0,142807586	-0,143038853
MYC	0,041859389	0,142807586	1	0,473982424
INHBB	0,068917669	-0,143038853	0,473982424	1

SHH_delta

	PMEPA1	NRL	MYC	INHBB
PMEPA1	1	-0,073766234	-0,020041012	0,191442242
NRL	-0,073766234	1	0,034531784	0,049405332
MYC	-0,020041012	0,034531784	1	0,159289132
INHBB	0,191442242	0,049405332	0,159289132	1

Group3_alpha

	PMEPA1	NRL	MYC	INHBB
PMEPA1	1	-0,204725038	0,235174395	0,618844281
NRL	-0,204725038	1	-0,074068162	-0,087437146
MYC	0,235174395	-0,074068162	1	0,300782185
INHBB	0,618844281	-0,087437146	0,300782185	1

Group3_beta

	PMEPA1	NRL	MYC	INHBB
PMEPA1	1	-0,076813656	0,1628734	0,404931247
NRL	-0,076813656	1	-0,183499289	0,057373163
MYC	0,1628734	-0,183499289	1	0,257230915
INHBB	0,404931247	0,057373163	0,257230915	1

Group3_gamma

	PMEPA1	NRL	MYC	INHBB
PMEPA1	1	0,169043152	-0,181050657	0,650469043
NRL	0,169043152	1	-0,416322702	0,428705441
MYC	-0,181050657	-0,416322702	1	-0,166041276
INHBB	0,650469043	0,428705441	-0,166041276	1

Group4_alpha

	PMEPA1	NRL	MYC	INHBB
PMEPA1	1	0,145853655	0,143188672	0,314729453
NRL	0,145853655	1	0,149704493	0,362469636
MYC	0,143188672	0,149704493	1	-0,044546028
INHBB	0,314729453	0,362469636	-0,044546028	1

Group4_beta

	PMEPA1	NRL	MYC	INHBB
PMEPA1	1	0,280530071	0,455472153	0,445436011
NRL	0,280530071	1	0,11225095	0,251617088
MYC	0,455472153	0,11225095	1	0,208655361
INHBB	0,445436011	0,251617088	0,208655361	1

Group4_gamma

	PMEPA1	NRL	MYC	INHBB
PMEPA1	1	0,056167213	0,398632673	0,451830224
NRL	0,056167213	1	0,011551061	0,117426293
MYC	0,398632673	0,011551061	1	0,14766415
INHBB	0,451830224	0,117426293	0,14766415	1

INHBB-NRL

Group	cor (spearman)	p-value
WNT	0,042183536	0,728234249
SHH	0,019611259	0,770696687
Group3	0,302829355	0,000239812
Group4	0,514738041	0
G3a	-0,087437146	0,480779646
G3b	0,057373163	0,735153304
G3g	0,428705441	0,006173715

Spearman correlation (ρ and p-values) related to the analyses performed on data set from Cavalli et al. (Cancer Cell. 2017; 31(6):737-754.e6. doi: 10.1016/j.ccell.2017.05.005.) for *INHBB, PMEPA1, MYC* and *NRL*.

Appendix Table S4

gene	Forward (F)/Reverse (R)	sequences (human)
ACVR1B	F	CTGTTCCCACTCAGCAGTGA
	R	AAAGGGCCCTAGAGCAAAAA
ACVR1C	F	TGCCACAATATCGGGTACAA
	R	CTCTTCCCCCACACACTCAC
ACVR2A	F	ACACAGCCCACTTCAAATCC
	R	AGGAGGGTAGGCCATCTTGT
ACVR2B	F	CACTGGCAGACTGGAGTCAA
	R	TCCTCCCAATCCACAAGAAG
GAPDH	F	GGTCTCCTCTGACTTCAACA
	R	AGCCAAATTCGTTGTCATAC
INHBA	F	CTTGAGGTTCCCTTGTGAGC
	R	GGGTAGGAAAGTGCCTGTGA
INHBB	F	GTGACAGGTGGAACATGGTG
	R	TGCACGTCTAGGTTGAGTCG
MYC	F	GAGGCTATTCTGCCCATTTG
	R	AGGCTGCTGGTTTTCCACTA
OTX2	F	CCCACTGTCAGATCCCTTGT
	R	ATGCCCCCAAAGTAGGAAGT
PMEPA1	F	TTTCCATCTCCTTTCCCCGC
	R	CCCGCCAACCCCAAATCTAT
TGFB1	F	CAACAATTCCTGGCGATACC
	R	CTAAGGCGAAAGCCCTCAAT
TGFB2	F	ATCCCCTGTAGCCCCATAAC
	R	GGCAAGTAGCTGATCCCAAA
TGFB3	F	CCTCTCTTCCCAGCTCACAC
	R	AATGGCTTCCACCCTCTTCT
TGFBRI	F	CAGCTCTGGTTGGTGTCAGA
	R	ATGTGAAGATGGGCAAGACC
TGFBRII	F	TTTTGTTTGGTCAGCACAGC
	R	TTTTGTGAACGTGGACTGGA
TGFBRIII	F	CAGTCCACATCCACCACAAG
	R	CACAGAACCCTCAGACACCA

Appendix Table S5 : detailed n and pvalues

Figure 1

Figure 1C	n=7 per group			
DAOY	non group3 vs HDMB03	non group3 vs D458	non group3 vs MED1603	non group3 vs D283
Psmad2/Actin	0,0135	< 0,0001	< 0,0001	0,0002

D283 vs MED1603	D283 vs D458	D283 vs HDMB03	D283 vs DAOY	D283 vs ONS76	D283 vs UW
0,0006	0,5221	0,0169	0,0041	0,007	0,0041

MED1603 vs D458	MED1603 vs HDMB03	MED1603 vs DAOY	MED1603 vs ONS	MED1603 vs UW
0,0006	0,0006	0,0006	0,0006	0,0006

D458 vs HDMB03	D458 vs DAOY	D458 vs ONS76	D458 vs UW
0,0006	0,0006	0,0012	0,0023

HDMB03 vs DAOY	HDMB03 vs ONS76	HDMB03 vs UW
0,1801	0,0169	0,0169

Figure 1D	non group3 vs MED1603	non group3 vs D283	non group3 vs D458	non group3 vs HDMB03
INHBB expression	0,0016	0,0016	0,0917	> 0,9999
	non group3 vs MED1603	non group3 vs D283	non group3 vs D458	non group3 vs HDMB03
TGFB3 expression	0,029	0,7443	0,2817	0,7483

Figure 2

Figure 2A			
DAOY	TGFb	ActB	n=5
Psmad2/Actin	0,0079	0,6825	
ONS	TGFb	ActB	n=4
Psmad2/Actin	0,0286	> 0,9999	
UW	TGFb	ActB	n=4
Psmad2/Actin	0,0286	0,0286	
HDMB03	TGFb	ActB	n=6
Psmad2/Actin	0,0119	0,0022	
D458	TGFb	ActB	n=5
Psmad2/Actin	0,1	0,0079	
1603MED	TGFb	ActB	n=3
Psmad2/Actin	0,7	0,7	
D283	TGFb	ActB	n=4
Psmad2/Actin	0,1	0,0286	

Fig 2B		
1603MED	Ab	n=4
Psmad2/Actin	0,000382311	
	FLST	n=4
	2,2781E-05	

Fig 2C		
1603MED		n=3
Psmad2/Actin	NCM vs. NCM+ab	0,35
Psmad2/Actin	HD-CM vs. HD-CM+ab	0,2
Psmad2/Actin	1603-CM vs NCM	0,05
Psmad2/Actin	1603-CM vs HD-CM	0,05
Psmad2/Actin	1603-CM vs 1603-CM+Ab	0,05

Fig 2D	n=3
1603MED	siCTRL vs. siINHBB
expression	0,0013

Fig 2E	n=8
1603MED	siCTRL vs. siINHBB
Psmad2/Actin	5,50897E-06

Fig 2F	n=3
1603MED	siCTRL vs. siINHBB
growth curve	0,03

Figure 3

Figure 3A		
D458	ActB	n=3
Psmad2/Actin	0,000575283	

Figure 3B		
D458	ActB	n=3
growth curve	< 0,0001	

Figure 3C		
D458	ActB	n=3
G0-G1	0,0095	
S	0,0065	
G2+M	0,0732	
Figure 3D		
D458	ActB	n=3
cleaved-caspase3	0,0155	

Figure 3E		
D283	ActB	n=4
Psmad2/Actin	0,000294251	

Figure 3F		
D283	ActB	n=4
growth curve	< 0,0001	

Figure 3G		
D283	ActB	n=3
G0-G1	0,0023	
S	0,0027	
G2+M	0,7211	

Figure 3H		
D283	ActB	n=3
cleaved-caspase3	0,1453	

Figure 4

Figure 4A			
1603MED	LY	SB	n=3
Psmad2/Actin 24h	9,11709E-06	4,56647E-06	

Figure 4B			
1603MED	LY	SB	n=3
growth curve	0,0023	0,0014	

Figure 4C			
1603MED	LY	SB	n=3
G0-G1	0,0277	0,0302291	
S	0,0178	0,0259227	
G2+M	0,9898	0,863702	

Figure 4D			
1603MED	LY	SB	
cleaved-caspase3	0,0293	0,0262	n=4

Figure 4E			
D283	LY	SB	n=3
Psmad2/Actin	1,75241E-05	8,51551E-06	

Figure 4F			
D283	LY	SB	n=3
growth curve	0,0002	< 0,0001	

Figure 4G			
D283	LY	SB	n=3
G0-G1	0,2439	0,0960529	
S	0,0046	0,00437473	
G2+M	0,7114	0,828739	

Figure 4H			
D283	LY	SB	n=3
cleaved-caspase3	0,0084	0,0048	

Figure 5

Figure 5D	
PMEPA1/Actin	
Group WNT vs. group SHH	0,893
Group WNT vs. group 3	0,0014
Group WNT vs. group 4	0,1395
Group SHH vs. group 3	< 0,0001
Group SHH vs. group 4	0,0007
Group 3 vs. group 4	0,0349

group WNT	group SHH	group 3	group 4
n=7	n=12	n=10	n=9

Figure 5G		
1603MED	siCTRL vs. siPMEPA1pool	
PMEPA1/Actine	0,0286	n=4

Figure 5H		
1603MED	siCTRL vs. siPMEPA1pool	n=3
growth curve	0,046	

Figure 5I		
D283	siCTRL vs. siPMEPA1pool	
PMEPA1/Actine	0,0006	n=7

Figure 5J		
D283	siCTRL vs. siPMEPA1pool	
growth curve	0,0002	n=3

Figure 6

Figure 6C			
PDX3	TGFb	ActB	n=4
Psmad2/Actin	0,3143	0,0286	

PDX4	TGFb	ActB	n=3
Psmad2/Actin	0,1	0,7	

PDX7	TGFb	ActB	n=4
Psmad2/Actin	> 0,9999	0,0286	

Figure 6D		
PDX4	n=3	
Psmad2/Actin	NCM vs. NCM+ab	0,25
Psmad2/Actin	HD-CM vs. HD-CM+ab	0,25
Psmad2/Actin	PDX4-CM vs NCM	0,05
Psmad2/Actin	PDX4-CM vs HD-CM	0,05
Psmad2/Actin	PDX4-CM vs PDX4-CM+Ab	0,05

Figure 7

Figure 7A

Ctrl vs Galu: Pvalue = 0,0001***
Ctrl vs Galu+Cis: Pvalue = 0,0096**
Ctrl vs Cis: Pvalue =0,1021 ns
Galu vs Galu+Cis: Pvalue = 0,3441 ns
Galu vs Cis: Pvalue = 0,0022 **
Galu+Cis vs Cis: Pvalue = 0,08 ns

Figure 7B			
	CTRL vs. Cis	CTRL vs. Galu	CTRL vs. Galu+Cis
tumour size	0,0431	0,0574	0,0347
CTRL n=10	Cis n=10	Galu n=6	Galu + Cis n=7

staining quantifications	CTRL vs. Cis	CTRL vs. Galu	CTRL vs. Galu+Cis
Psmad2/Total area	0,5962	0,001	0,0085

Figure EV1

Fig EV1A	
ACTIN NORMALISATION	
Group WNT vs. group SHH	0,0098
Group WNT vs. group 3	0,0136
Group WNT vs. group 4	0,4575
Group SHH vs. group 3	< 0,0001
Group SHH vs. group 4	0,002
Group 3 vs. group 4	0,0522
SMAD NORMALISATION	
Group WNT vs. group SHH	0,0098
Group WNT vs. group 3	0,4028
Group WNT vs. group 4	0,2463
Group SHH vs. group 3	< 0,0001
Group SHH vs. group 4	0,0003
Group 3 vs. group 4	0,7655
WNT	n=7
SHH	n=12
Gp3	n=10
Gp4	n=9

Figure EV2

Fig EV2A				
1603MED	siCTRL vs. siINHBB	siCTRL vs. siINHBB+ActB	siINHBB vs siINHBB+ActB	
Psmad2/Actin	5,50897E-06	0,000014683	7,62238E-06	n=8

Fig EV2B				
1603MED	SiCTRL - SiINHBB	SiINHBB vs. siINHBB+Ab	SiCTRL vs. SiINHBB+Ab	n=3
	0,0142	0,0195	0,294	

Figure EV3

Fig EV3A				
1603MED	siCTRL vs. siINHBB	siCTRL vs. siINHBB+ActB	siINHBB vs siINHBB+ActB	
Psmad2/Actin	5,50897E-06	0,000014683	7,62238E-06	n=8
PMEPA1/Actine	5,23226E-08	0,00624415	0,000258717	n=7
MYC/Actine	0,00118098	0,916968	0,103082	n=4
OTX2/Actine	0,914512	0,36956	0,426926	n=4

Fig EV3B			
D283	siCTRL vs. siPMEPA1#1	siCTRL vs. siPMEPA1p#2	
PMEPA1/Actine	0,0006	0,0006	n=7

Fig EV3C			
D283	siCTRL vs. siPMEPA1#1	siCTRL vs. siPMEPA1p#2	n=7
confluency	0,0003	0,0003	

Fig EV3D			
1603MED	siCTRL vs. siPMEPA1#1	siCTRL vs. siPMEPA1p#2	
PMEPA1/Actine	0,0286	0,0286	n=4

Fig EV3E			
1603MED	siCTRL vs. siPMEPA1#1	siCTRL vs. siPMEPA1p#2	n=3
cell number	0,0079	0,0357	

Figure EV4

Fig EV4A		
1603MED	Galu	n=3
	0,0027	
D283	Galu	n=3
	0,0013	

Fig EV4C		
1603MED	GALU	n=4
Psmad2/Actin	0,000364399	
PMEPA1/Actine	0,000483894	
MYC/Actine	3,45566E-07	
OTX2/Actine	0,617171	
D283	GALU	n=4
Psmad2/Actin	8,40701E-07	
PMEPA1/Actine	0,00578699	
MYC/Actine	2,00173E-06	
OTX2/Actine	0,596591	
PDX3	GALU	n=3
Psmad2/Actin	2,16985E-10	
PMEPA1/Actine	1,31706E-07	
MYC/Actine	0,0110236	
OTX2/Actine	0,0024861	
PDX4	GALU	
Psmad2/Actin	1,4963E-06	
PMEPA1/Actine	1,05738E-07	
MYC/Actine	0,0702494	
OTX2/Actine	0,3786	
PDX7	GALU	n=3
Psmad2/Actin	2,40096E-12	
PMEPA1/Actine	0,000110627	
MYC/Actine	0,00016205	
OTX2/Actine	0,0090427	

Fig EV4E			
staining quantifications Psmad2/Total area	CTRL vs. Cis	CTRL vs. Galu	CTRL vs. Galu+Cis
Ki67	0,9134	0,5	0,4465
cc3	0,9134	0,0899	0,1304
CTRL n=12	Cis n=10	Galu n=6	Galu + Cis n=7

Appendix Figure S1

Appendix Figure S1A	CTRL vs. ActB	CTRL vs. ActB+Ab-aActB	ActB vs. ActB+Ab-aActB	n=4
Psmad2/Actin	0,0048	0,0048	0,0286	

	CTRL vs. ActB	ActB vs. ActB+LY	ActB vs. ActB+SB	ActB vs. ActB+Galu	n=4
	0,0079	0,0286	0,0286	0,0286	

Appendix Figure S1B	CTRL vs. TGFb	CTRL vs. TGFb+Ab-aActB	TGFb vs. TGFb+Ab-aActB	n=4
Psmad2/Actin	0,0286	0,0286	0,8286	

	CTRL vs. TGFb	TGFb vs. TGFb+LY	TGFb vs. TGFb+SB	TGFb vs. TGFb+Galu	n=3
	0,05	0,05	0,05	0,05	

Appendix Figure S2

Appendix Figure S2A		
HDMB03	ActB	n=5
Psmad2/Actin	0,0079	

Appendix Figure S2B		
HDMB03	ActB	
Confluency	0,468	n=4

Appendix Figure S3

Appendix Figure S3C			
1603MED	LY	SB	n=3
PMEPA1/Actine	0,000255484	2,11743E-05	
MYC/Actine	0,00096581	0,00378772	
OTX2/Actine	0,476047	0,52631	

D283	LY	SB	
PMEPA1/Actine	2,32921E-06	3,51897E-06	n=4
MYC/Actine	0,00360325	0,0246365	
OTX2/Actine	0,293633	0,623413	

D283	ActB	n=3
PMEPA1/Actine	0,00029355	
MYC/Actine	0,343751	
OTX2/Actine	0,0132184	

D458	ActB	n=4
PMEPA1/Actine	0,00368346	
MYC/Actine	0,0639103	
OTX2/Actine	0,000762444	

Appendix Figure S3D			
1603MED	LY	SB	n=3
Psmad2/Actin 24h	9,11709E-06	4,56647E-06	
PMEPA1/Actine	0,00500923	0,0079822	
MYC/Actine	0,00283955	0,0152516	
OTX2/Actine	0,409986	0,220658	

1603MED	Ab	n=4	FLST	n=4
Psmad2/Actin 24h	0,000382311		2,2781E-05	
PMEPA1/Actine	8,11523E-06		0,00271064	
MYC/Actine	0,00831629		0,0958693	
OTX2/Actine	0,876512		0,386375	

D283	LY	SB	n=3
Psmad2/Actin 24h	1,75241E-05	8,51551E-06	
PMEPA1/Actine	0,00219095	0,0285833	
MYC/Actine	0,00194504	0,0413334	
OTX2/Actine	0,480343	0,238429	

D283	ActB	n=4
Psmad2/Actin 24h	0,000294251	
PMEPA1/Actine	0,000582275	
MYC/Actine	0,001534	
OTX2/Actine	0,0359888	

D458	ActB	n=3
Psmad2/Actin 24h	0,000575283	
PMEPA1/Actine	0,0337263	
MYC/Actine	0,119118	
OTX2/Actine	0,864227	

Appendix Figure S4

Appendix Figure S4B			
PDX3	LY	SB	n=7
PMEPA1/Actine	7,58483E-10	8,45776E-09	
MYC/Actine	0,447207	0,462925	
OTX2/Actine	0,196957	0,00447424	

PDX4	LY	SB	n=5
PMEPA1/Actine	5,32316E-08	5,16547E-09	
MYC/Actine	0,0110324	0,145839	
OTX2/Actine	0,73397	0,786599	

PDX7	LY	SB	n=4
PMEPA1/Actine	0,00444009	0,00172518	
MYC/Actine	0,00264169	0,0748821	
OTX2/Actine	0,280295	0,54736	

PDX3	ActB	n=3
PMEPA1/Actine	0,0672152	
MYC/Actine	0,0753677	
OTX2/Actine	0,0546868	

PDX7	ActB	n=3
PMEPA1/Actine	0,216253	
MYC/Actine	0,113895	
OTX2/Actine	0,270416	

Appendix Figure S4C			
PDX3	LY	SB	n=6
Psmad2/Actin 24h	7,36256E-08	6,45964E-07	
PMEPA1/Actine	0,000123149	0,000202419	
MYC/Actine	0,309977	0,0286538	
OTX2/Actine	0,518319	0,375461	

PDX4	LY	SB	n=3

Psmad2/Actin 24h	0,000268245	0,000708827
PMEPA1/Actine	0,000224456	9,04785E-06
MYC/Actine	0,386431	0,914608
OTX2/Actine	0,946689	0,343322

PDX4	Ab	n=4
Psmad2/Actin 24h	0,00233629	
PMEPA1/Actine	0,00117035	
MYC/Actine	0,0184714	
OTX2/Actine	0,489486	

FLST	n=3
1,25386E-05	
1,5366E-05	
0,000015217	
0,00449743	

PDX7	LY	SB	n=4
Psmad2/Actin 24h	5,70068E-09	3,62086E-09	
PMEPA1/Actine	1,16054E-06	0,000352	
MYC/Actine	0,00158669	0,000403743	
OTX2/Actine	0,154355	0,17975	

PDX3	ActB	n=4
Psmad2/Actin 24h	0,149101	
PMEPA1/Actine	0,0346483	
MYC/Actine	0,000885645	
OTX2/Actine	0,136323	

PDX7	ActB	n=5
Psmad2/Actin 24h	0,00283622	
PMEPA1/Actine	7,45566E-05	
MYC/Actine	0,00258122	
OTX2/Actine	0,156133	

Appendix Figure S5

Appendix Figure S5 - Figure 1C

	D458 vs All	HDMBO3 vs All	MED1603 vs all	D283 vs all
Psmad2/Smad2	0,0011	0,2685	< 0,0001	0,6494

n=8 per cell line

Appendix Figure S5 - Figure 2A

DAOY	TGFb	ActB	n=5
Psmad2/Smad2	0,0079	0,6825	

ONS	TGFb	ActB	n=4
Psmad2/Smad2	0,0286	> 0,9999	

UW	TGFb	ActB	n=4
Psmad2/Smad2	0,0286	0,3143	

HDMB03	TGFb	ActB	n=6
Psmad2/Smad2	0,4405	0,0022	

D458	TGFb	ActB	n=5
Psmad2/Smad2	0,4643	0,0079	

1603MED	TGFb	ActB	n=3
Psmad2/Smad2	0,1	0,1	

D283	TGFb	ActB	n=4
Psmad2/Smad2	0,0286	0,0286	

Appendix Figure S5 - Figure 2B and S3D

1603MED	Ab	n=4
Psmad2/Smad2	0,000486899	
	FLST	n=4
Psmad2/Smad2	3,64454E-05	

Appendix Figure S5 - Figure 2C

1603MED	n=3	
Psmad2/Smad2	NCM vs. NCM+ab	0,3377
Psmad2/Smad2	HD-CM vs. HD-CM+ab	0,35
Psmad2/Smad2	1603-CM vs NCM	0,0119
Psmad2/Smad2	1603-CM vs HD-CM	0,0119
Psmad2/Smad2	1603-CM vs 1603-CM+Ab	0,05

Appendix Figure S5 - Figure EV2A

1603MED	siCTRL vs. siINHBB	siCTRL vs. siINHBB+ActB	siINHBB vs siINHBB+ActB	
Psmad2/Smad2	5,33998E-05	0,0069216	0,000588218	n=8

Appendix Figure S5 - Figure 3A

D458	ActB	n=3
Psmad2/Smad2	0,00151788	

Appendix Figure S5 - Figure 3E

D283	ActB	n=4
Psmad2/Smad2	0,00271302	

Appendix Figure S5 - Figure 4E and 4A

D283	LY	SB	n=3
Psmad2/Smad2	5,09217E-06	7,20888E-07	

MED1603	LY	SB	n=3
Psmad2/Smad2	2,21229E-06	2,6165E-06	

Appendix Figure S5 - Figure 6C

PDX3	TGFb	ActB	n=4
Psmad2/Smad2	0,1587	0,0159	

PDX4	TGFb	ActB	n=3
Psmad2/Smad2	0,7	0,7	

PDX7	TGFb	ActB	n=4
Psmad2/Smad2	0,3143	0,0286	

Appendix Figure S5 - Figure 6D

PDX4	n=3	
Psmad2/Smad2	NCM vs. NCM+ab	0,375
Psmad2/Smad2	HD-CM vs. HD-CM+ab	0,35
Psmad2/Smad2	PDX4-CM vs NCM	0,0119
Psmad2/Smad2	PDX4-CM vs HD-CM	0,0119
Psmad2/Smad2	PDX4-CM vs PDX4-CM+Ab	0,05

Appendix Figure S5 - Figure EV4C

1603MED	GALU	n=4
Psmad2/Smad2	3,45566E-07	

D283	GALU	n=4

Psmad2/Smad2	1,78302E-05

PDX3	GALU	n=6
Psmad2/Smad2	1,10815E-14	

PDX4	GALU	n=3
Psmad2/Smad2	1,72102E-05	

PDX7	GALU	n=3
Psmad2/Smad2	1,08588E-09	

Appendix Figure S5 - Appendix Figure S1A

	CTRL vs. ActB	CTRL vs. ActB+Ab-aActB	ActB vs. ActB+Ab-aActB	n=4
Psmad2/Smad2	0,0022	0,0476	0,0087	

	CTRL vs. ActB	ActB vs. ActB+LY	ActB vs. ActB+SB	ActB vs. ActB+Galu	n=4
Psmad2/Smad2	0,0286	0,0286	0,0286	0,0286	

Appendix Figure S5 - Appendix Figure S1B

	CTRL vs. TGFb	CTRL vs. TGFb+Ab-aActB	TGFb vs. TGFb+Ab-aActB	n=4
Psmad2/Smad2	0,0079	0,0079	0,8571	

	CTRL vs. TGFb	TGFb vs. TGFb+LY	TGFb vs. TGFb+SB	TGFb vs. TGFb+Galu	n=3
Psmad2/Smad2	0,0286	0,0286	0,0286	0,0286	

Appendix Figure S5 - Appendix Figure S2A

HDMB03	ActB	n=5
Psmad2/Smad2	0,0079	

Appendix Figure S5 - Appendix Figure S4C

PDX3	LY	SB	n=6
Psmad2/Smad2	1,34017E-08	5,04918E-06	

PDX4	LY	SB	n=3
Psmad2/Smad2	0,0010285	0,00183895	

PDX4	Ab	n=4	FLST	n=3
Psmad2/Smad2	0,00218201		2,36944E-07	

PDX7	LY	SB	n=4
Psmad2/Smad2	4,12389E-09	3,90988E-09	

PDX3	ActB	n=4
Psmad2/Smad2	0,136817	

PDX7	ActB	n=5
Psmad2/Smad2	0,000204885	

Appendix Supplemental Figure legends

<u>**Appendix Figure S1:**</u> **Pharmacological inhibitors and blocking antibody specificity.** (A-B) WB analysis performed in D283 group 3 MB cell line (A) and UW228 non group 3 MB cell line (B).The level of phosphorylated Smad2 (P-Smad2) and total Smad2 (Smad2) was assessed by Immunoblot in response to DMSO or LY364947 or SB431542 or Galunisertib or ActivinB blocking antibody (Ab α-ActB) after either ActivinB (ActB) or TGFβ stimulation for 1 hour. β-actin was used as a loading control. Right panel represents relative quantifications of the P-S2/ β-actin. The p-values were determined by unpaired t-test. *p < 0.05, **p < 0.01, ***p < 0.001. Bars represent the mean ± SD. Number of replicates is n≥3.

<u>**Appendix Figure S2:**</u> ActivinB stimulation does not promote growth in HDMB03 cells (A-B) HDM03 cell line was treated with PBS (vehicle, black) or with ActivinB (green). (A) Immunoblot of phosphorylated Smad2 (P-Smad2), total Smad2 and β-actin in response to ActivinB stimulation for 24 hours. Quantification of P-Smad2 (P-S2) to β-actin is shown on right panels. (B) Growth curve experiments showing cell proliferation upon ActivinB treatment. **p < 0.01. Bars represent the mean ± SD. Number of replicates is n≥3.

<u>**Appendix Figure S3:**</u> ***PMEPA1* is a target gene of Activin signaling.** (A) Scatter plot of *INHBB* and *PMEPA1* gene expression levels in the 4 individual MB groups. Note that panel, corresponding to group 3 (yellow) is identical to that shown in Figure EV4E, except for the colour code (B) Scatter plots of P-Smad2 and PMEPA1 protein levels in the 4 MB groups. (A-B) Colored dots represent each patient samples and colors represent the MB groups (WNT in blue, SHH in red, Group 3 in yellow and Group 4 in green). Spearman's rank correlation coefficient ρ and p-value are indicated on top. (C) RT-qPCR were performed on RNA extracted from 1603MED, D283 and D458 to compare expression levels of target genes upon inhibition and stimulation of TGFβ/Activin signaling by LY364947 or SB431542 inhibitors and ActivinB stimulation. Relative expression level to control is presented. (D) Quantification of the WB presented in Figure 5G. Bar graphs represent the relative quantifications of the indicated proteins relative to β-actin. Note that P-Smad2/b-actin quantification is identical to quantification in Figure 2B, 3A, 3E, 4A and 4E.The different treatments are indicated. The level in control conditions was set at 1. The p-values were determined by Spearman rank correlation test for A and B and unpaired t-test for C and D. Detailed statistics are presented in Appendix Table S3 for A. *p < 0.05, **p < 0.01 and ***p < 0.001. Bars represent the mean ± SD. Number of replicates is n≥3.

Appendix Figure S4: TGFβ/ActivinB signaling in group 3 MB PDXs. (A) Relative mRNA expression of TGFβ/ActivinB different mediators in G3 PDXs and cell lines. Expression in HDMB03 was set at 1. RT-qPCR were performed on RNA extracted from Group 3 MB cell lines and PDXs as indicated to compare the expression levels of mediators of the TGFβ/ActivinB pathway in the different PDXs in regards to cell lines: *INHBA*, *TGFB1*, *TGFB2*, *TGFB3*, *ACVR1B*, *ACVR1C*, *ACVR2A*, *ACVR2B, TGFBR1* and *TGFBR2*. (B) RT-qPCR were performed on RNA extracted from Group 3 MB cell lines and PDXs to compare relative expression of target genes upon inhibition or stimulation of TGFβ/Activin signaling by LY364947 and SB431542 inhibitors and ActivinB stimulation. Expression in control condition was set at 1. Detailed statistics are presented in Appendix Table S2. (C) Quantification of WB presented in Figure 6E. The relative level to β-actin is presented for the different proteins. The level in control conditions was set at 1. The p-values were determined by unpaired t-test. *p < 0.05, **p < 0.01 and ***p < 0.001. Bars represent the mean ± SD. Number of replicates is n≥3.

Appendix Figure S5: P-Smad2/Total Smad2 normalization

The P-Smad2/Total Smad2 normalization is provided for all relevant WB in the manuscript figures as indicated by the corresponding Figure panel number. Code colors as well as numbers below the plots are identical to those in the corresponding figure panels.

Appendix Supplemental Table legends

Appendix Table S1: Statistics related to Figure 1B, 5B-C and EV1B
Statistics related to transcriptomic analyses on dataset from "Data ref: Cavalli et al, 2017". (Cancer Cell. 2017; 31(6):737-754.e6. doi: 10.1016/j.ccell.2017.05.005.)

Appendix Table S2: Statistics related to Figure 1D, 6B and EV1D and Appendix Figure S4A
Statistics related to RT-qPCR experiments assessing expression levels of the TGFβ/ActivinB signaling pathway mediators performed in cell lines and PDX

Appendix Table S3: Statistics related to Figure 7D-E
Spearman correlation (ρ and p-values) related to the analyses performed on dataset from "Data ref: Cavalli et al, 2017" (Cancer Cell. 2017; 31(6):737-754.e6. doi: 10.1016/j.ccell.2017.05.005.) for INHBB, PMEPA1, MYC and NRL.

Appendix Table S4: Sequences of the oligonucleotides used in this study for RT-qPCR experiments

Appendix Table S5: Exact p-values and number of replicates for each experiment.
The exact p-values and number of replicates corresponding to the relevant figures and panels are provided.
