# Supplementary material for: An autocrine ActivinB mechanism drives TGFβ/Activin signaling in Group 3 medulloblastoma
Source: EMBO Mol Med. 2019 Jul 22;11(8):e9830. doi: 10.15252/emmm.201809830 (PMC6685082; doi:10.15252/emmm.201809830)
Supplement: Supplementary file 2 — Expanded View Figures PDF [file EMMM-11-e9830-s002.pdf]

Expanded View Figures

Figure EV1. TGFβ/ActivinB pathway is activated in Group 3 MB patients and cell lines.

A Boxplots represent the quantification of P-Smad2 normalized to β-actin levels on the left and total Smad2 on the right.
B Boxplots summarizing the expression of major actors of the TGFβ/Activin pathway in the different groups of MB (blue WNT, red SHH, yellow Group 3, and green Group 4) in the dataset from Cavalli *et al* (Data ref: Cavalli *et al*, 2017b).
C Boxplots summarizing the protein level of major actors of the TGFβ/Activin pathway detected by mass spectrometry in the different groups of MB (blue WNT, red SHH, yellow Group 3, and green Group 4) in the dataset from Archer *et al* (Data ref: Archer *et al*, 2018b).
D RT–qPCR was performed on RNA extracted from non-Group 3 (blue) and Group 3 (yellow) MB cell lines to compare expression levels of *INHBA*, *TGFB1*, *TGFB2*, *TGFBR1*, *TGFBR2*, *ACVR1B*, *ACVR1C*, *ACVR2A*, and *ACVR2B*. The expression level relative to that of found in HDMB03 is presented.

Data information: Boxplot center lines show data median; box limits indicate the 25th and 75th percentiles; lower and upper whiskers extend 1.5 times the interquartile range (IQR) from the 25th and 75th percentiles, respectively (A, B and C). Outliers are represented by individual points (B). The *P*-values were determined by unpaired *t*-test in panels (A and D), Wilcoxon rank-sum tests for panels (B and C). $^{*}P < 0.05$, $^{**}P < 0.01$, $^{***}P < 0.001$, and $^{****}P < 0.0001$. Detailed statistics are presented in Appendix Table S5 for A, Appendix Table S1 for panel (B), and Appendix Table S2 for panel (D). Bars represent the mean ± SD. Number of replicates is $n \geq 3$. The exact *P*-values and number of replicates are indicated in Appendix Table S5.

© 2019 The Authors

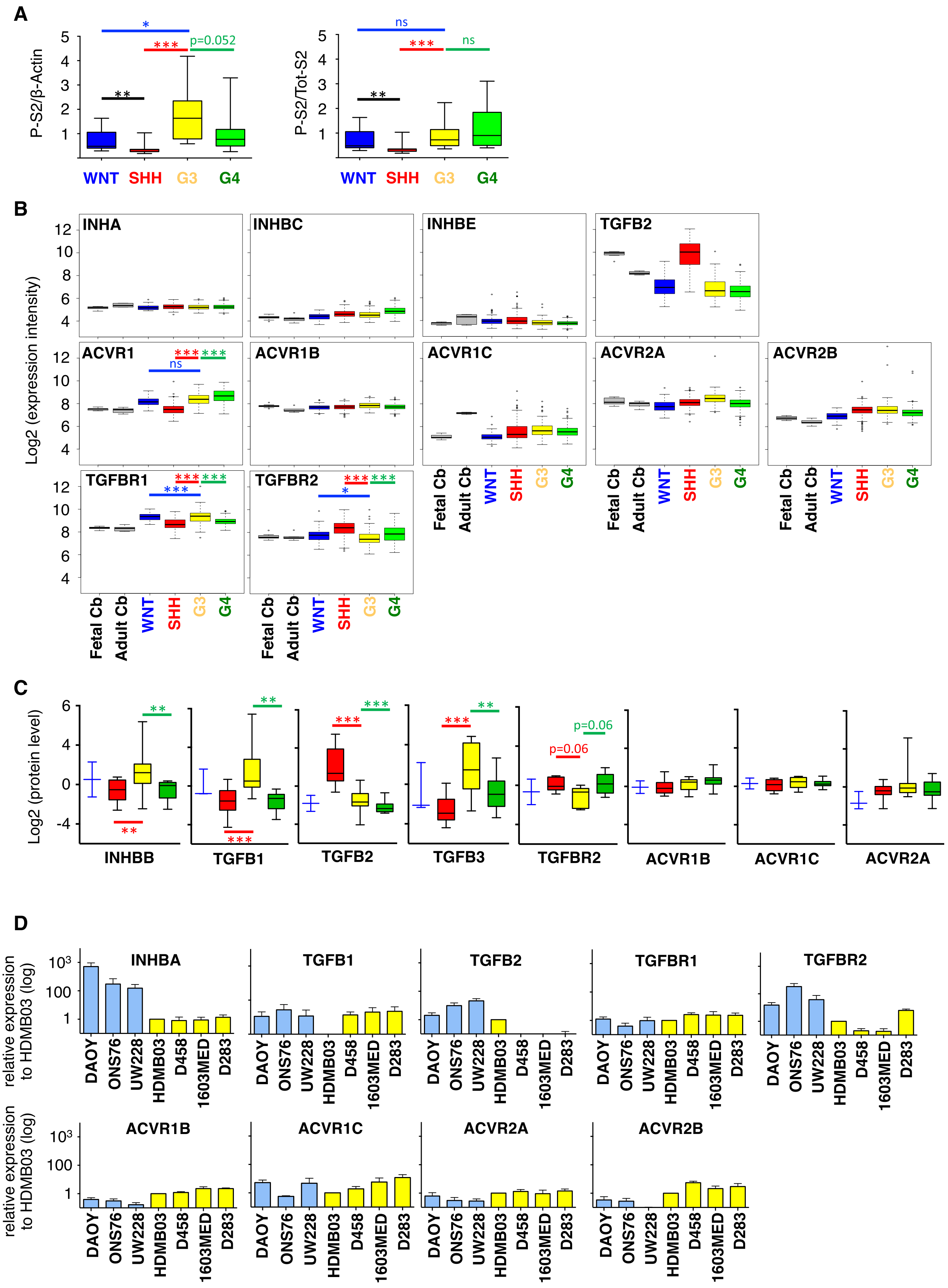
A
P-S2/β-Actin
P-S2/Tot-S2
p=0.052
ns
WNT SHH G3 G4
B
Log2 (expression intensity)
INHA
INHBC
INHBE
TGFB2
ACVR1
ACVR1B
ACVR1C
ACVR2A
ACVR2B
TGFBR1
TGFBR2
Fetal Cb Adult Cb WNT SHH G3 G4
C
Log2 (protein level)
p=0.06
INHBB
TGFB1
TGFB2
TGFB3
TGFBR2
ACVR1B
ACVR1C
ACVR2A
D
relative expression to HDMB03 (log)
INHBA
TGFB1
TGFB2
TGFBR1
TGFBR2
ACVR1B
ACVR1C
ACVR2A
ACVR2B
DAOY ONS76 UW228 HDMB03 D458 1603MED D283

Figure EV1.

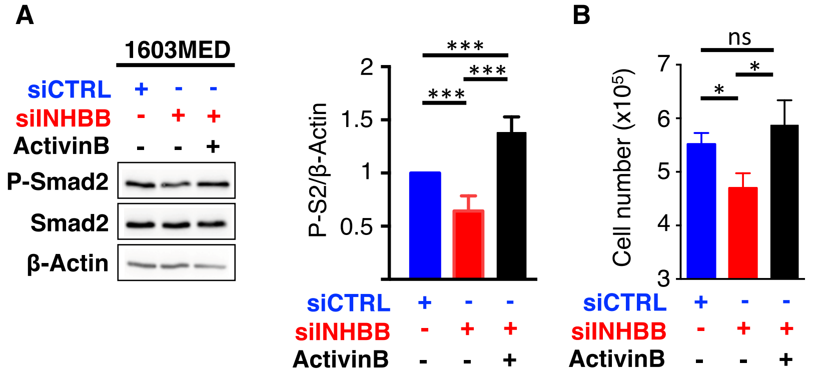

Figure EV2. An autocrine stimulation by ActivinB in the 1603MED cell line.

A, B The level of phosphorylated Smad2 (P-Smad2) and total Smad2 (Smad2) was assessed by immunoblot, and β-actin was used as a loading control in 1603MED cells transfected with the indicated control siRNA (siCTRL) or targeting INHBB (siINHBB). Rescue experiment was performed by adding exogenous ActivinB. Lysates were prepared 48 h after transfection. Right panel represents relative quantifications of the P-S2/β-actin ratio. Note that P-Smad2/β-actin quantification is identical to that of in Fig 2D. P-Smad2 to total Smad normalization is provided on Appendix Fig S5. (B) Number of viable 1603MED cells 2 days after transfection with either siCTRL (blue) or siINHBB (red). Rescue was assessed upon ActivinB stimulation (black). The *P*-values were determined by unpaired *t*-test. $^{*}P < 0.05$, $^{***}P < 0.001$. Bars represent the mean ± SD. Number of replicates is $n \geq 3$. The exact *P*-values and number of replicates are indicated in Appendix Table S5.

Source data are available online for this figure.

 © 2019 The Authors

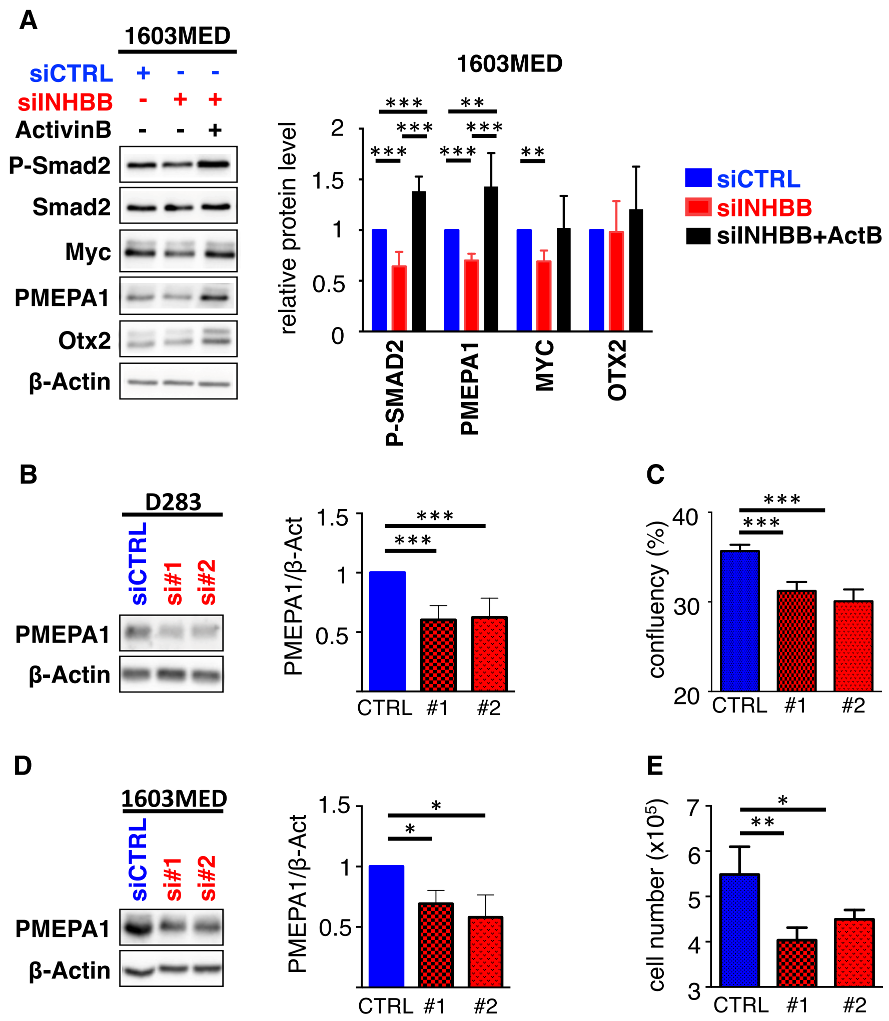

Figure EV3. *PMEPA1* is a target gene involved in the response to Activin signaling.

A Immunoblots of phosphorylated Smad2 (P-Smad2), total Smad2, MYC, PMEPA1, OTX2, and β-actin were performed on cell extracts from 1603MED transfected with control siRNA control (siCTRL, blue) or targeting INHBB (siINHBB, red). 1603MED cells transfected with siINHBB were also treated with ActivinB to investigate a potential rescue (black). Lysates were prepared 48 h after transfection. Right panel represents WB quantification. The relative level to β-actin is presented for the different proteins. Note that P-Smad2/β-actin quantification is identical to that of in Figs 2D and EV2A. The level in control conditions (siCTRL) was set at 1.

B–E D283 (B and C) and 1603MED (D and E) cell lines were transfected with siCTRL (blue) or individual siRNAs targeting PMEPA1 (siPMEPA1#1 or siPMEPA1#2 red). D283 (B) and 1603MED (D) lysates were prepared 48 h after transfection. Right panel represents WB quantifications performed as above. (C and E) Viable D283 (C) and 1603MED (E) cells measured 2 days after transfection with either siCTRL (blue) or siPMEPA1#1 and #2 (red).

Data information: The *P*-values were determined by unpaired *t*-test. *$P < 0.05$, **$P < 0.01$, and ***$P < 0.001$. Bars represent the mean ± SD. Number of replicates is $n \geq 3$. The exact *P*-values and number of replicates are indicated in Appendix Table S5.

Figure EV4. ActivinB signaling is a potential target for patients of group 3 MB.

A Growth curve experiments showing cell proliferation upon TGFβ/Activin signaling inhibition by Galunisertib (LY2157299) in 1603MED (top panel) and D283 (bottom panel) cell lines.

B Immunoblots of phosphorylated Smad2 (P-Smad2), total Smad2, MYC, PMEPA1, OTX2, and β-actin were performed on extracts from 1603MED and D283 cell lines and PDX3, PDX4, and PDX7 cell cultures treated with Galunisertib for 24 h.

C WB quantifications. The relative level to β-actin is presented for the different proteins. P-Smad2 to total Smad normalization is provided on Appendix Fig S5. The level in control conditions was set at 1.

D Ki67 and cleaved caspase-3 staining by IHC of 3 representative tumors per group of mice (Vehicle, Cisplatin, Galunisertib, and Galunisertib + Cisplatin). Scale bars indicate 100 μm.

E Boxplots represent quantification of Ki67 and cleaved caspase-3 (Cc3) staining on tumor (IHC).

Data information: Boxplot center lines show data median; box limits indicate the 25th and 75th percentiles; lower and upper whiskers extend 1.5 times the interquartile range (IQR) from the 25th and 75th percentiles, respectively. Outliers are omitted. The *P*-values were determined by two-way ANOVA in (A), unpaired *t*-test in (C) and Mann–Whitney test (E). *$P < 0.05$, **$P < 0.01$, and ***$P < 0.001$. Bars represent the mean ± SD. Number of replicates is $n \geq 3$. The exact *P*-values and number of replicates are indicated in Appendix Table S5.
Source data are available online for this figure.

© 2019 The Authors

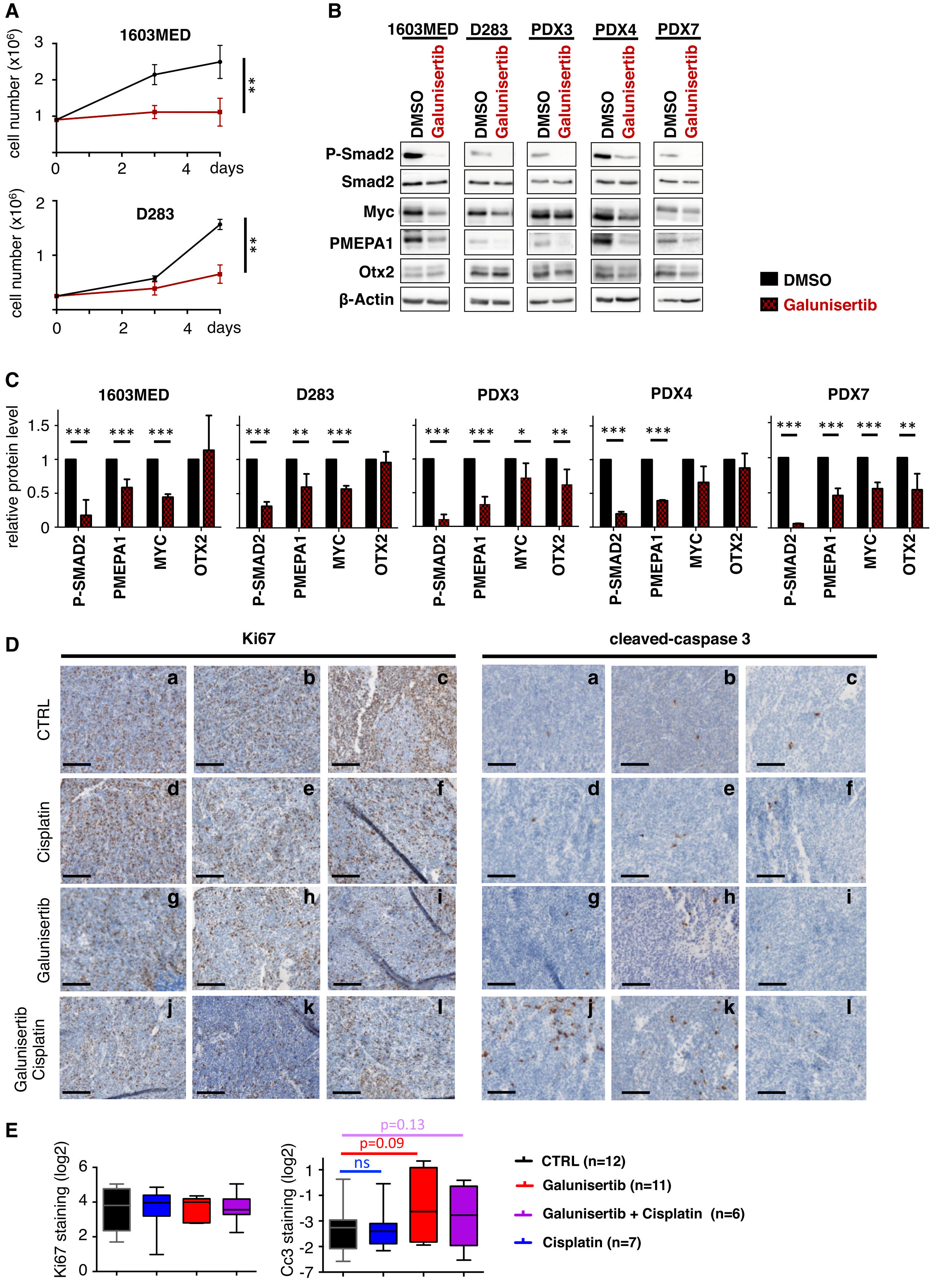

Figure EV4.

 © 2019 The Authors
