# Supplementary figures and images for: An autocrine ActivinB mechanism drives TGFβ/Activin signaling in Group 3 medulloblastoma
Source: EMBO Mol Med. 2019 Jul 22;11(8):e9830. doi: 10.15252/emmm.201809830 (PMC6685082; doi:10.15252/emmm.201809830)

Figure EV2

## silNHBB rescue – WB57

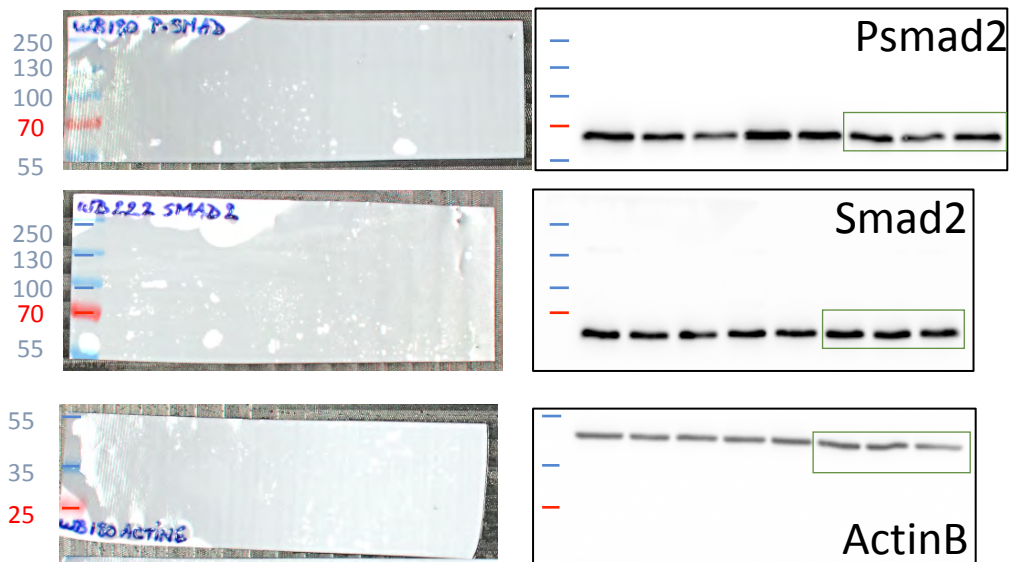

Supplement: Supplementary file 3 — Source Data for Expanded View and Appendix [file EMMM-11-e9830-s009.zip › EMM_2018_09830_V3_EV_Source_Data/EMM_2018_09830_V3_MorabitoR3_Source_data_Uncropped_WB_EV2.pdf]

Figure EV4

## 283 1603 GALU – WB34

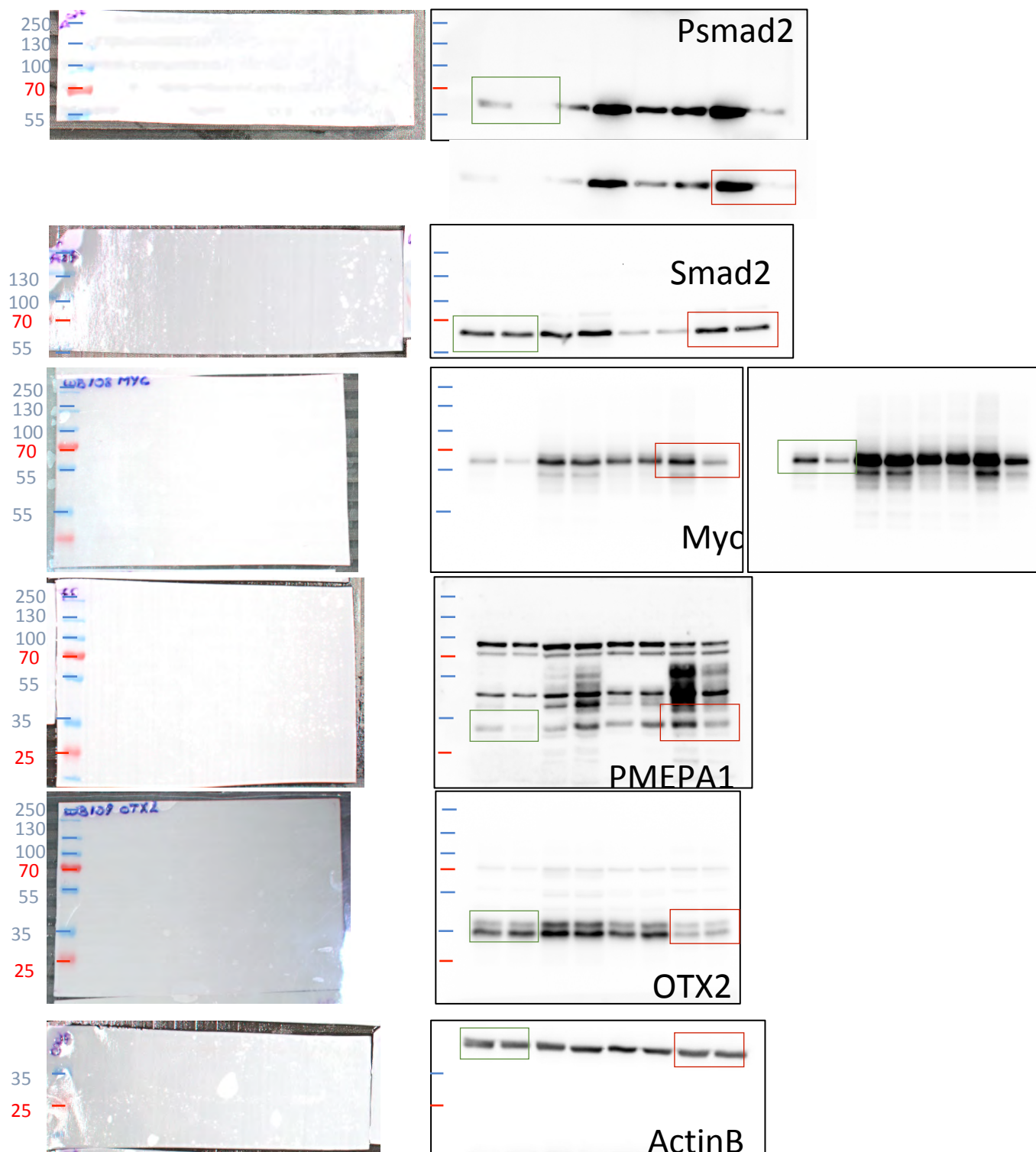

pdx3 GALU – WB54

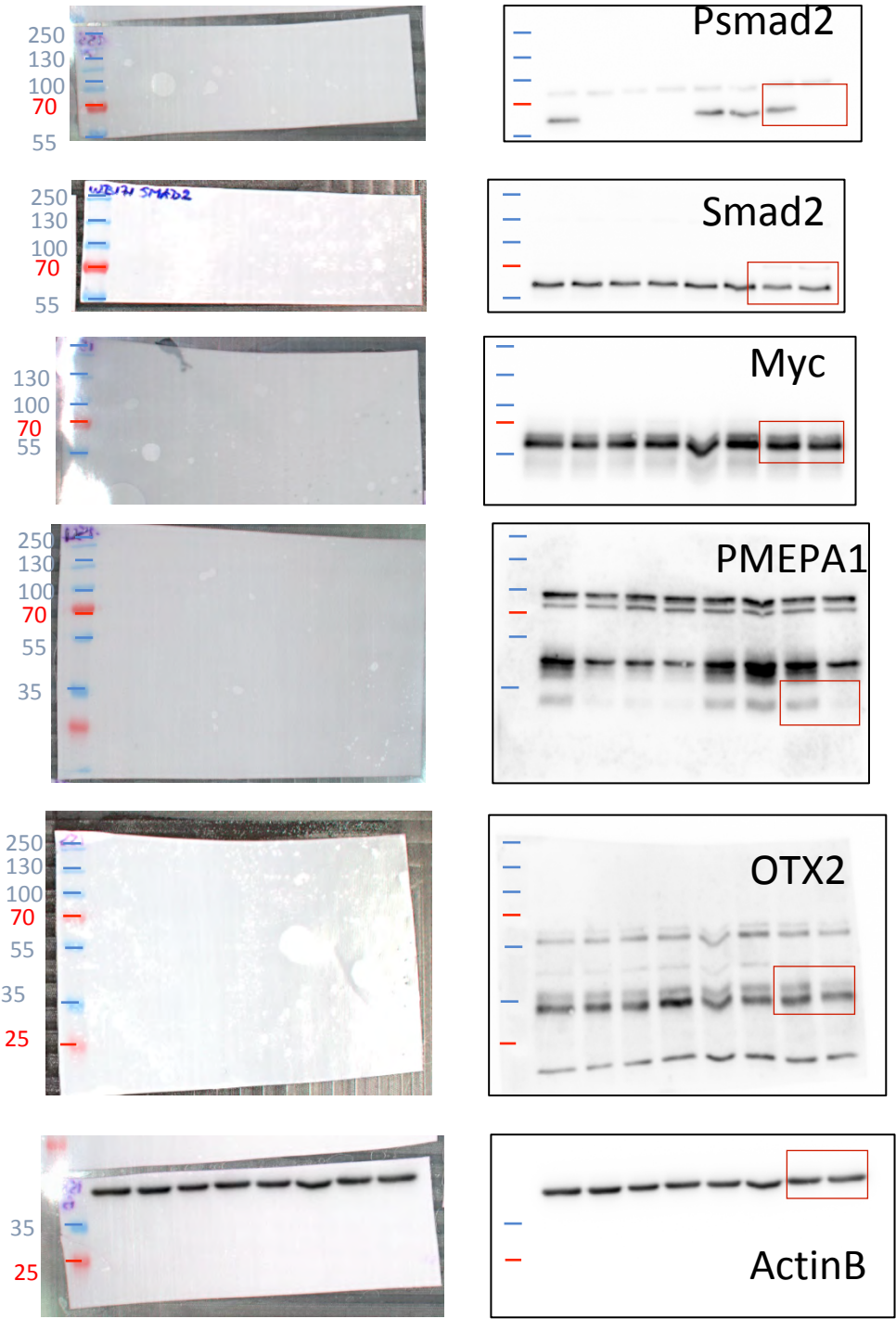

## pdx4 GALU – WB63

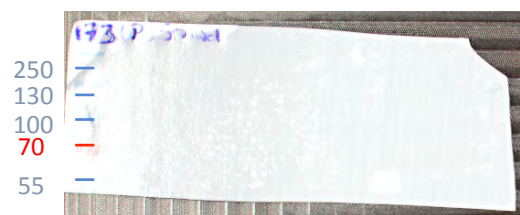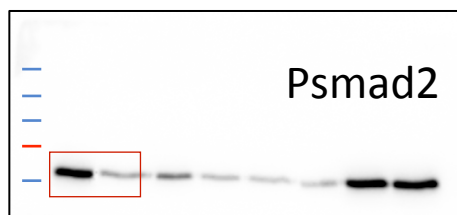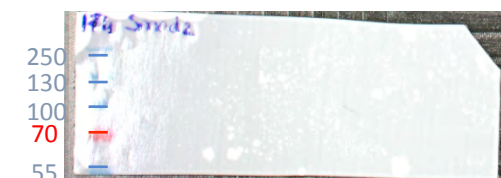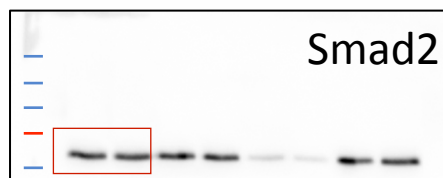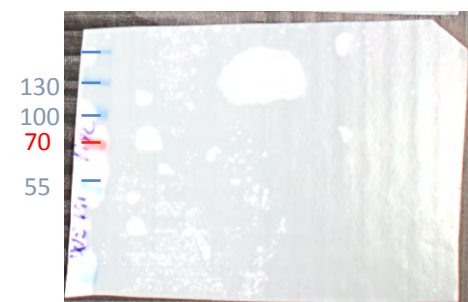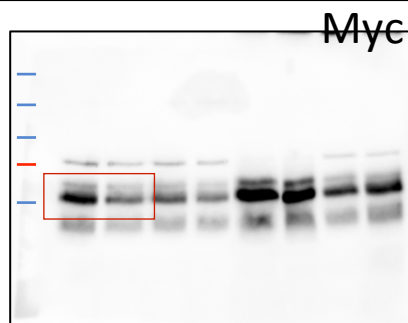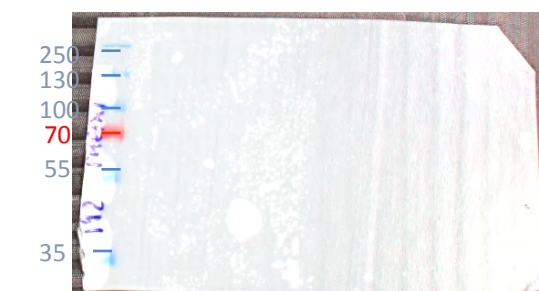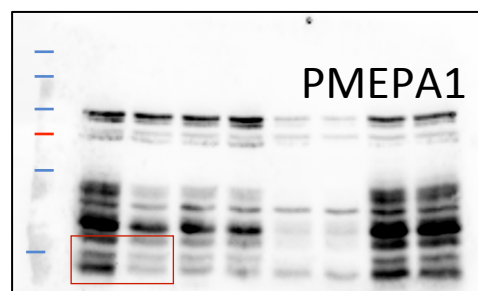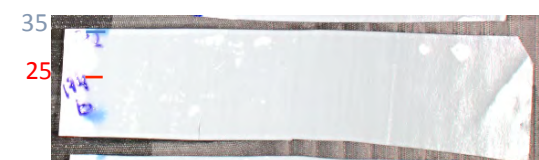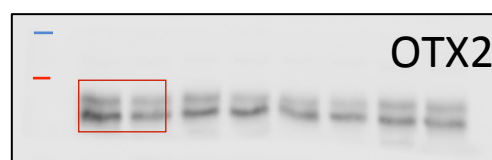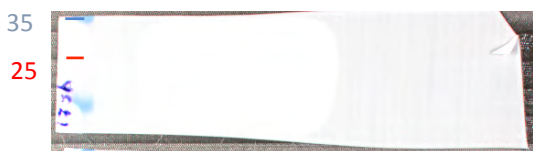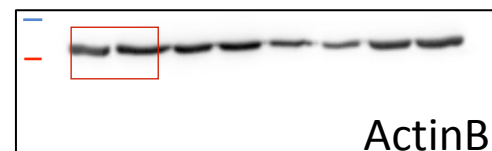

pdx7 GALU – WB40

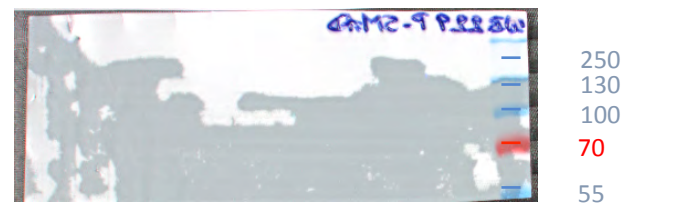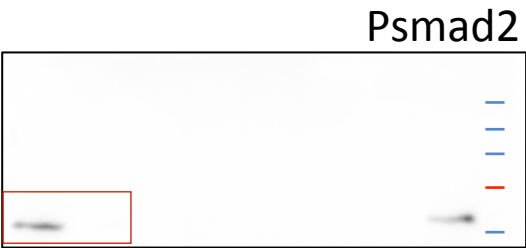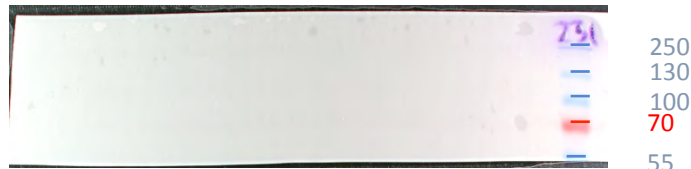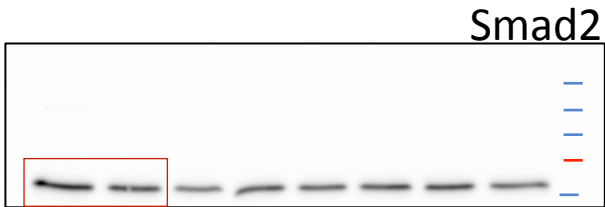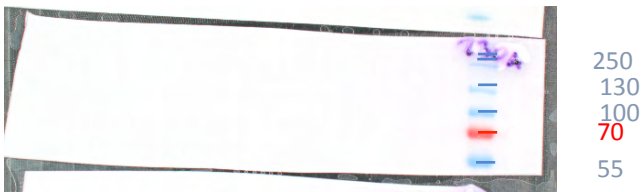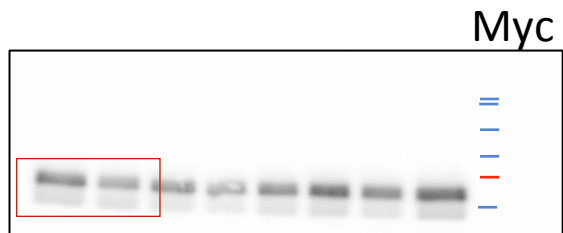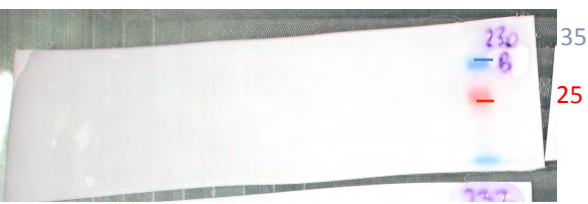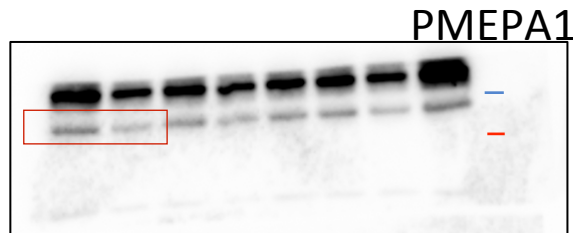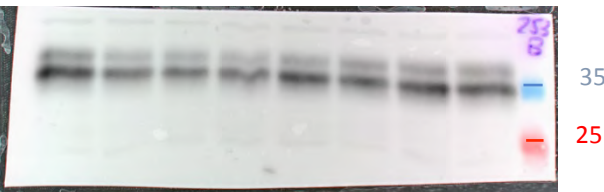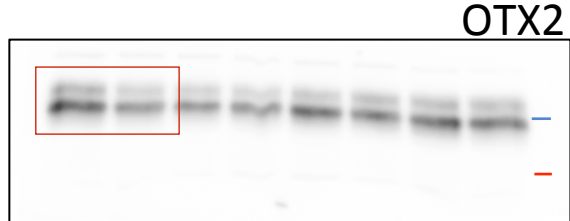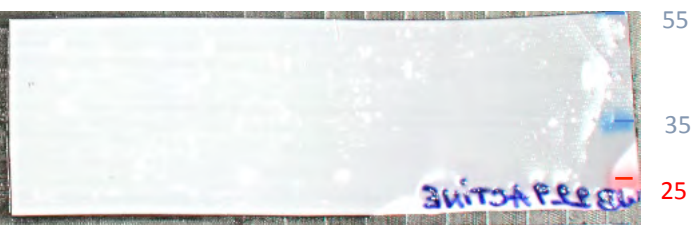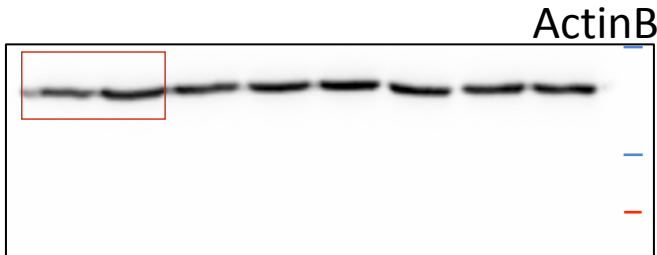

Supplement: Supplementary file 3 — Source Data for Expanded View and Appendix [file EMMM-11-e9830-s009.zip › EMM_2018_09830_V3_EV_Source_Data/EMM_2018_09830_V3_MorabitoR3_Source_data_Uncropped_WB_EV4.pdf]

# Figure S1

Ab specificity

# Figure S1A

**D283**

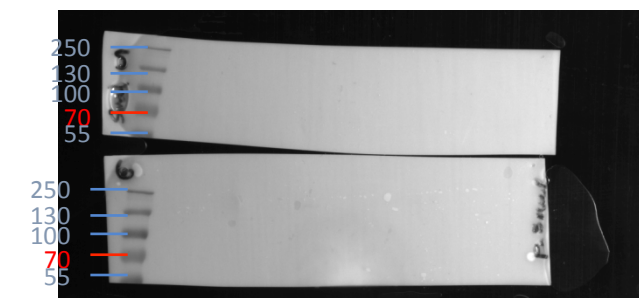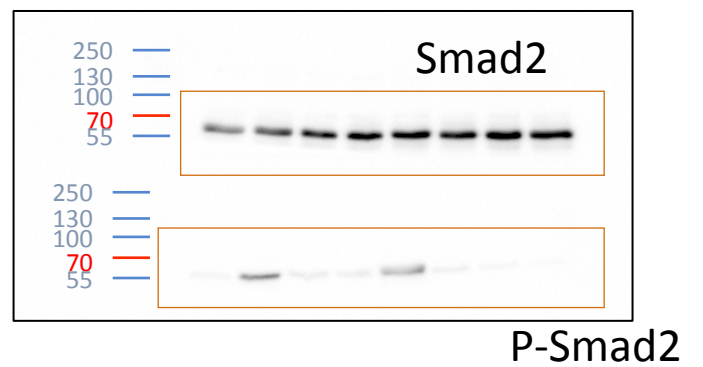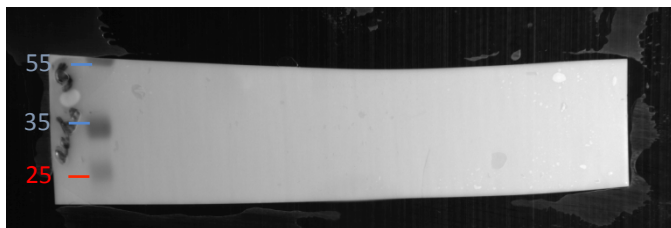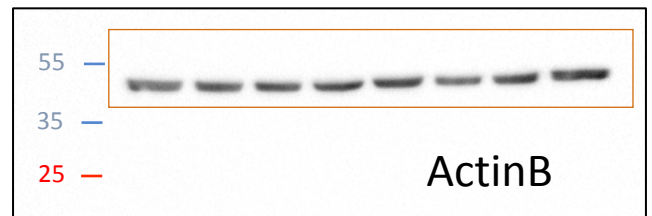

UW AB SPE MB 91. ET 269

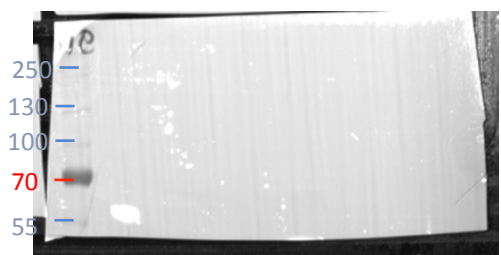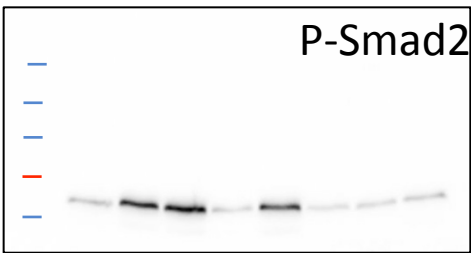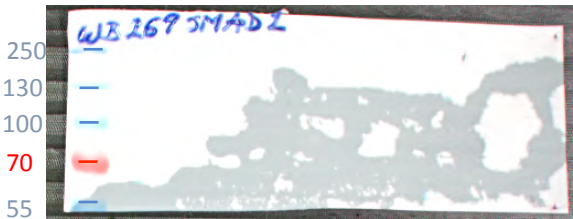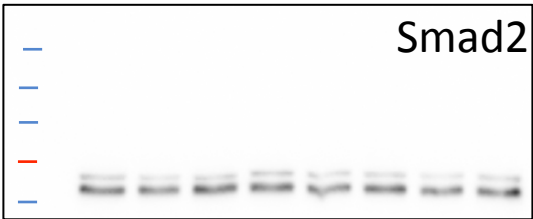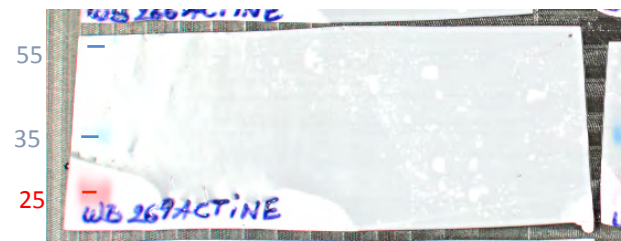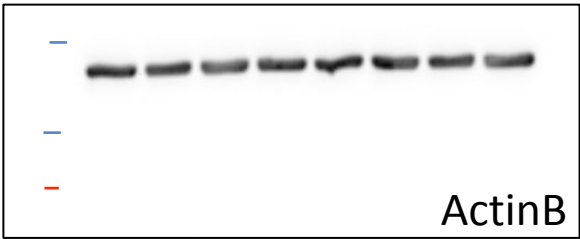

Supplement: Supplementary file 3 — Source Data for Expanded View and Appendix [file EMMM-11-e9830-s009.zip › EMM_2018_09830_V3_EV_Source_Data/EMM_2018_09830_V3_MorabitoR3_Source_data_Uncropped_WB_FIGS1.pdf]

# Figure S2

D458 D283 P/A

## HDMB03 PA WB29

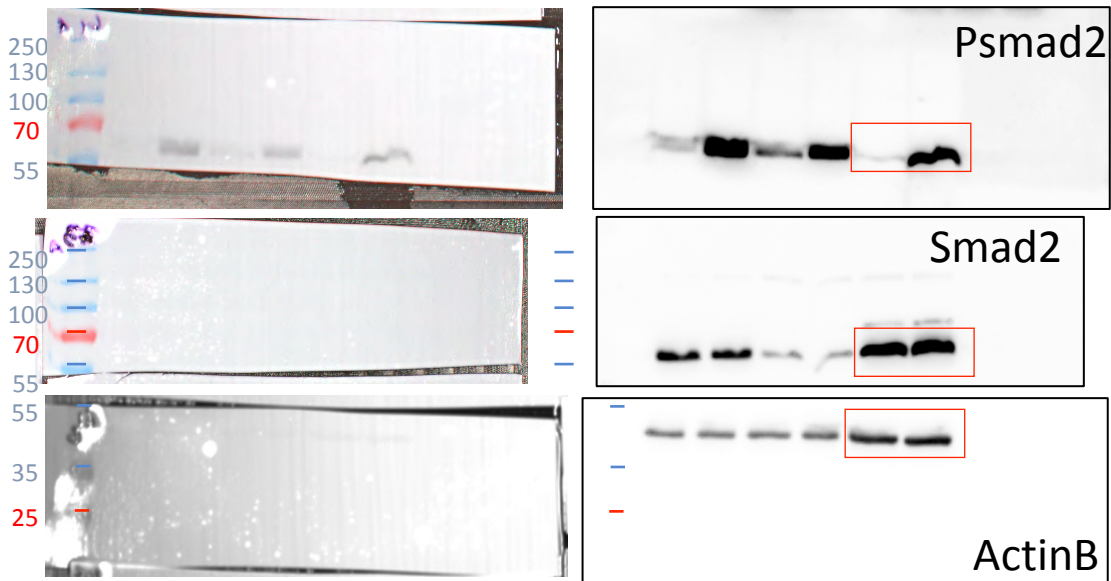

Supplement: Supplementary file 3 — Source Data for Expanded View and Appendix [file EMMM-11-e9830-s009.zip › EMM_2018_09830_V3_EV_Source_Data/EMM_2018_09830_V3_MorabitoR3_Source_data_Uncropped_WB_FIGS2.pdf]

# Figure 3

D458 D283 P/A

## D283

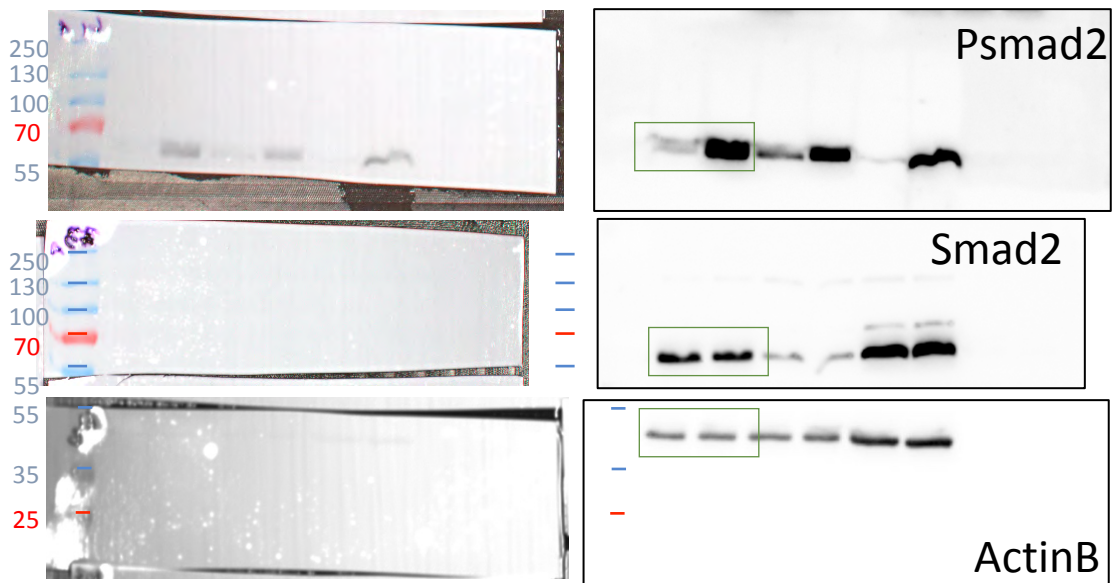

## D458 PA WB SAB\_ PSMAD

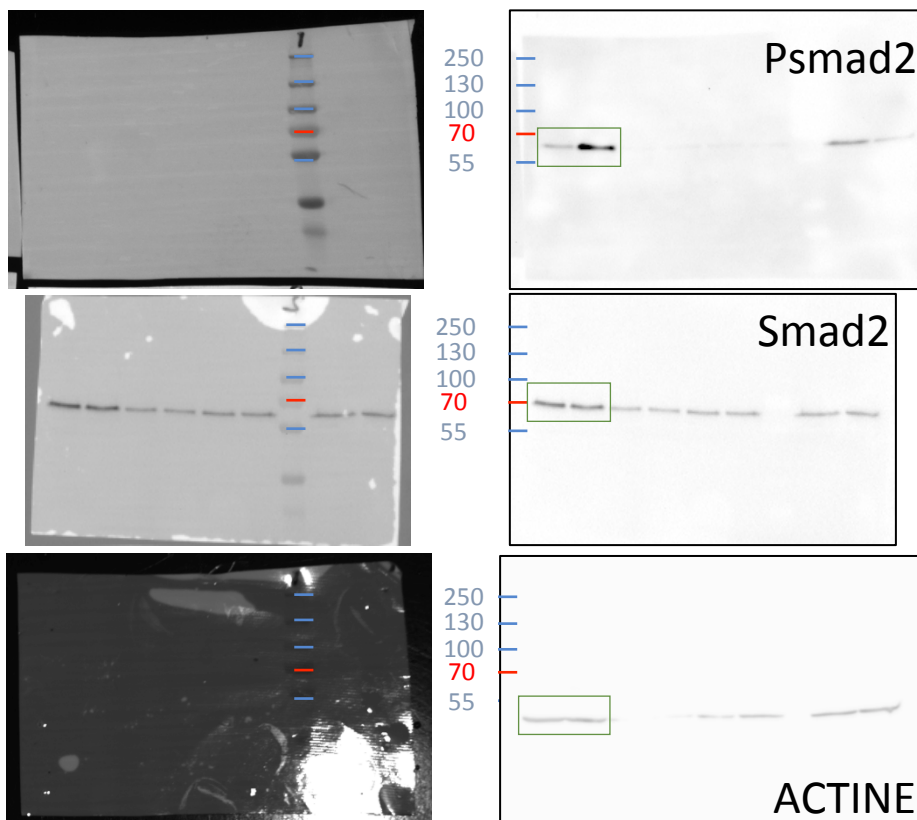

Supplement: Supplementary file 7 — Source Data for Figure 3 [file EMMM-11-e9830-s005.pdf]

# Figure 4

1603MEd and S283 D/L/S

## 1603MED D L S \_ PSMAD

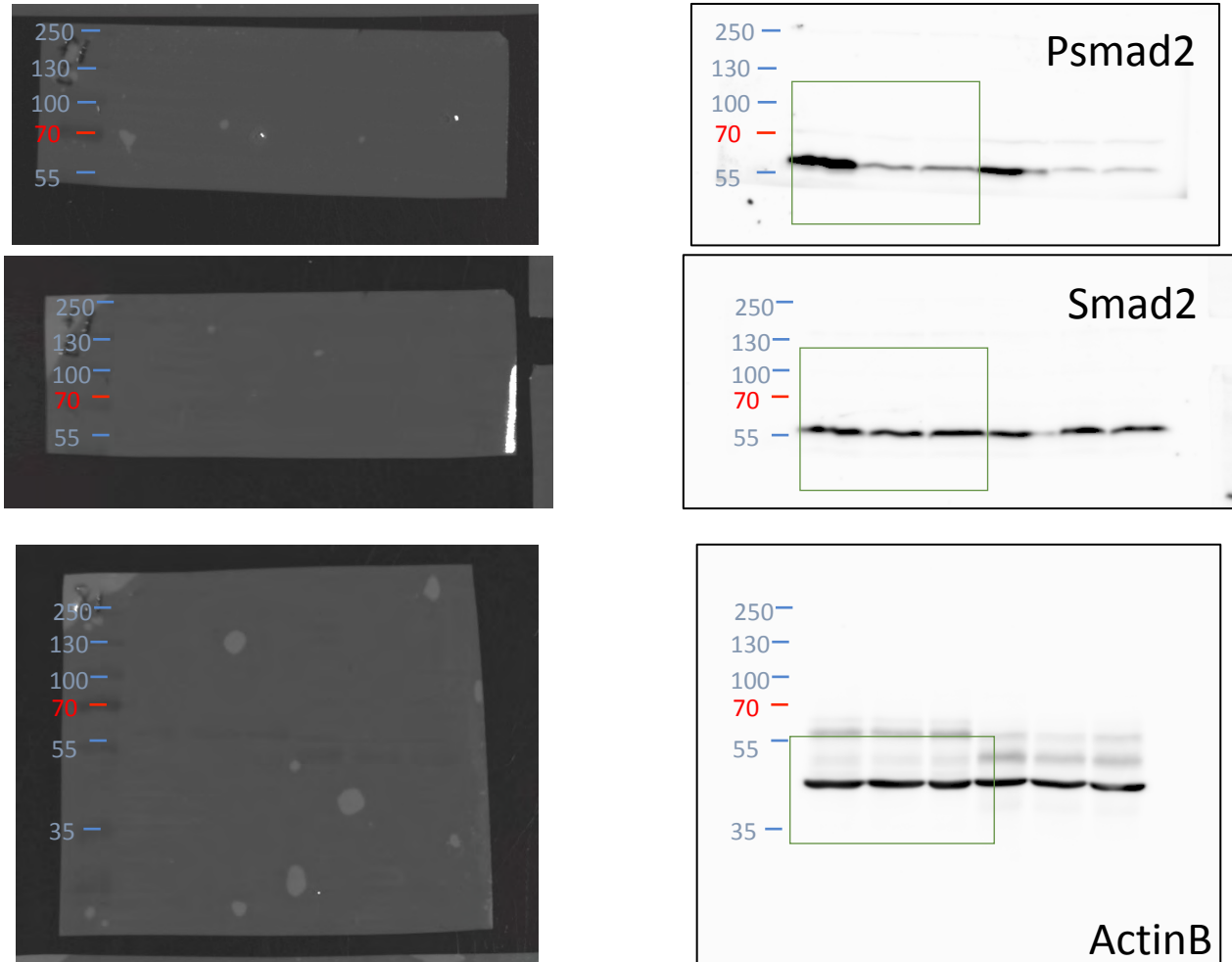

## D283 D L S WB40\_ PSMAD

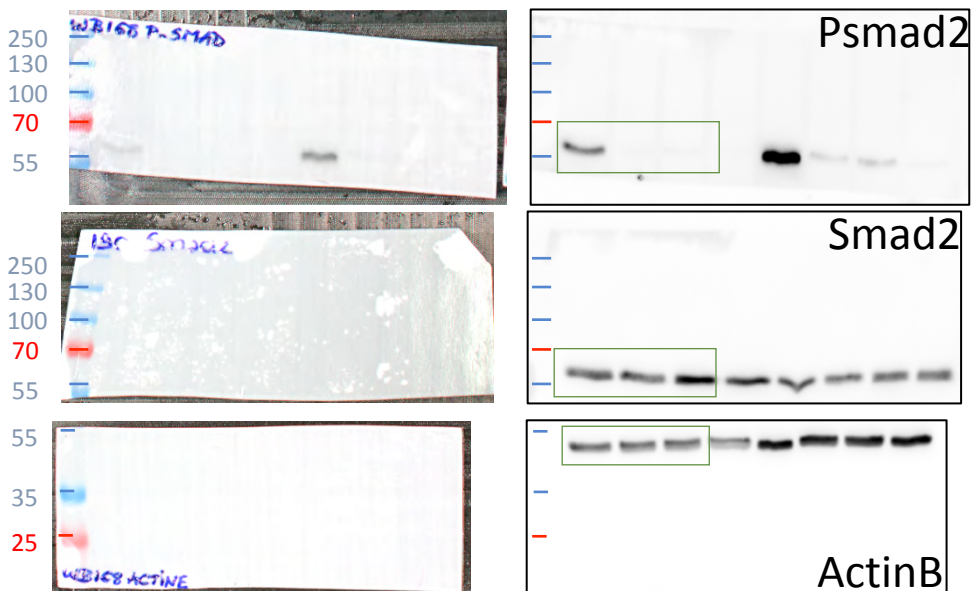

Supplement: Supplementary file 8 — Source Data for Figure 4 [file EMMM-11-e9830-s006.pdf]
