# Supplementary material for: An autocrine ActivinB mechanism drives TGFβ/Activin signaling in Group 3 medulloblastoma
Source: EMBO Mol Med. 2019 Jul 22;11(8):e9830. doi: 10.15252/emmm.201809830 (PMC6685082; doi:10.15252/emmm.201809830)

Figure 1A

Patient samples

Patient samples WNT (262) and SHH (263)

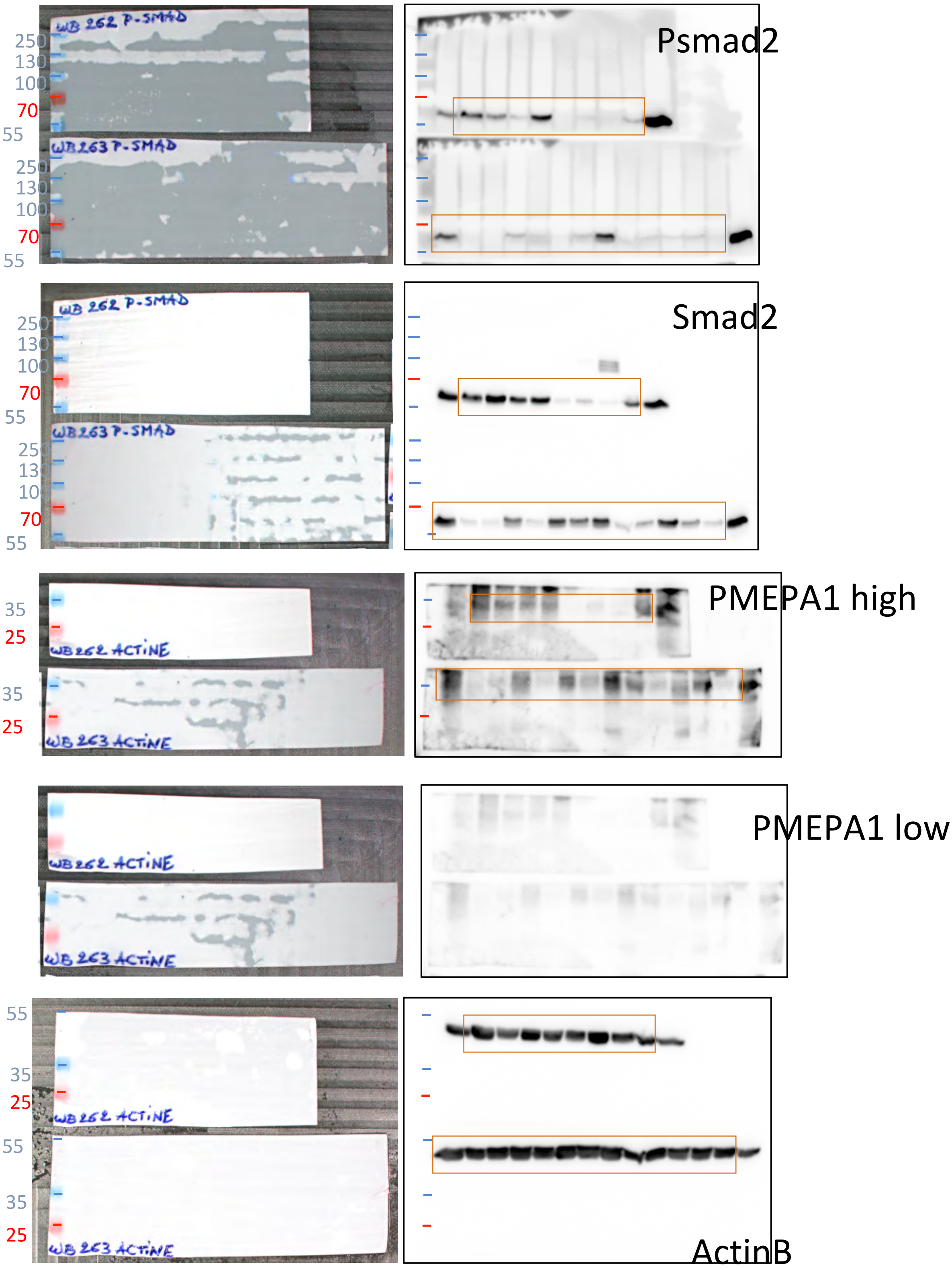

Patient samples Gpe3 (292) and Gpe4 (293)

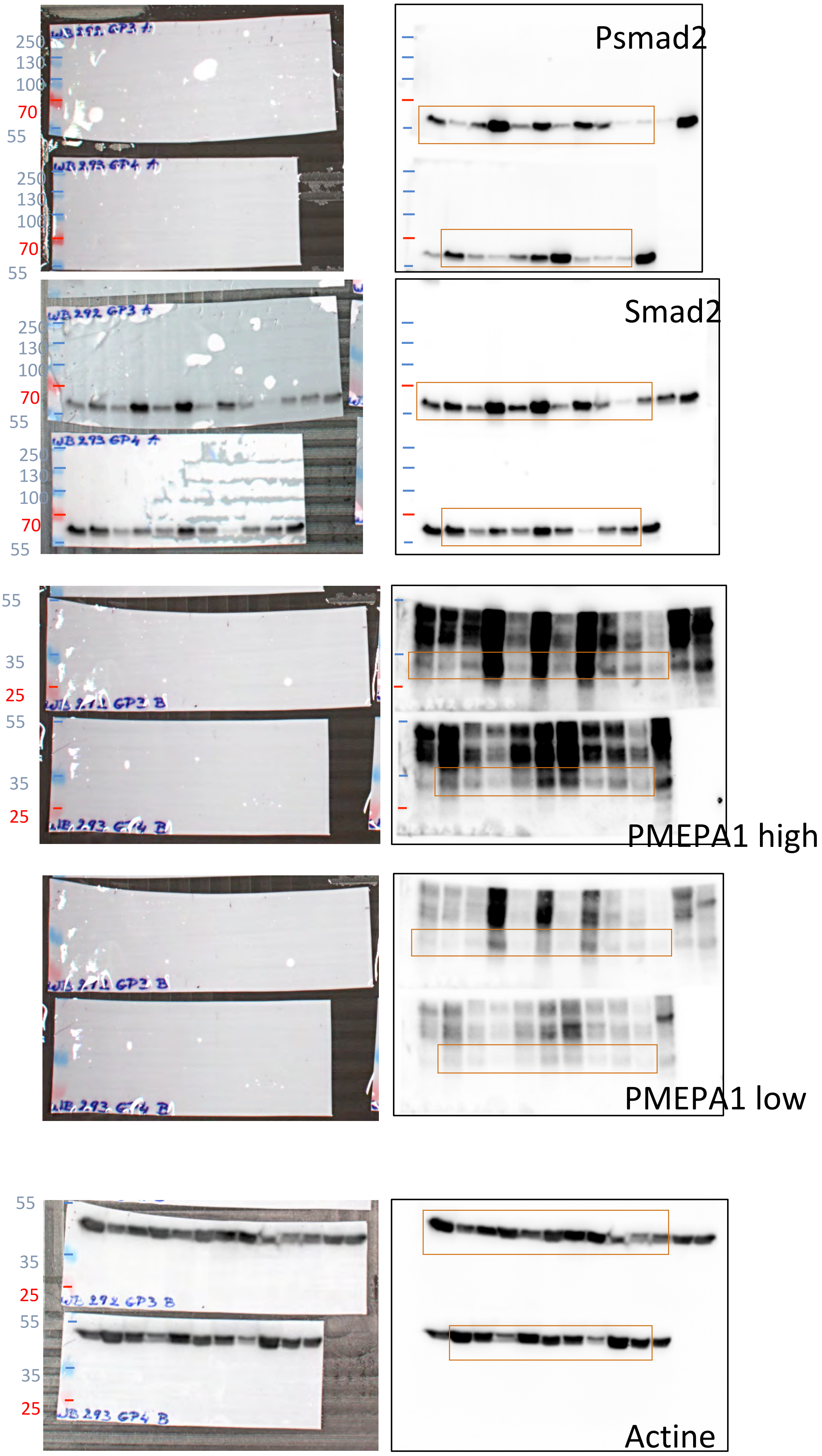

Figure 1C

All MB cell lines

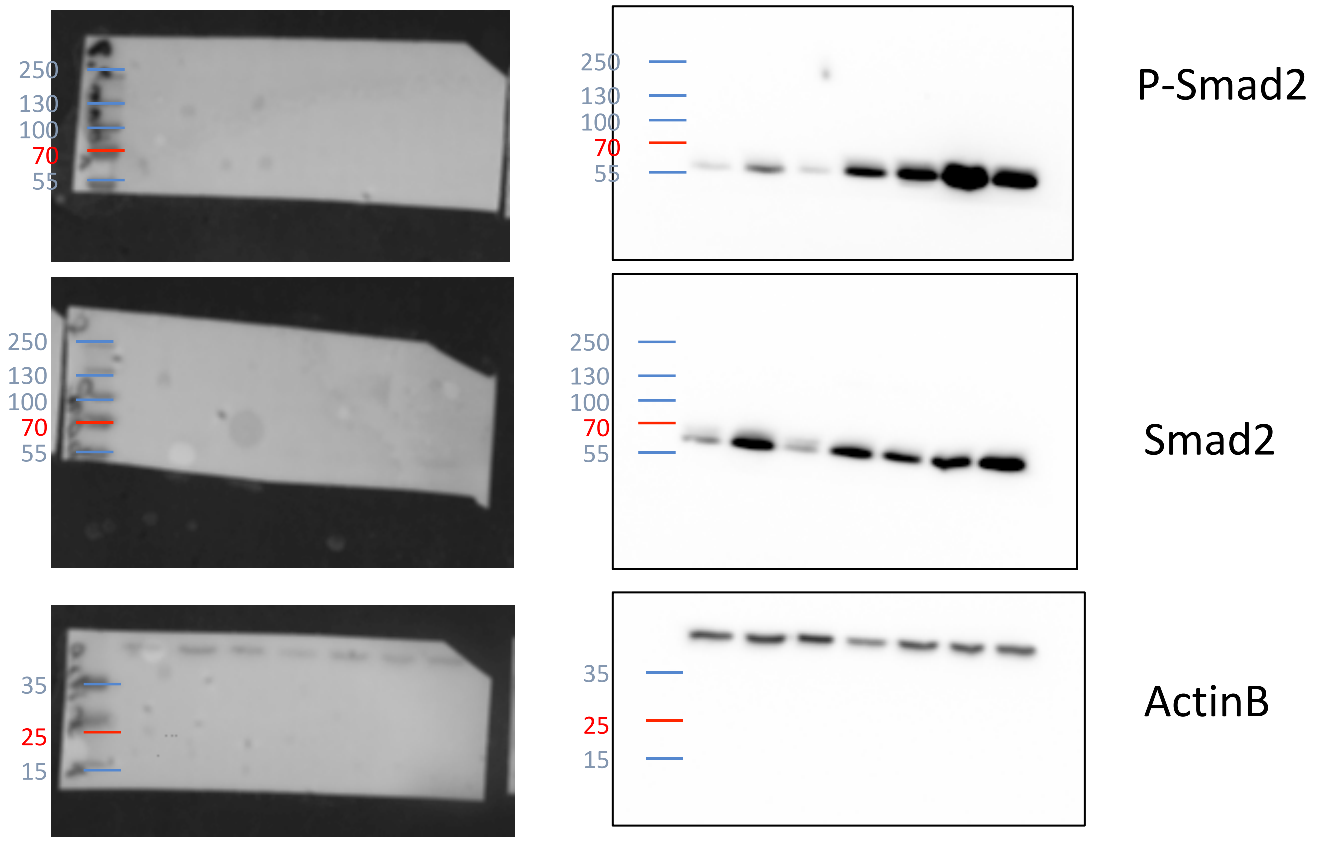

Supplement: Supplementary file 5 — Source Data for Figure 1 [file EMMM-11-e9830-s003.pdf]
