# Supplementary material for: An autocrine ActivinB mechanism drives TGFβ/Activin signaling in Group 3 medulloblastoma
Source: EMBO Mol Med. 2019 Jul 22;11(8):e9830. doi: 10.15252/emmm.201809830 (PMC6685082; doi:10.15252/emmm.201809830)

# Figure 2A

Cell lines

Activin or TGFb stimulations

# 2A – 10/3/16 DAOY

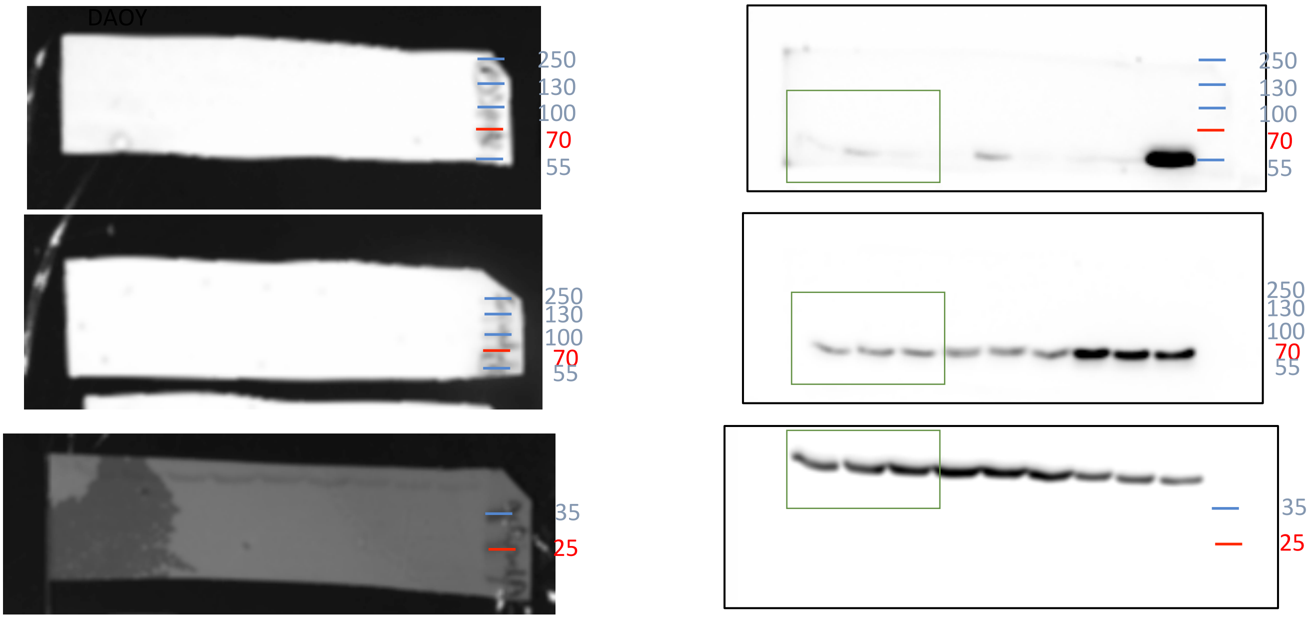


# 2A – ONS76

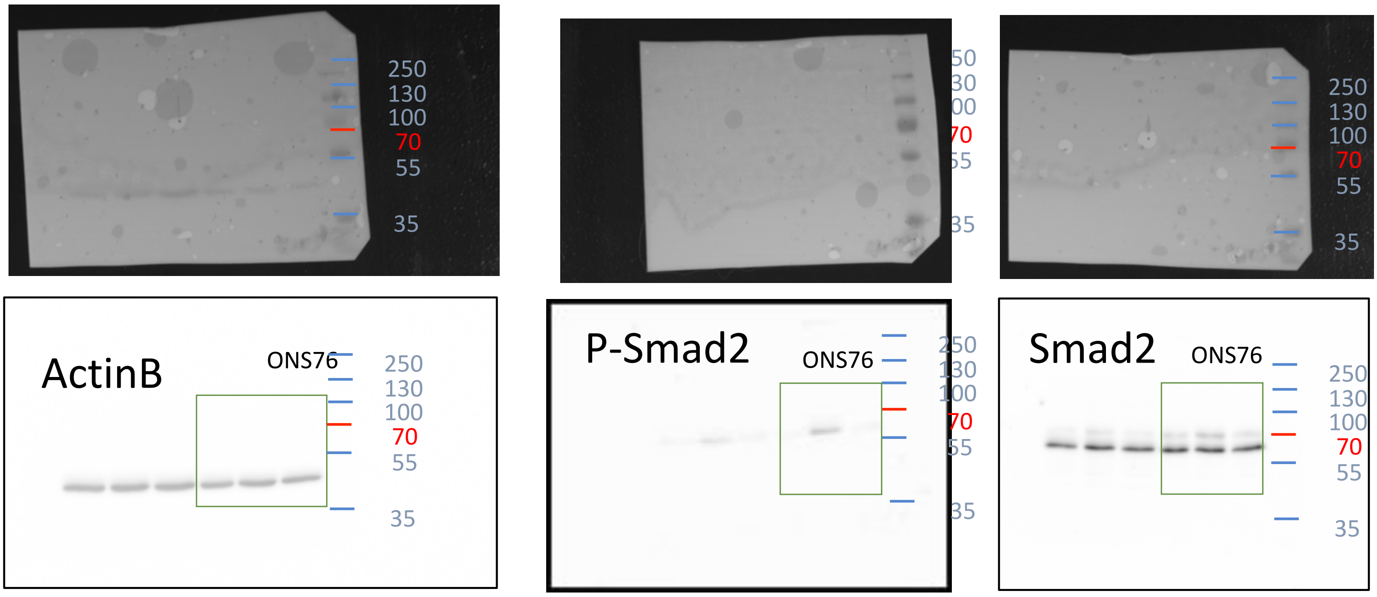

# 2A – UW228 – D283

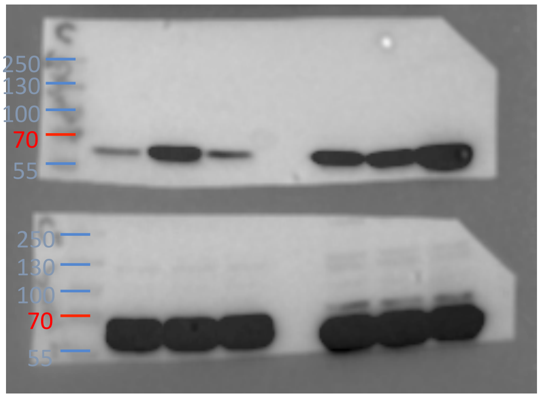


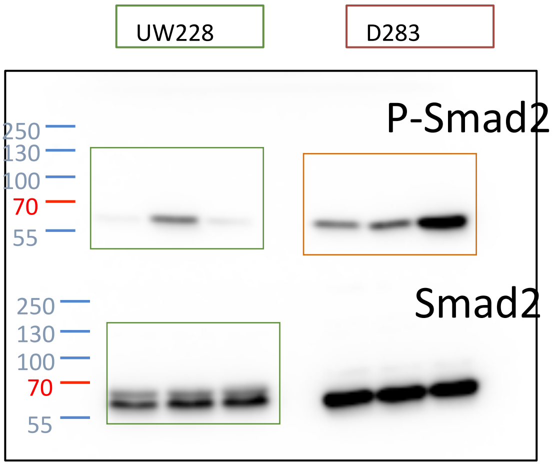


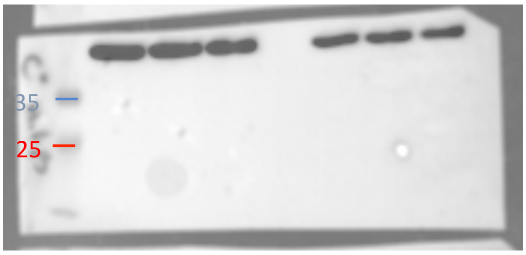


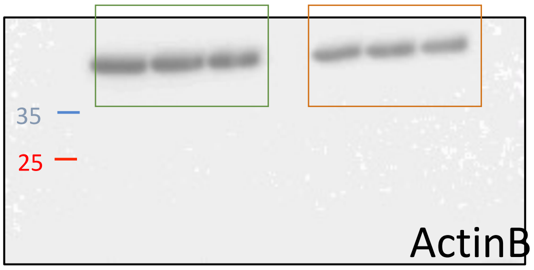


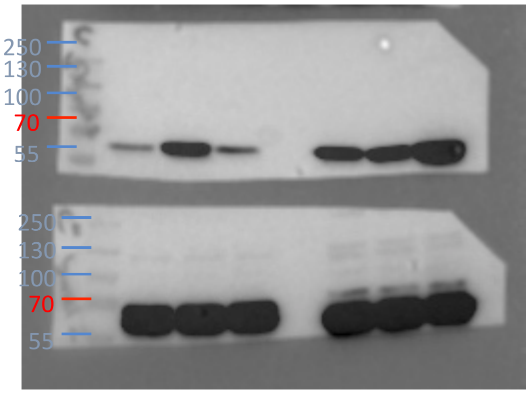


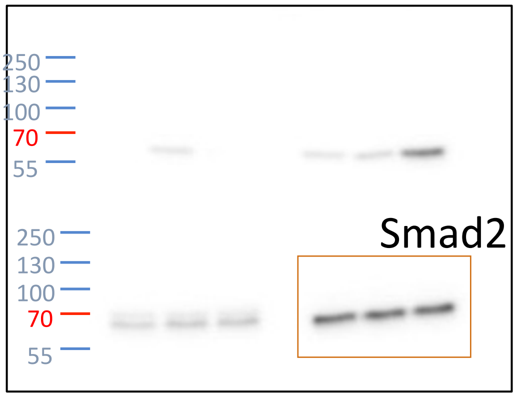

## HDMB03 ET D458 P/T/A WB35

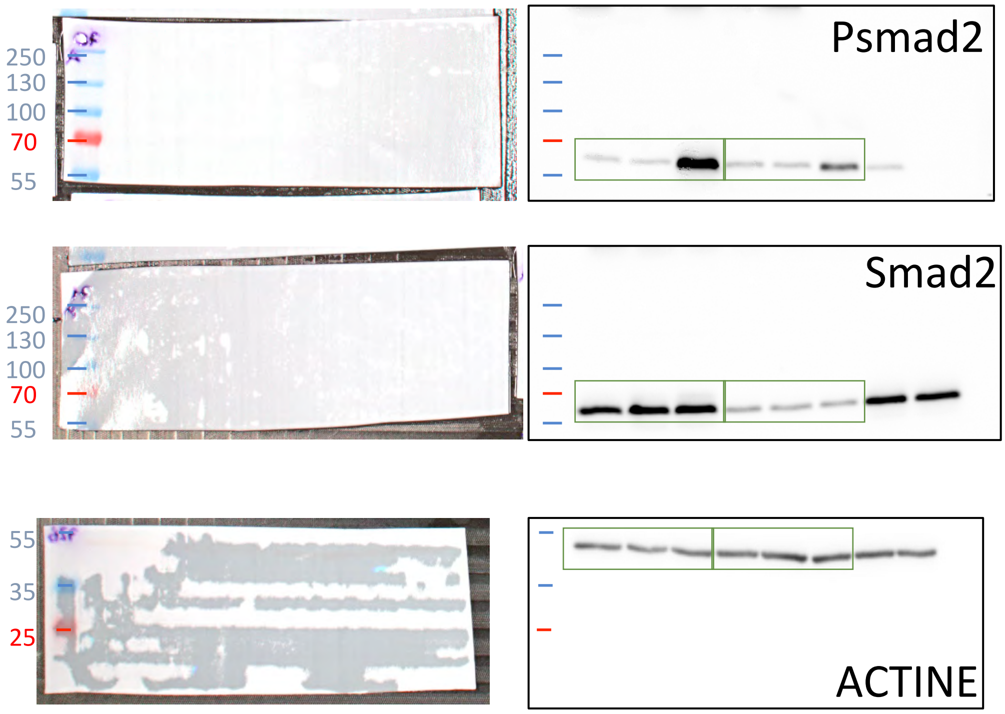


## 1603 P T A

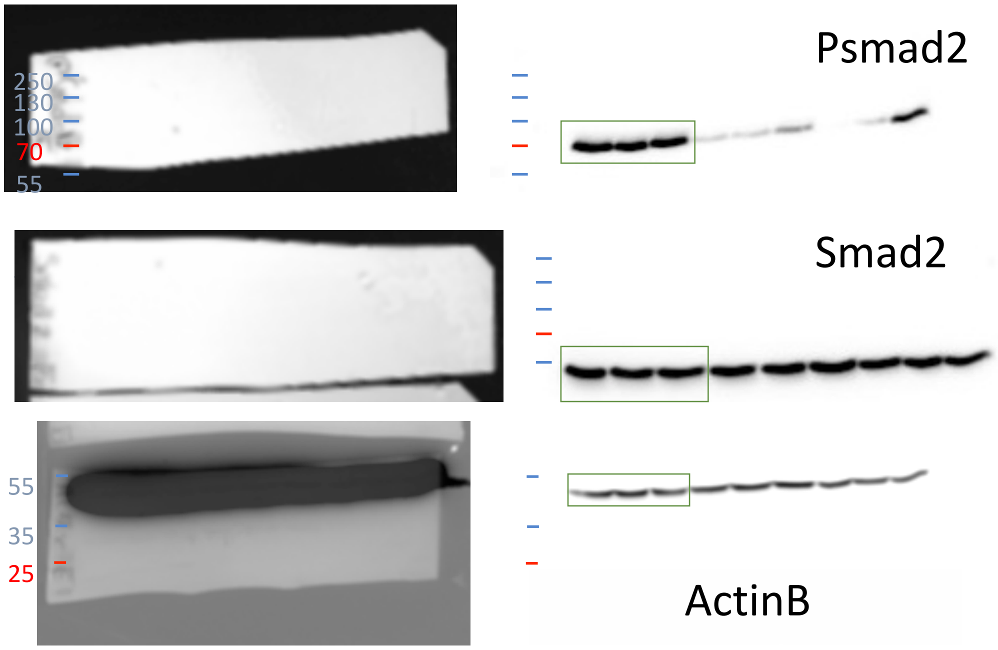

Figure 2B

## 1603 Ab

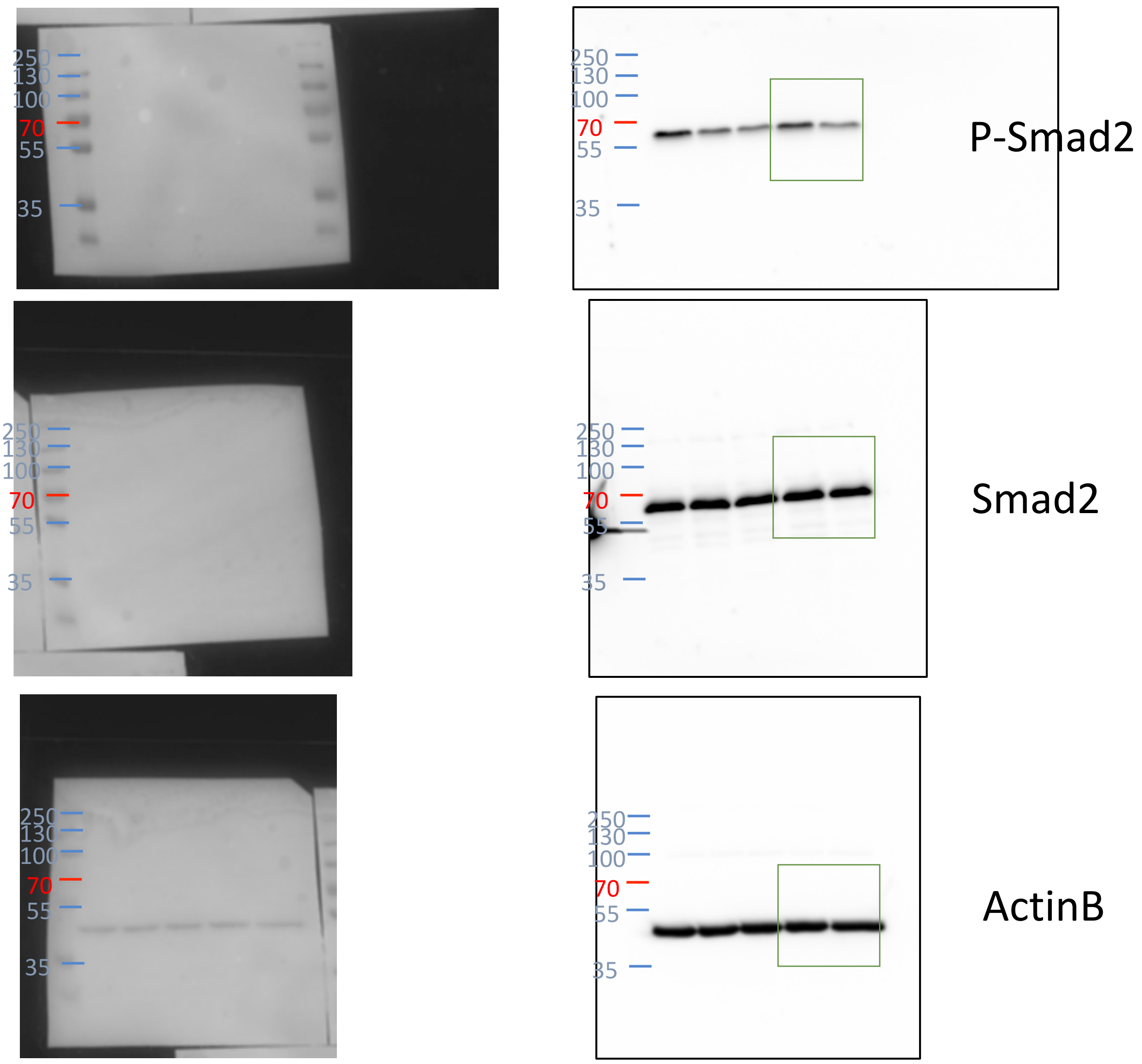


## 1603 FLT WB 31

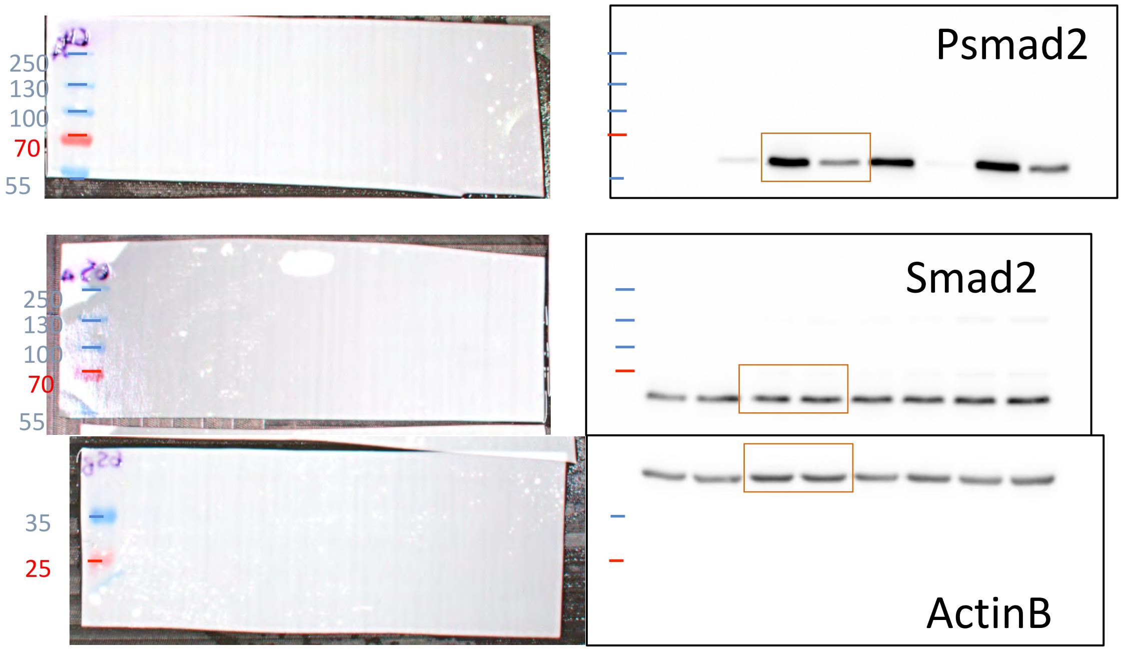

# Figure 2C and E

conditionned media and siINHBB

**2C**

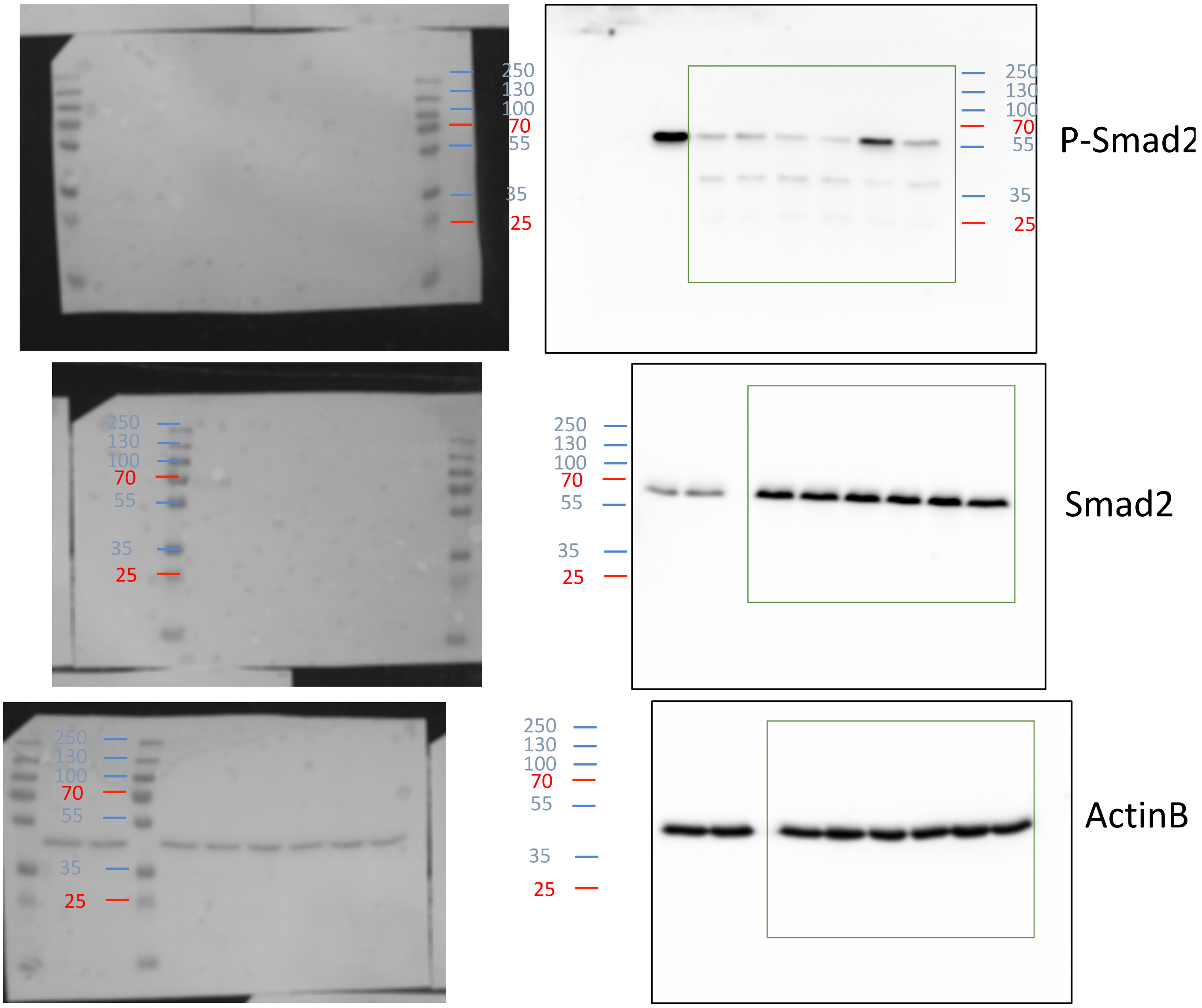

**2E**

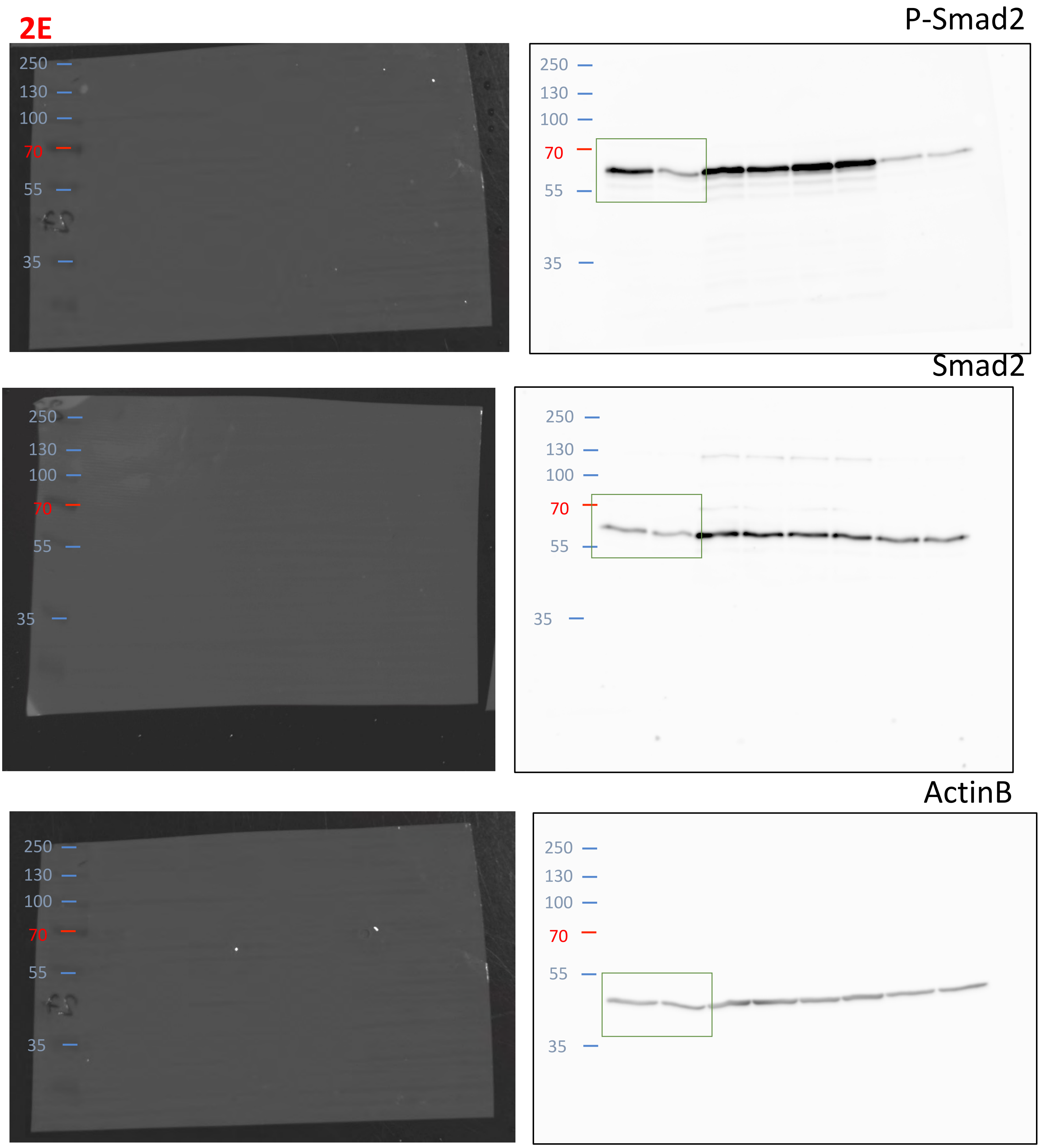

Supplement: Supplementary file 6 — Source Data for Figure 2 [file EMMM-11-e9830-s004.pdf]
