# Supplementary material for: An autocrine ActivinB mechanism drives TGFβ/Activin signaling in Group 3 medulloblastoma
Source: EMBO Mol Med. 2019 Jul 22;11(8):e9830. doi: 10.15252/emmm.201809830 (PMC6685082; doi:10.15252/emmm.201809830)

Figure 5F

D/L/S and P/A

1603 D L S WB93_ TARGET GENES

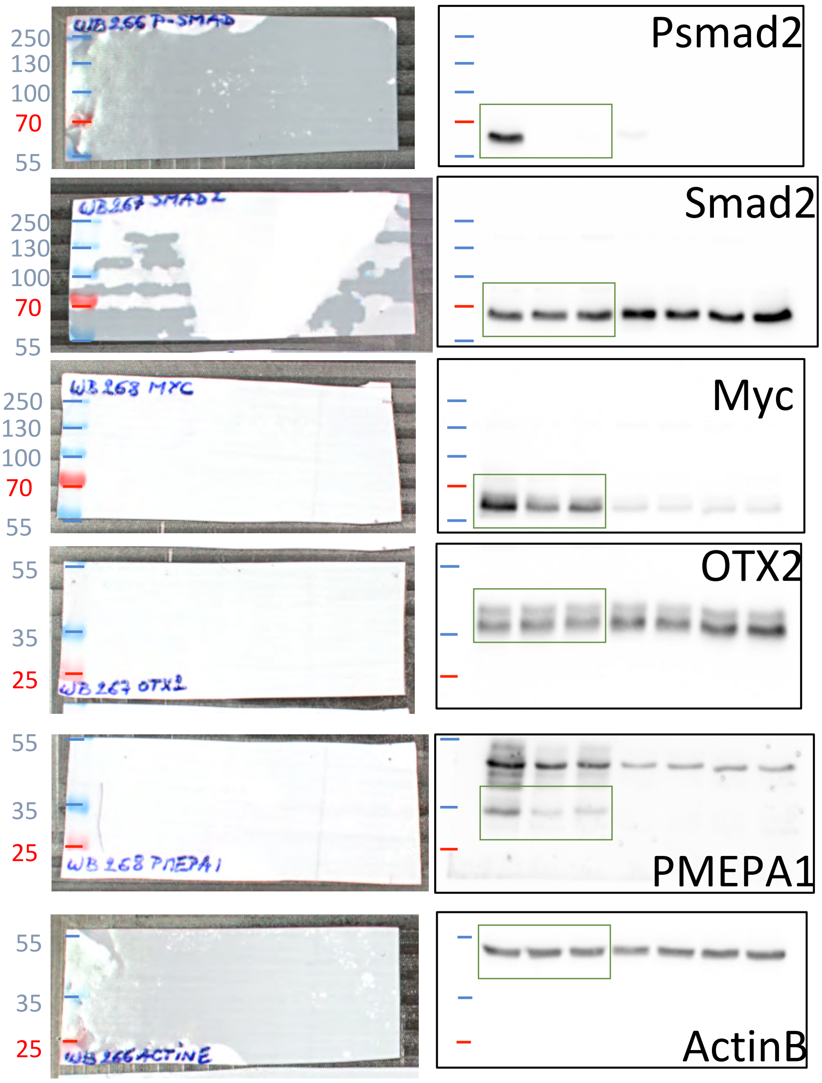

D283 PA 1603 Ab WB 11

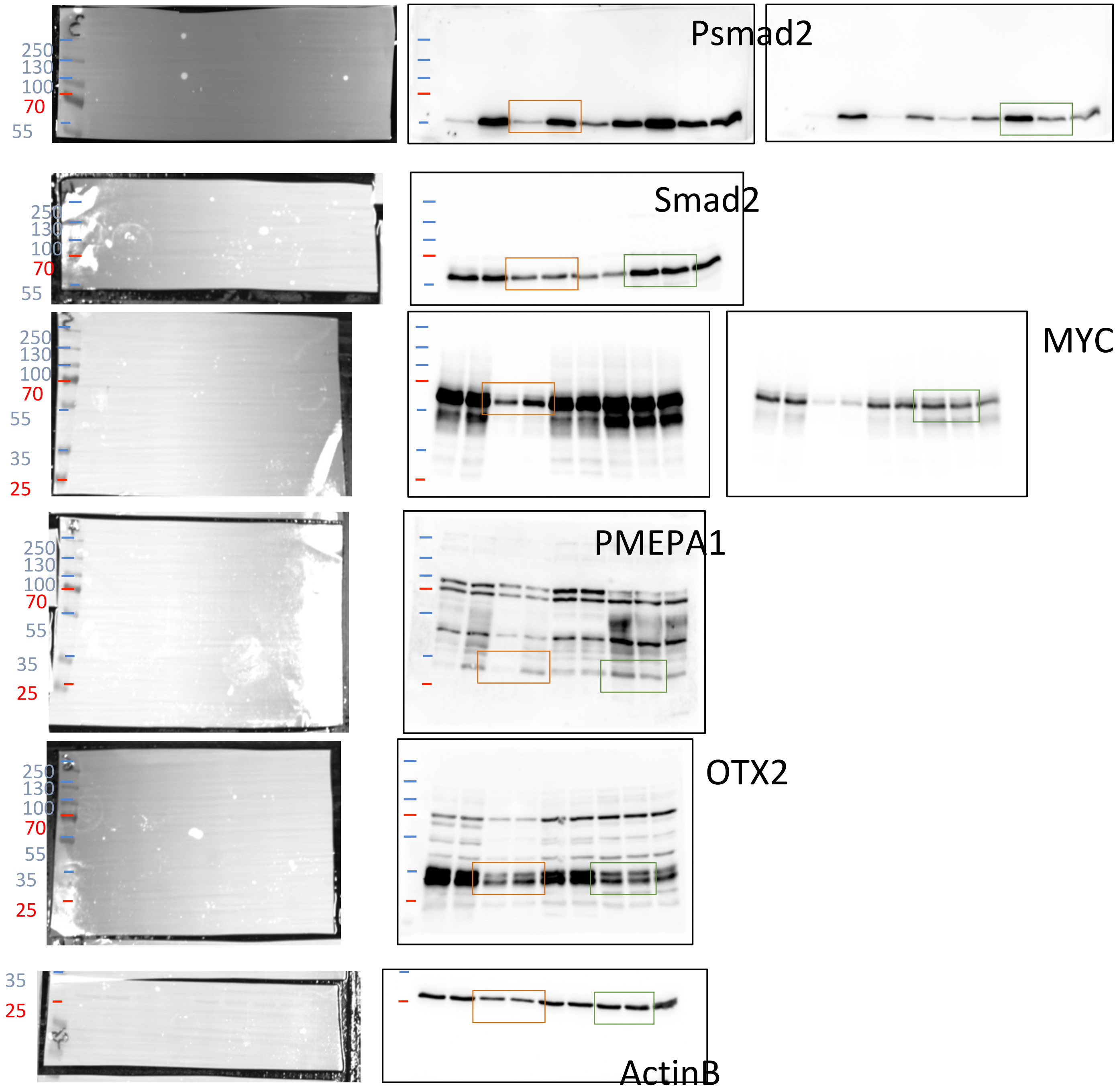

1603 FLT WB 33 TARGET

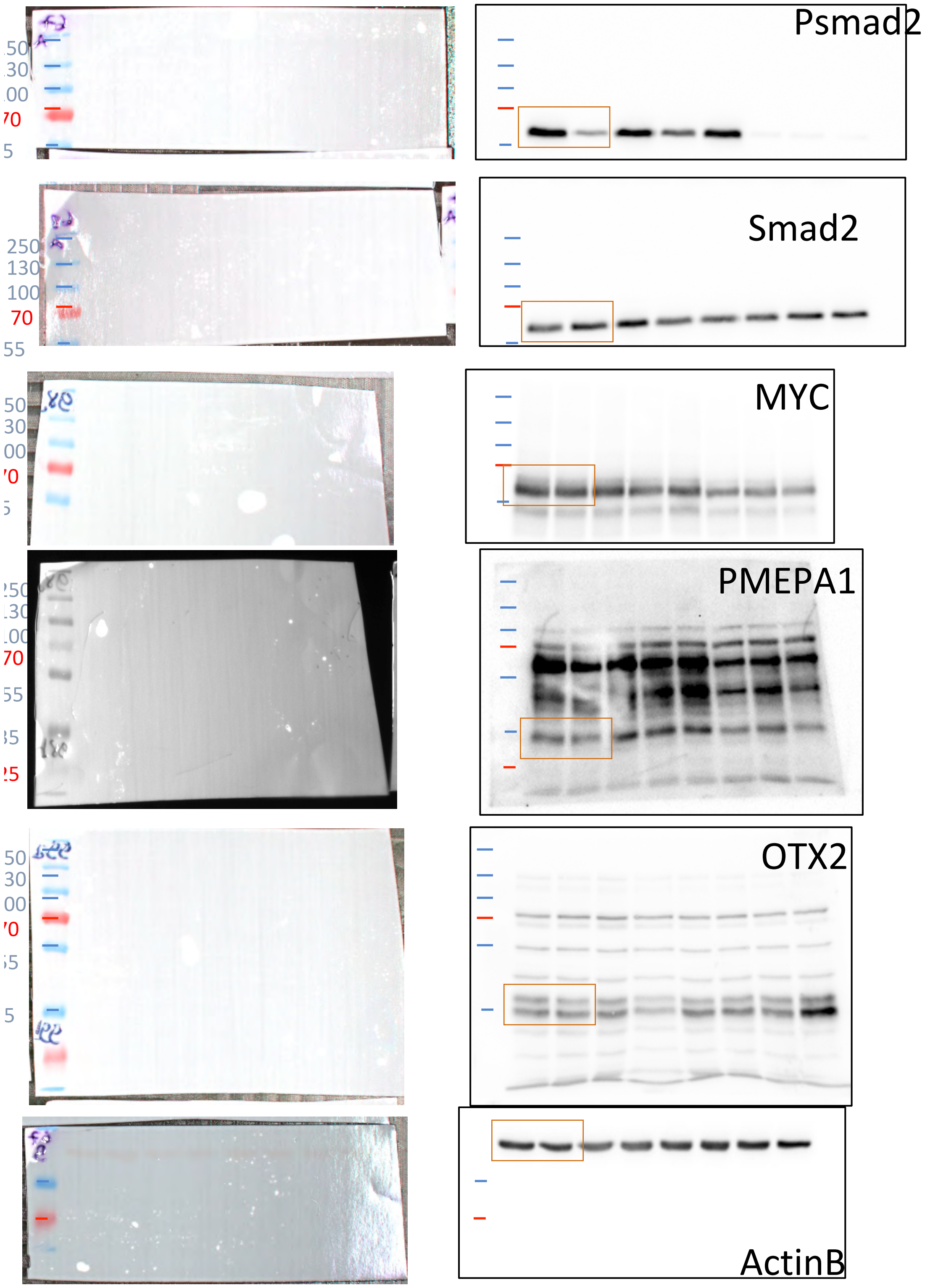

d458 PA TARGET GENES WB29_ PSMAD

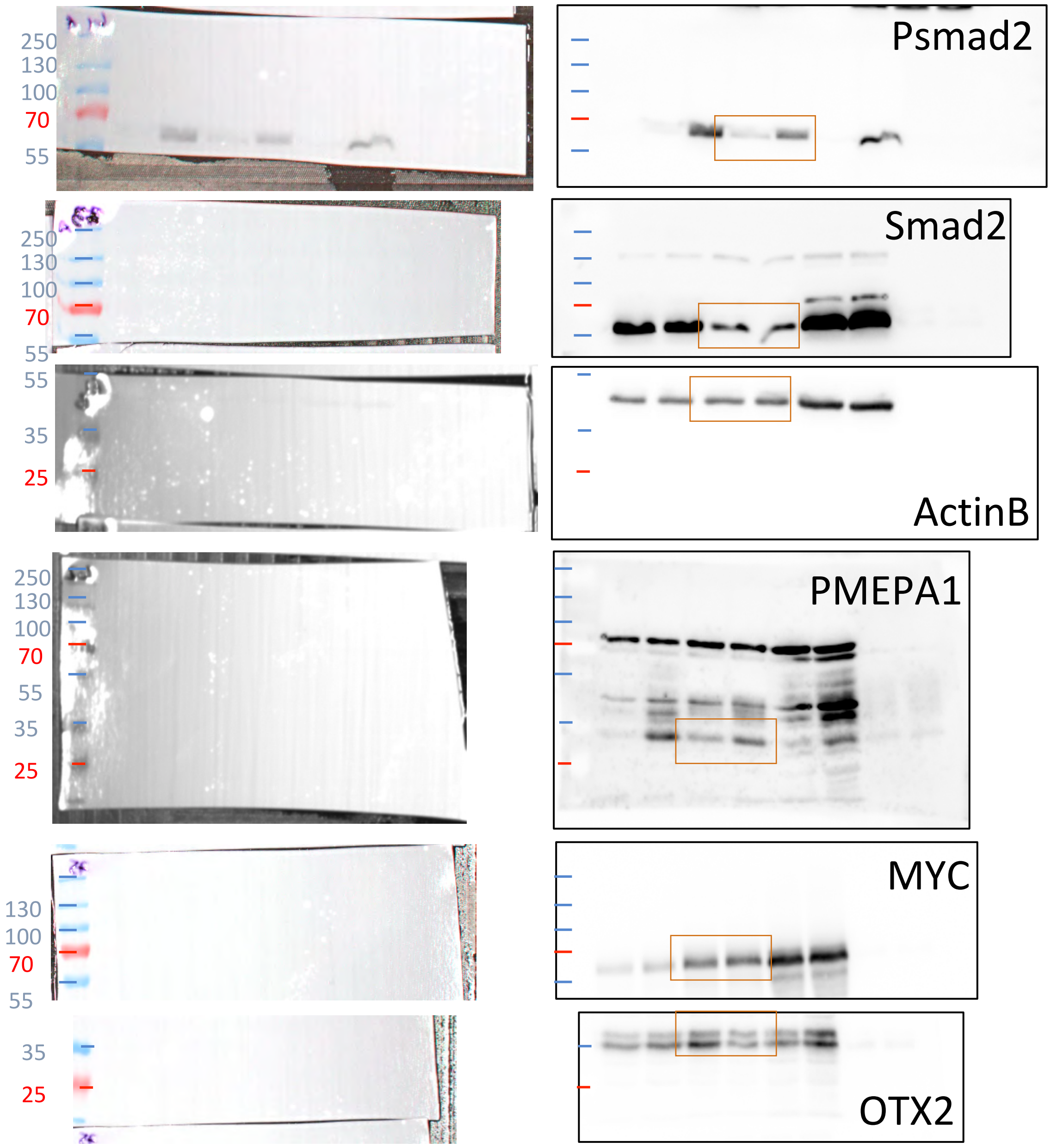

d283 DLSG TARGET GENES _ PSMAD

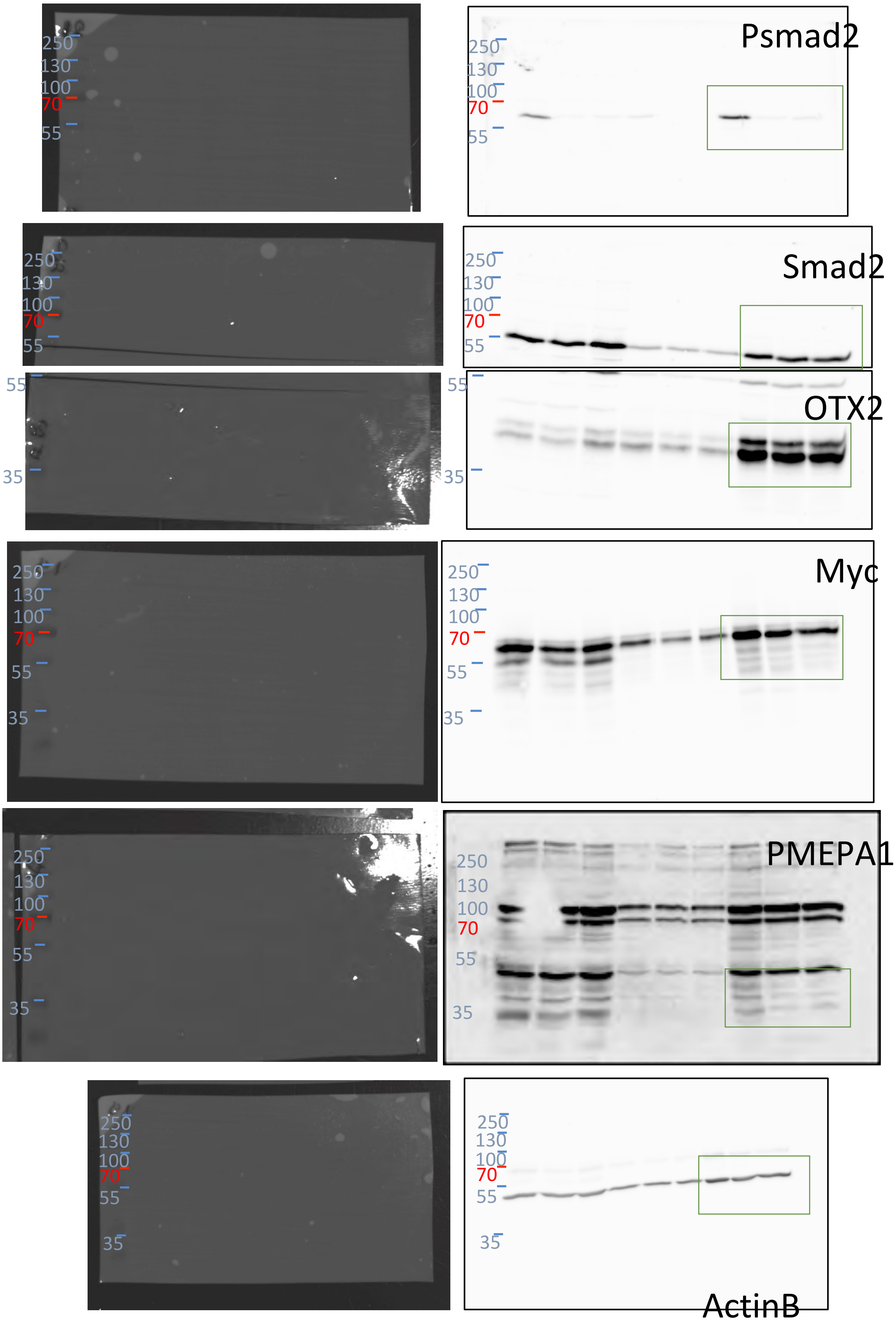

Figure 5G and I

SiPMEPA1 1603MED and D283

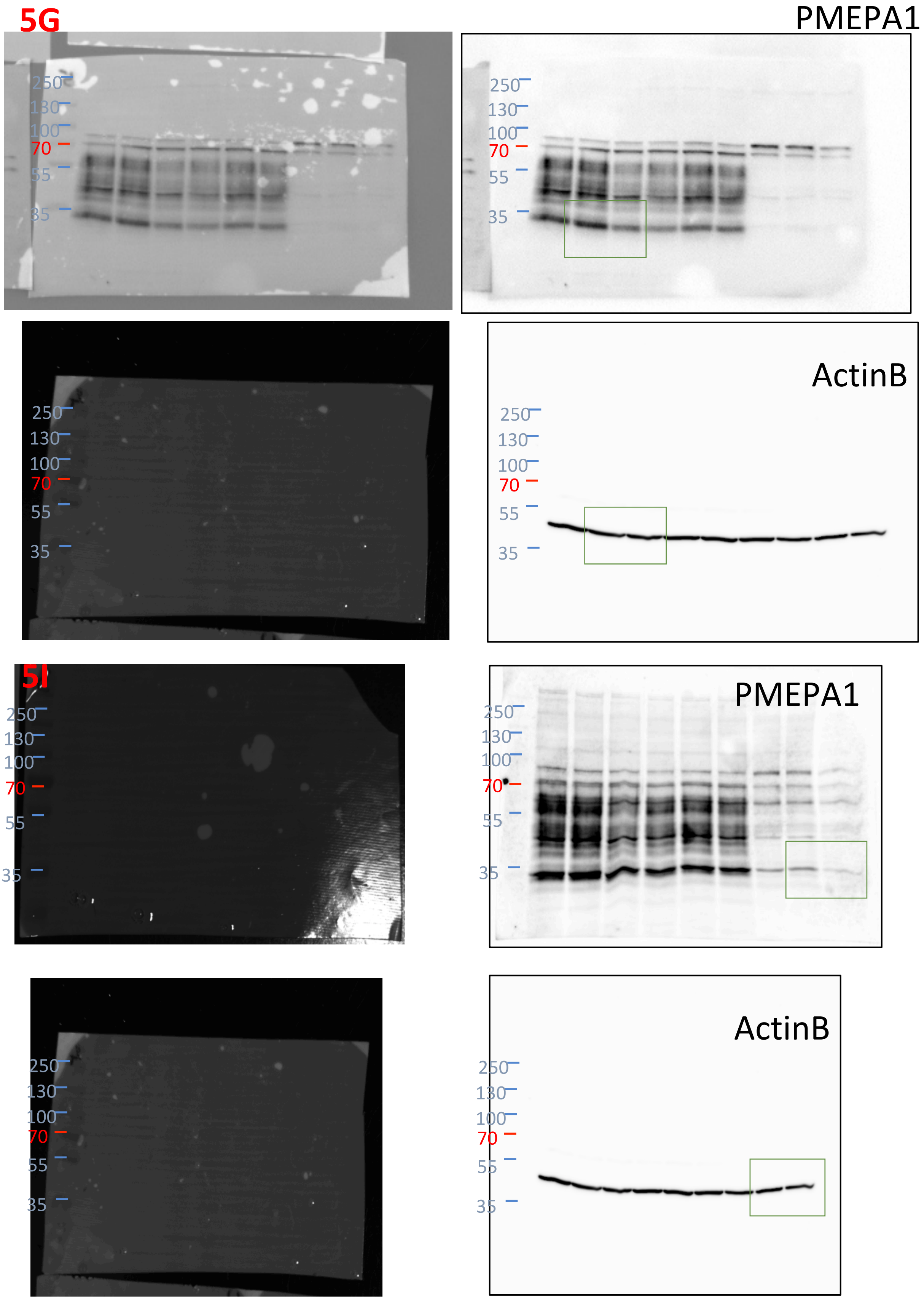

Supplement: Supplementary file 9 — Source Data for Figure 5 [file EMMM-11-e9830-s007.pdf]
