# Supplementary material for: An autocrine ActivinB mechanism drives TGFβ/Activin signaling in Group 3 medulloblastoma
Source: EMBO Mol Med. 2019 Jul 22;11(8):e9830. doi: 10.15252/emmm.201809830 (PMC6685082; doi:10.15252/emmm.201809830)
Supplement: Supplementary file 10 — Source Data for Figure 6 [file EMMM-11-e9830-s008.pdf]

Figure 6A

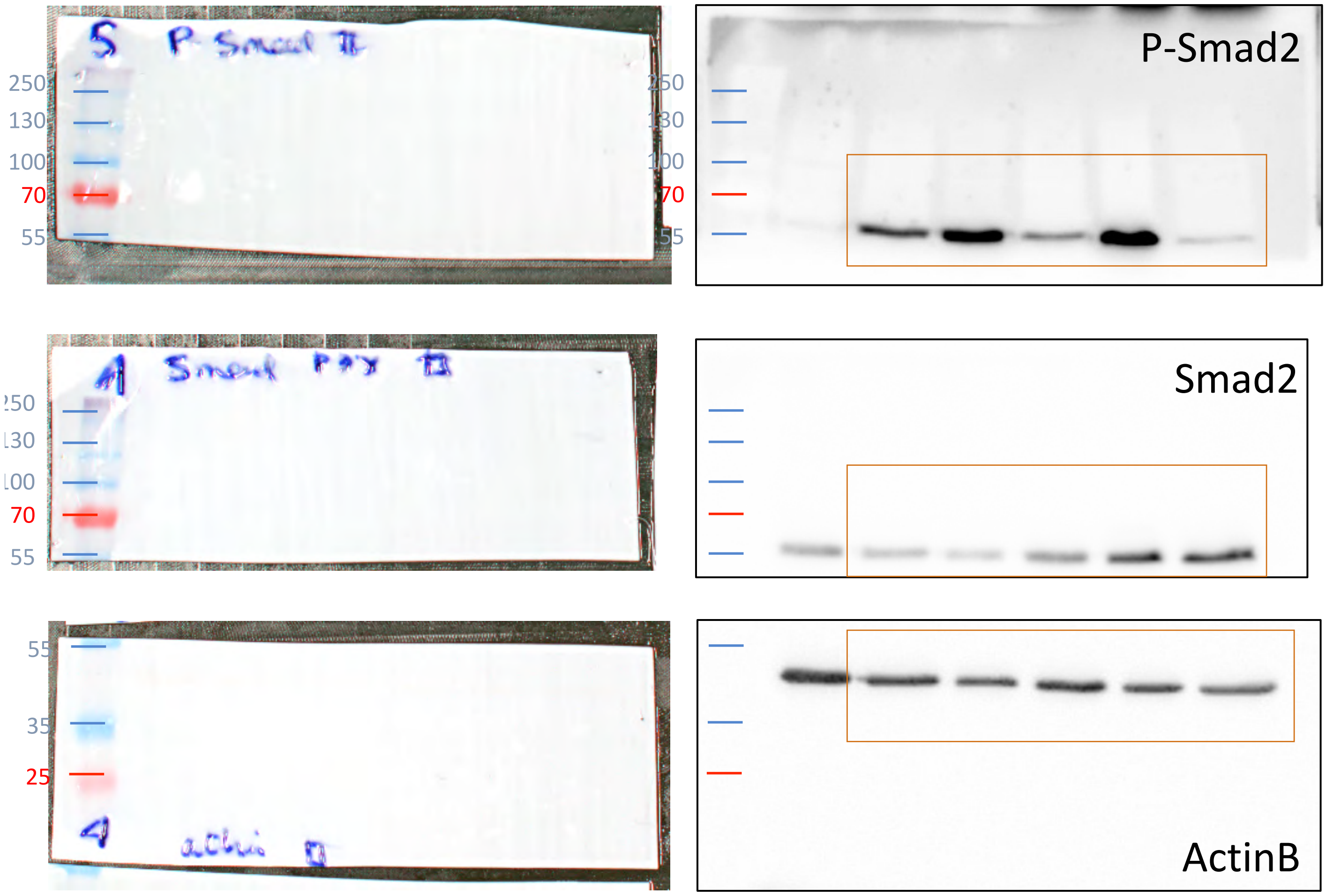

Figure 6D

MC PDX4

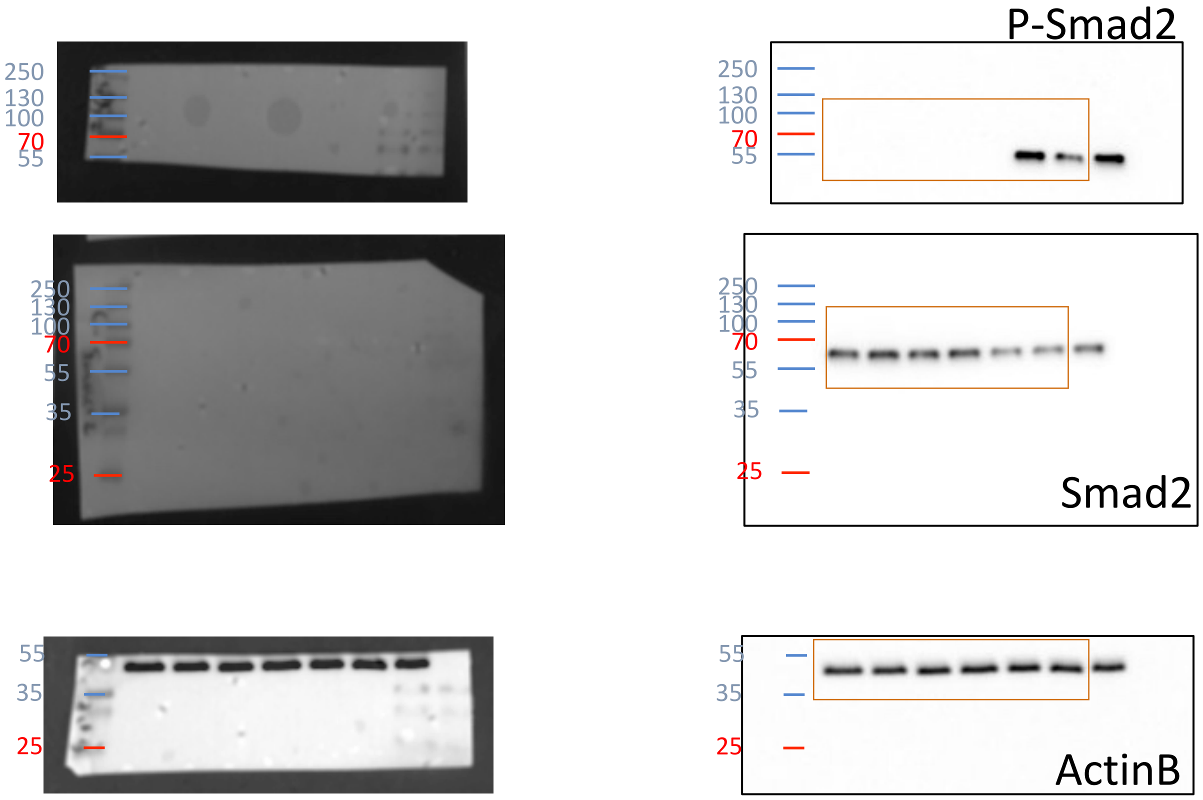

Figure 6C

6C-PDX3

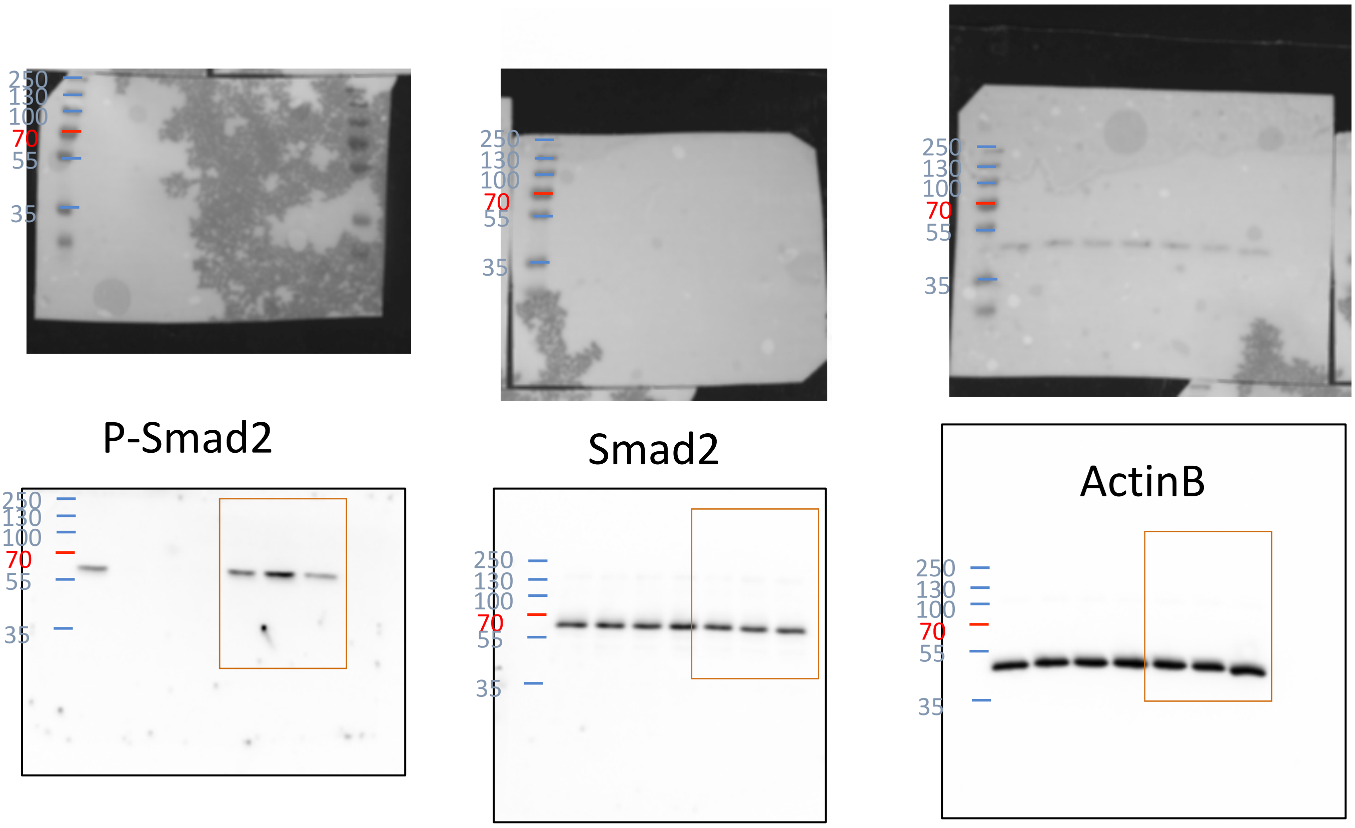

6C-PDX7

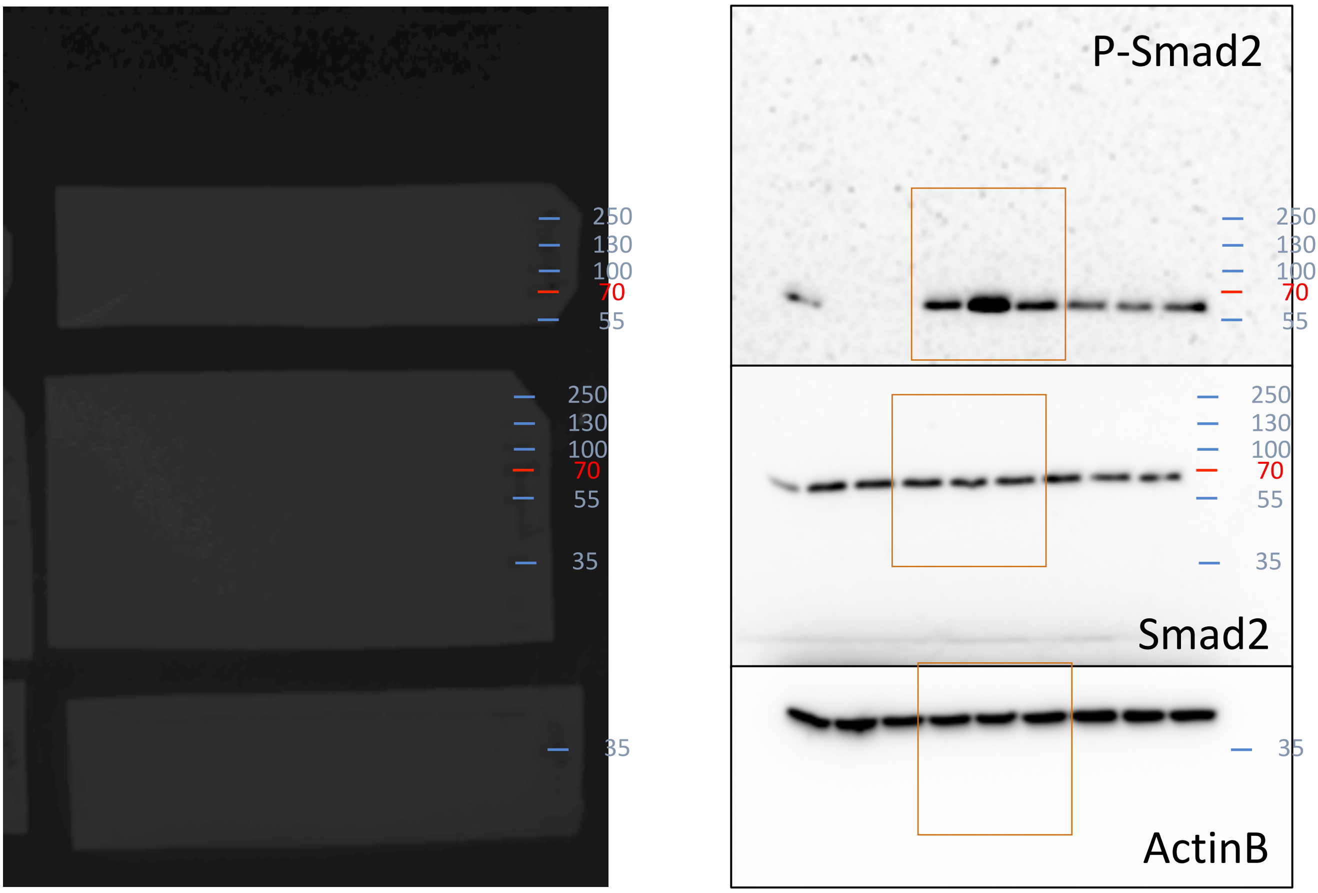

6C-PDX4

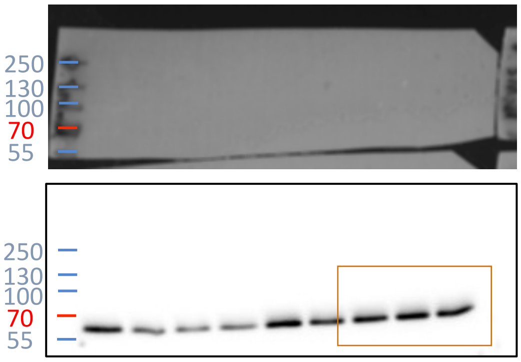

P-Smad2

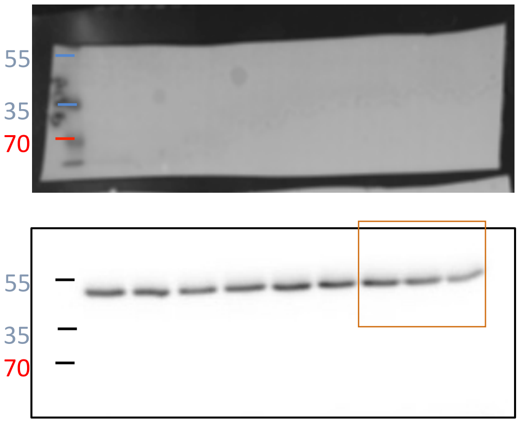

ActinB

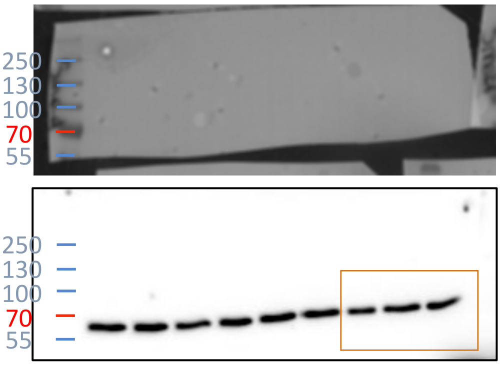

Smad2

Figure 6E

PDX4_DLS_WB64

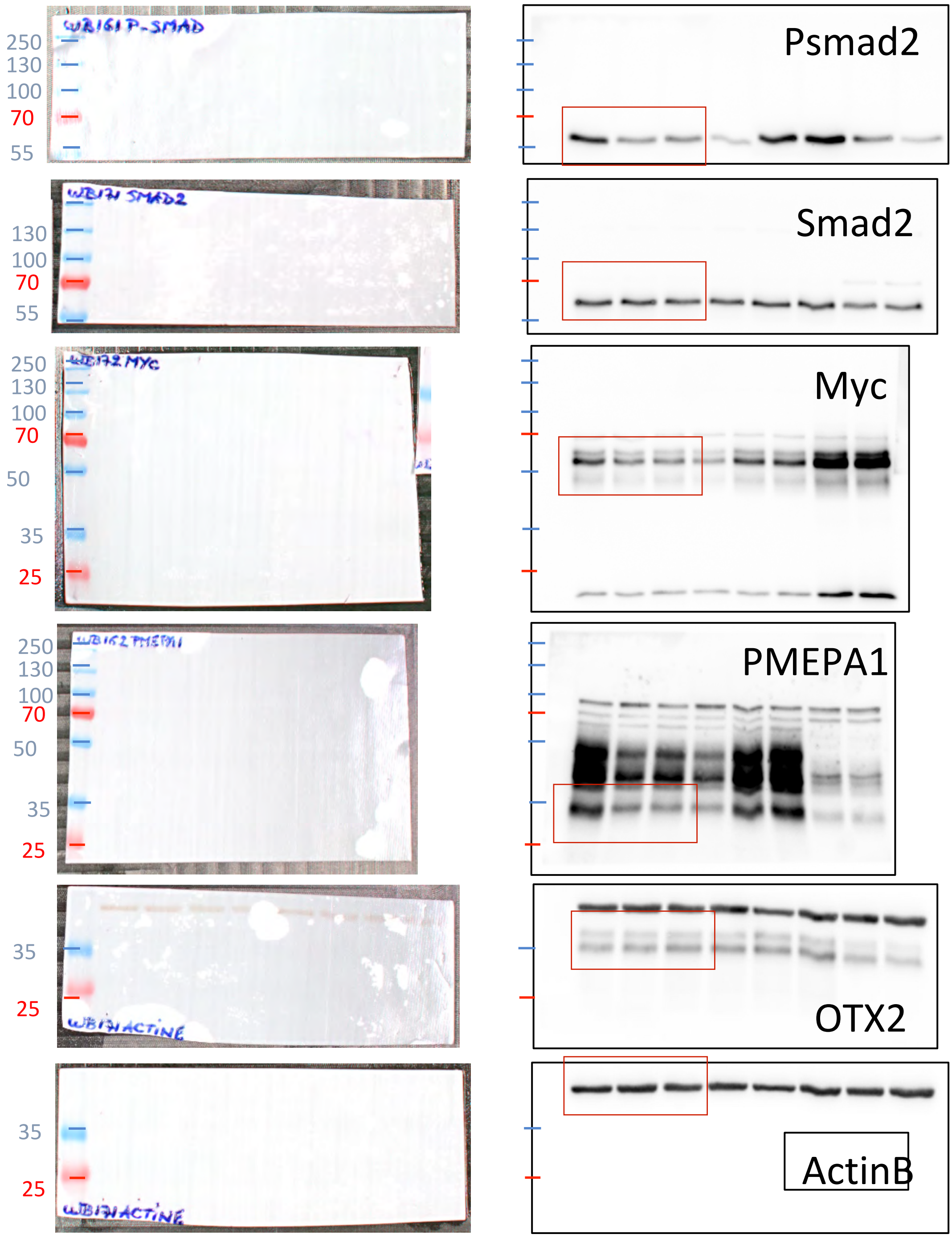

PDX4_Ab_WB99

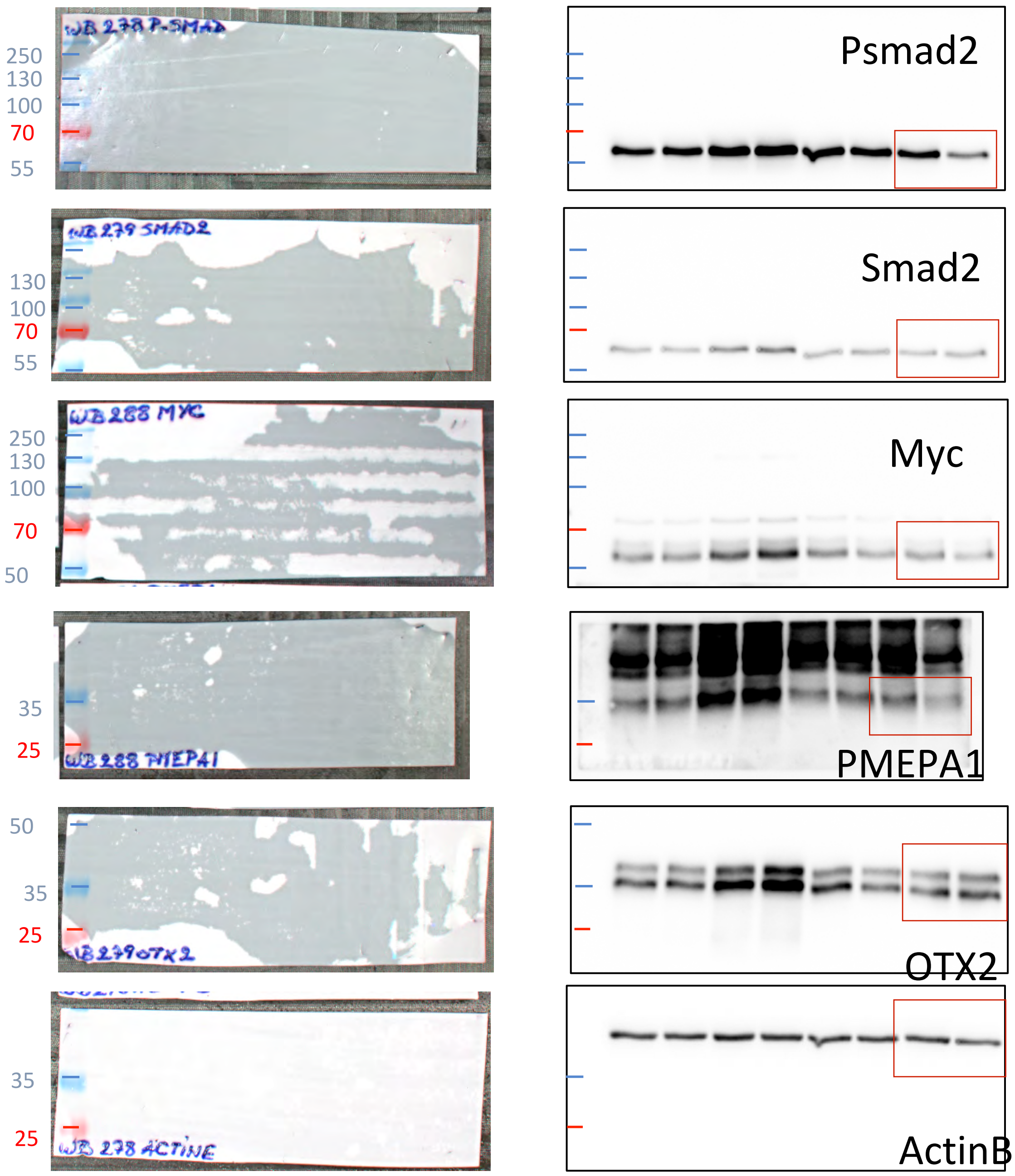
250
130
100
70
55
WB 278 P-SMAD
Psmad2
WB 279 SMAD2
130
100
70
55
Smad2
WB 288 MYC
250
130
100
70
50
Myc
35
25
WB 288 PMEPA1
PMEPA1
50
35
25
WB 279 OTX2
OTX2
35
25
WB 278 ACTINE
ActinB

-PDX4_FLST_WB99

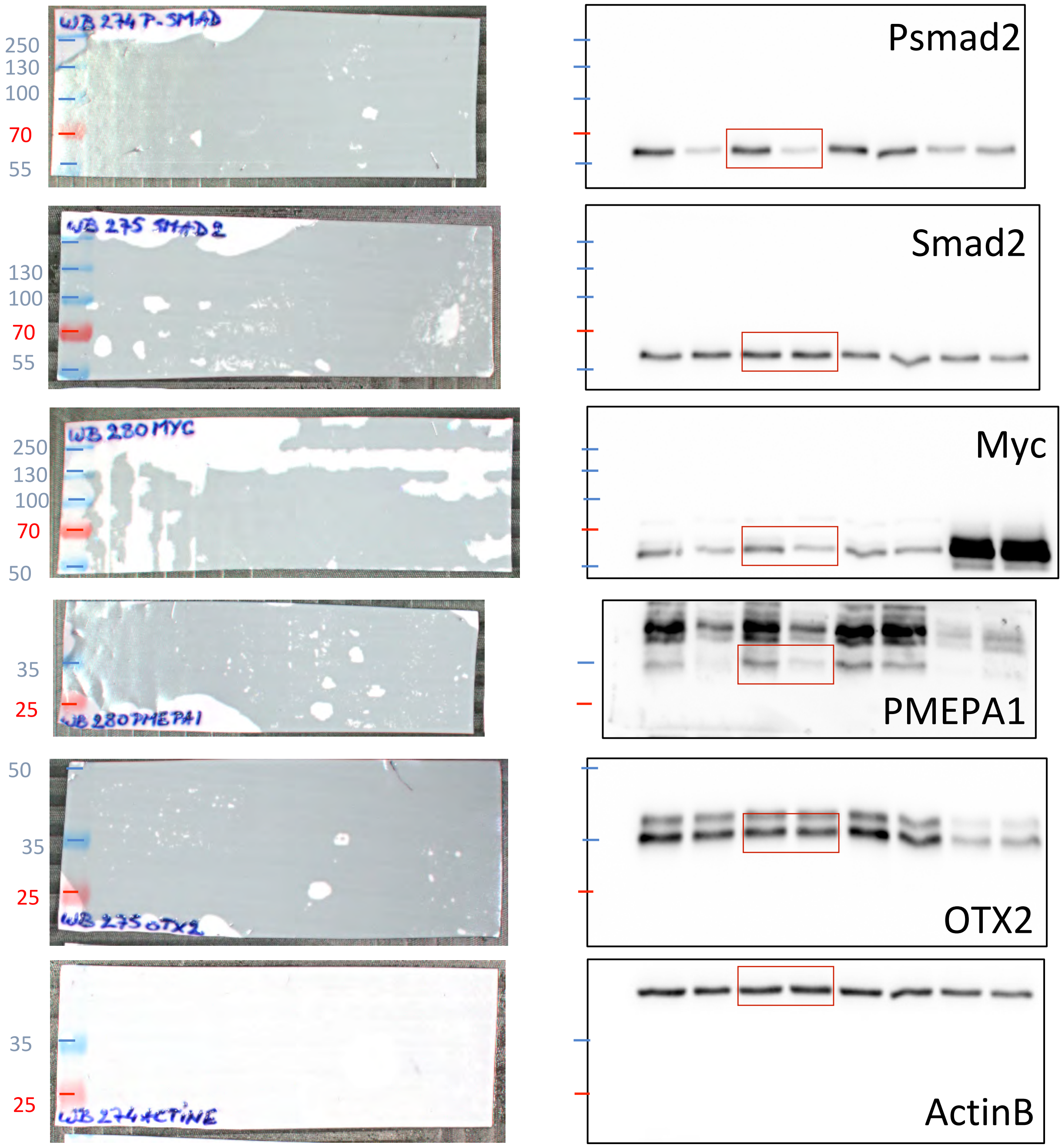

PDX3_DLS WB68

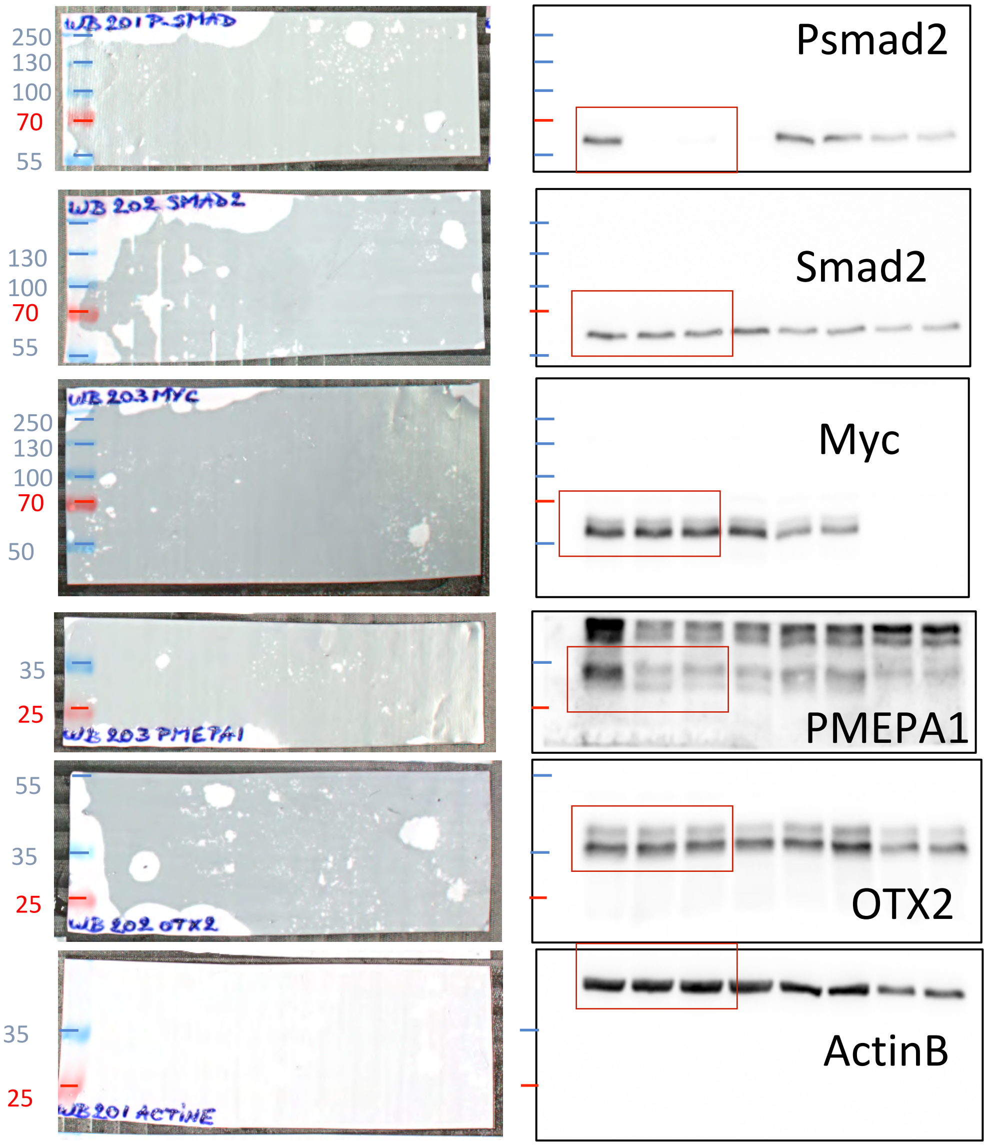

PDX3_PA WB108

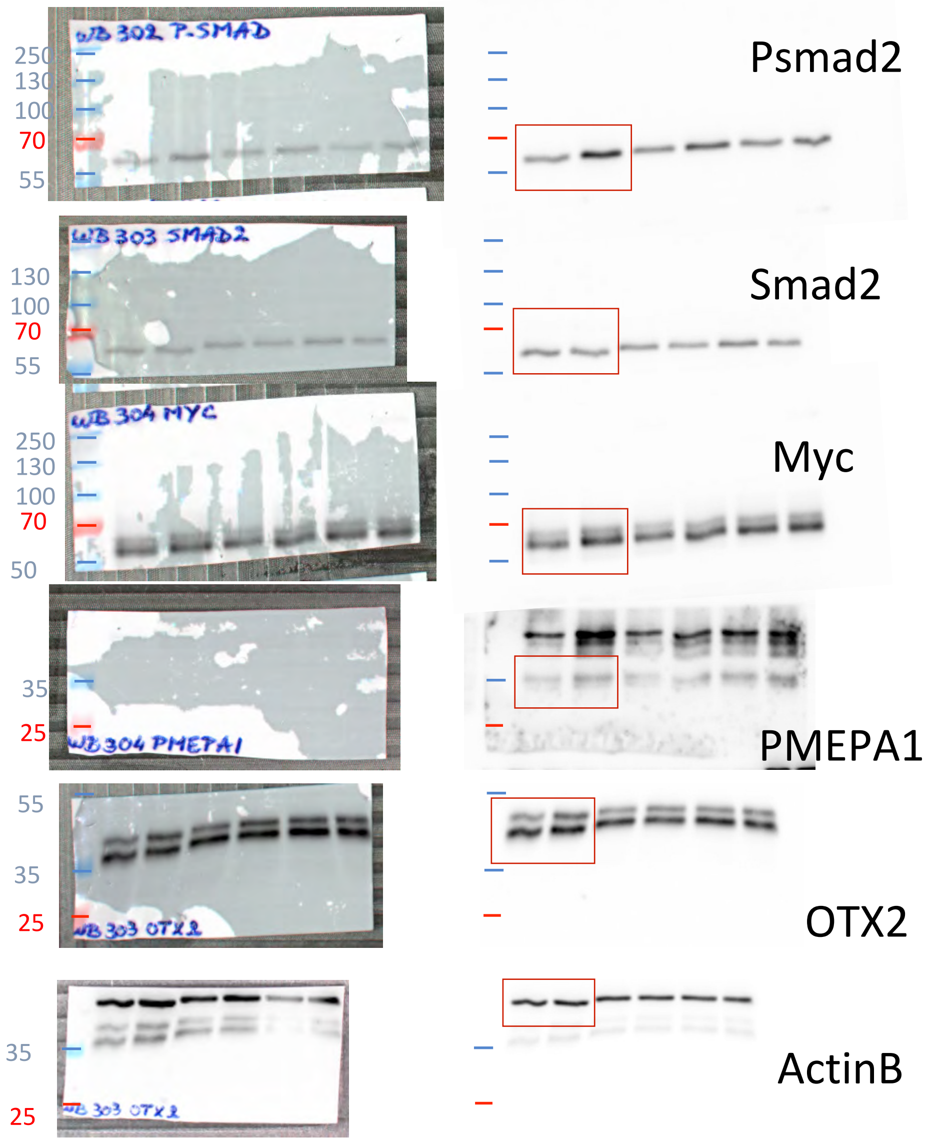

PDX7_DLS_WB40

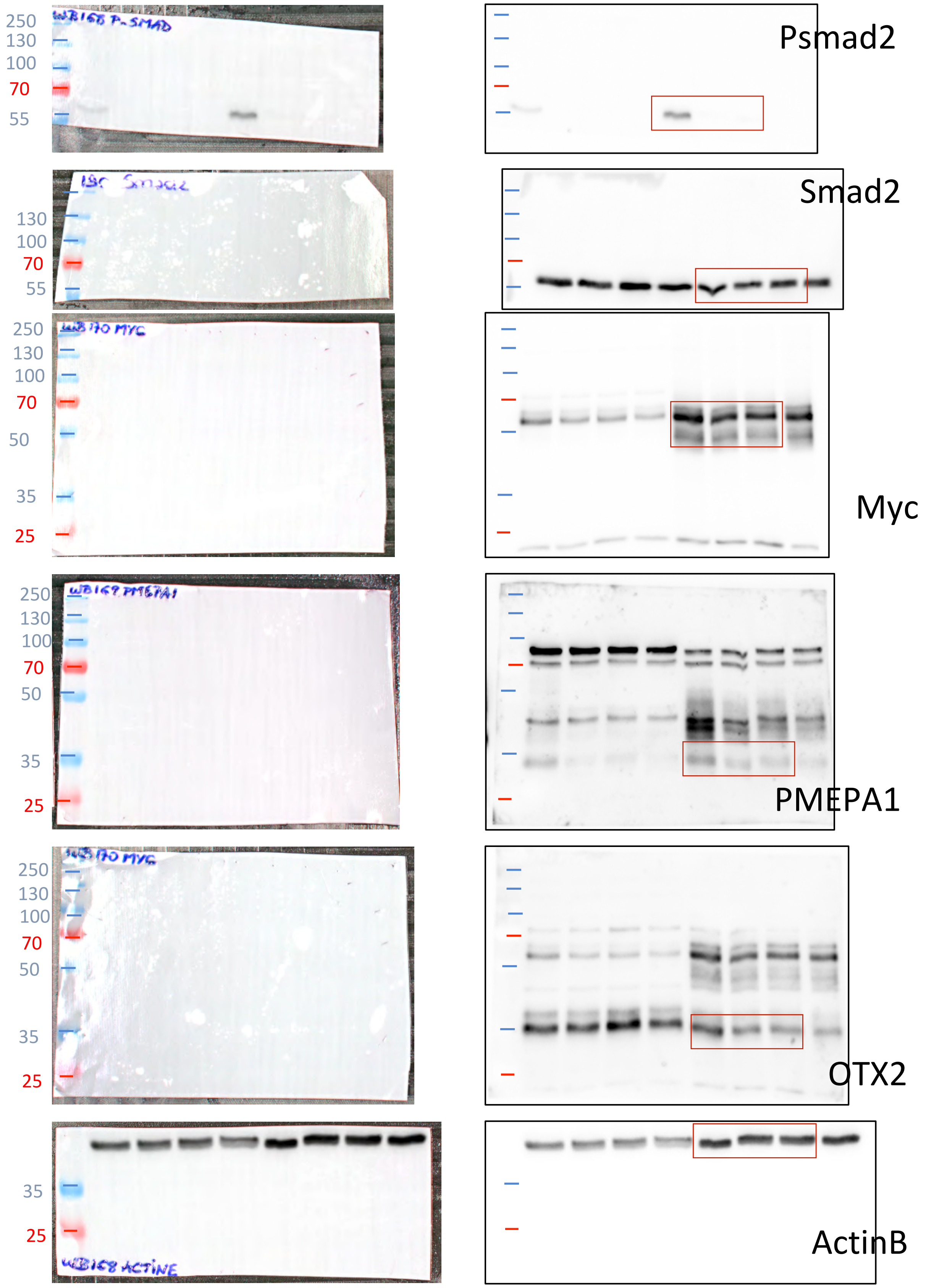

PDX7_PA_WB40

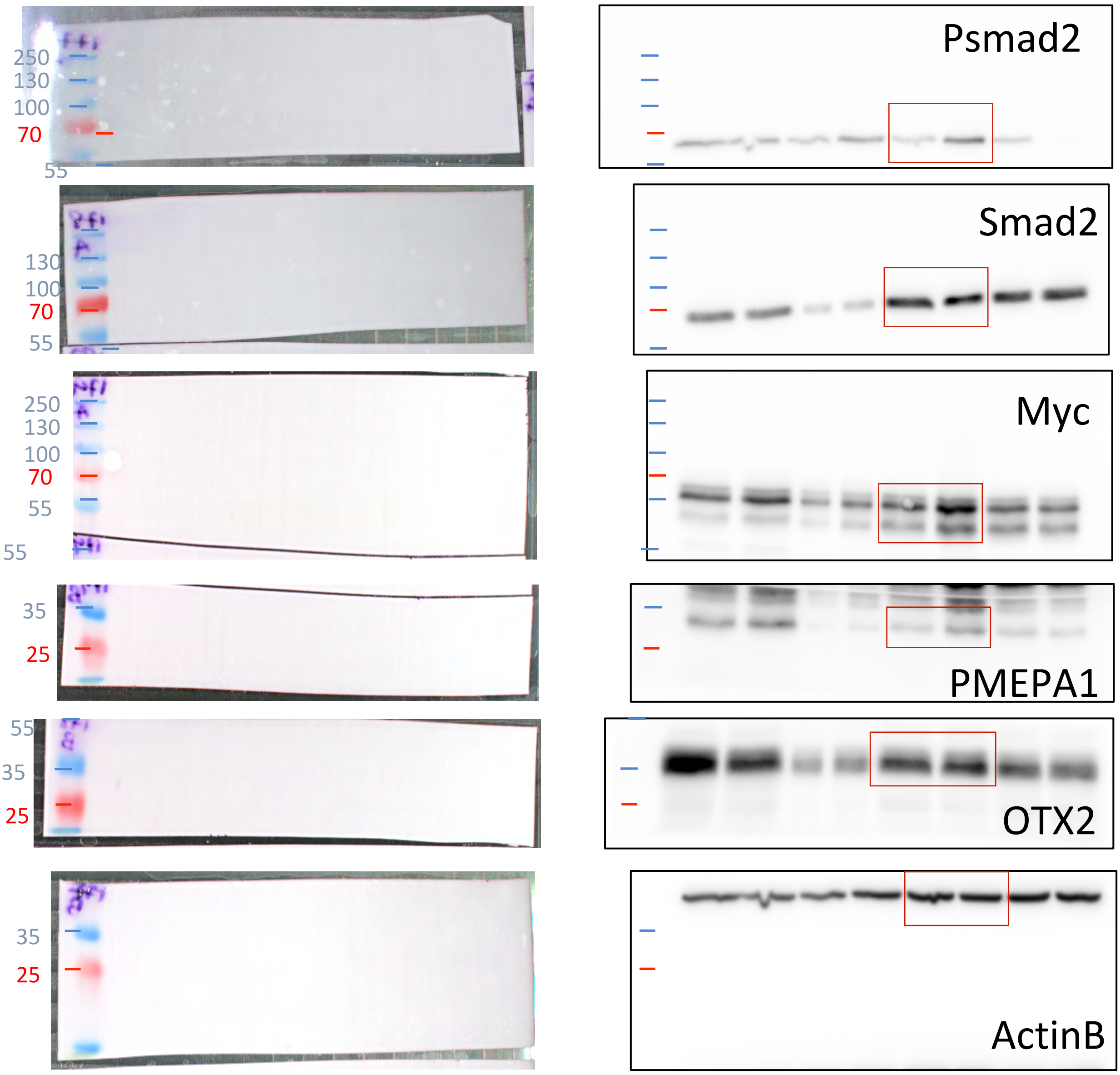
250
130
100
70
55
Psmad2
130
100
70
55
Smad2
250
130
100
70
55
55
Myc
35
25
PMEPA1
55
35
25
OTX2
35
25
ActinB
